# Benchmarking molecular representations and machine learning algorithms for asymmetric catalysis: a palladium-catalysed decarboxylative asymmetric allylic alkylation case study

**DOI:** 10.1186/s13321-026-01236-z

**Published:** 2026-06-12

**Authors:** Eduardo Aguilar-Bejarano, Declan Galvin, David M. Rogers, Ender Özcan, Simon Woodward, Patrick J. Guiry, Grazziela Figueredo

**Affiliations:** 1 School of Chemistry, University of Nottingham, University Park, Nottingham, NG7 2RD UK; 2 GSK Carbon Neutral Laboratories for Sustainable Chemistry, University of Nottingham, Jubilee Campus, Triumph Road, Nottingham, NG7 2TU UK; 3 School of Computer Science, University of Nottingham, Jubilee Campus, Nottingham, NG8 1BB UK; 4 School of Chemistry, Centre for Synthesis and Chemical Biology, University College Dublin, BelfieldDublin, D04 N2E5 Ireland; 5 School of Medicine, University of Nottingham, University Park, NottinghamNottinghamshire, NG7 2RD UK

**Keywords:** Asymmetric catalysis, Benchmarking, Explainable AI, Machine learning, Substrate screening

## Abstract

Machine learning (ML) applied to metal-ligand asymmetric catalysis remains less explored compared to other applications, such as drug design or materials science. Current strategies frequently focus on augmenting pre-existing descriptors (e.g. those originally formulated for medicinal chemistry), the development of new bespoke steric and electronic descriptors, and the use of molecular fingerprints. Such method diversity, in the absence of user guidelines, makes selecting optimal ML tools to model new asymmetric catalysis problems challenging. This is exacerbated in early asymmetric catalysis metal-ligand development, where typically only limited data describing how ligands and substrates affect reaction stereoselectivity are available. Herein, we present a benchmarking pipeline for asymmetric catalysis ML studies and a comparative evaluation of reaction representations, including bespoke electronic and steric descriptors, fingerprints (CircuS and Morgan), physicochemical descriptors (from RDKit), machine-learnt representations (ChemBERTa, MolT5, and UniMol), and graph representations, paired with a range of ML algorithms (Support Vector Regressors, Random Forests, Extreme Gradient Boosting, Multilayer Perceptron, and GNNs). We use a database comprising only 103 early-development palladium-catalysed decarboxylative asymmetric allylic alkylation (DAAA) exemplars (three Trost-type ligands and 54 substrates) to predict reaction enantioselectivity, evaluating performance in low-data regimes and in extrapolative tasks under both random and substrate-scaffold splitting. An external validation dataset comprising 19 more recently developed reactions is used as a final evaluation. Across data regimes, Morgan fingerprints, ChemBERTa, and the bespoke V4 descriptors emerged as the most robust representations, with Random Forest the most consistent algorithm; on truly unseen substrates, an ensemble of GNNs gave the most accurate predictions. Importantly, scaffold-based splitting was found to estimate real-world extrapolation performance more reliably than random splitting. Post-hoc explainability analyses (SHAP and integrated gradients) revealed that the bespoke and fingerprint representations provided chemically meaningful insights into the substrate and catalyst features driving enantioselectivity, whereas embedding- and RDKit-based representations did not. Lastly, the methodology was validated on asymmetric hydrogenation and palladium-electrocatalysed C–H activation datasets, demonstrating its applicability to a wide range of asymmetric reactions.

**Scientific contribution**

A curated dataset of 103 palladium-catalysed decarboxylative asymmetric allylic alkylation (DAAA) reactions, drawn consistently from Guiry group publications, is introduced together with an external validation set of 19 newer reactions. A bespoke, interpretable descriptor strategy that combines fragment-level steric (van der Waals volume) and electronic (Hammett-derived) parameters is developed for this reaction class, requiring neither DFT computations nor experimental crystal structures. A systematic benchmarking methodology is proposed for evaluating combinations of molecular representations and machine learning algorithms under realistic conditions-including small training sets and chemical-space extrapolation-and is validated across three asymmetric catalysis datasets (DAAA, asymmetric hydrogenation, and C–H activation)

**Supplementary Information:**

The online version contains supplementary material available at 10.1186/s13321-026-01236-z.

## Introduction

Machine learning (ML) has aided chemical discovery in different domains [1–13]. Families of algorithms allow the mapping of a molecular computational representation to a property of interest by optimising a mathematical function in a process known as training. Once trained, the model can be used to predict the properties of molecules yet to be synthesised. Thus, instead of synthesising and testing all possible candidates in the chemical pool, the model can be used to predict the properties of an in silico library, focussing the experimental study towards those exemplars predicted to have optimal target variable outcomes. Different chemical industries and research areas have benefitted from ML technologies including: pharmaceutics [1–3], agroindustry [4–6], toxicological studies [7–9], material design [10, 11] and formulation optimisation [12, 13]. Asymmetric transformations catalysed by transition metal complexes have also benefitted from ML studies [14–18].

One of the most important aspects of modelling asymmetric transformations is the reaction representation used for ML model training [19]. In general, there are two types of representations: those that are mechanism-based and those that are mechanism agnostic [17]. The mechanism-based descriptors usually require of high-level computations (DFT) of the actual catalytic process [20–22]. While these representations have shown to be very reliable in reaction development, especially under the extremely low data regime, they require precise knowledge of the reaction mechanism and/or its transition states, which can be very time consuming to attain. On the other hand, mechanism-agnostic representations usually represent just the starting materials and map these to reaction outcomes [23]. While there is no explicit mechanistic information, the representation aims to contain all necessary structural information that explains the reaction selectivity. Examples of these representations include fingerprints [24, 25], molecular descriptors [26, 27], molecular graphs [28, 29], and bespoke electronic and steric descriptors [30].

Exceptional studies presenting different descriptor generation strategies for asymmetric catalysis modelling are found in the literature. Li et al*.* developed steric- and electronics- embedded molecular graph (SEMG) [31], a graph representation aware of 3D information from DFT optimised structures (mechanism agnostic approach), which in combination with the Molecular Interaction GNN (MIGNN, developed in the same study) showed great predictive power of enantioselectivity on the dataset of thiol addition to *N-*acylimines by Denmark and coworkers (1075 datapoints) [32]. Cuomo et al*.* showed that neural networks trained on transition state (TS) DFT-derived descriptors can lead to good performance, even when the data available is very limited (17 training samples were used in this study). Exceptionally, Sigman and coworkers have reported a descriptor generation strategy that accounts for changes in the enantiodeterming step with changes on substrate and/or catalyst, showing excellent performance on chemical space extrapolation (prediction of enantioselectivity of unseen substrates/catalysts) [33]. Other relevant studies are also found in the literature [34–38].

Recently, Zhou and co-workers demonstrated how different molecular representations in combination with different ML algorithms can impact the trained model performance for the prediction of different DFT derived molecular properties for small organic molecules [39]. Similar studies are also found in enantioselectivity studies. For instance, Hong and coworkers explored the performance of multiple representation strategies (including RDKit descriptors, molecular fingerprints, atom-centred symmetry functions, local many-body tensor representation, and many-body tensor representation) in combination of multiple ML algorithms (AdaBoost, bagging, decision tree, extra-trees, neural network and XGBoost), leading to insights of what representation-ML algorithm pair lead to the best performing models predicting enantioselectivity of a database of 12619 asymmetric hydrogenation reactions [40]. Similarly, in a study from the same research group, it was demonstrated that transfer learning approaches can facilitate knowledge transfer from a well-documented Pd-catalysed Suzuki-Miyaura Cross-Coupling (SMC) reaction (310 reactions curated from the literature) to a novel Ni-based atroposelective SMC, where different descriptor approaches (molecular fingerprints, RDKit descriptors, physical-organic descriptors, atom-centred symmetry functions, and many-body tensor representation), with different ML algorithms (extreme gradient boosting, light gradient-boosting machine, random forest, extra trees, and decision trees) were evaluated [41]. Similarly, Hoque et al*.* collected 376 reactions [42], containing three different alkene transformations (cyclopropanation, aziridination, and arylation) and modelled them using an AttentiveFP-Cl model (GNN style model developed in this study), which showed enhanced performance compared to one-hot-encoding of molecules (e.g. catalyst, substrate, and ligand) present in the reaction (modelled with a Neural Network), molecular fingerprints (modelled with a Neural Network), SMILES (modelled with T5Chem [43], ULMFiT [35], and Transformer [44]), and graph representations (modelled using a MPNN [45]).

**Fig. 1:**
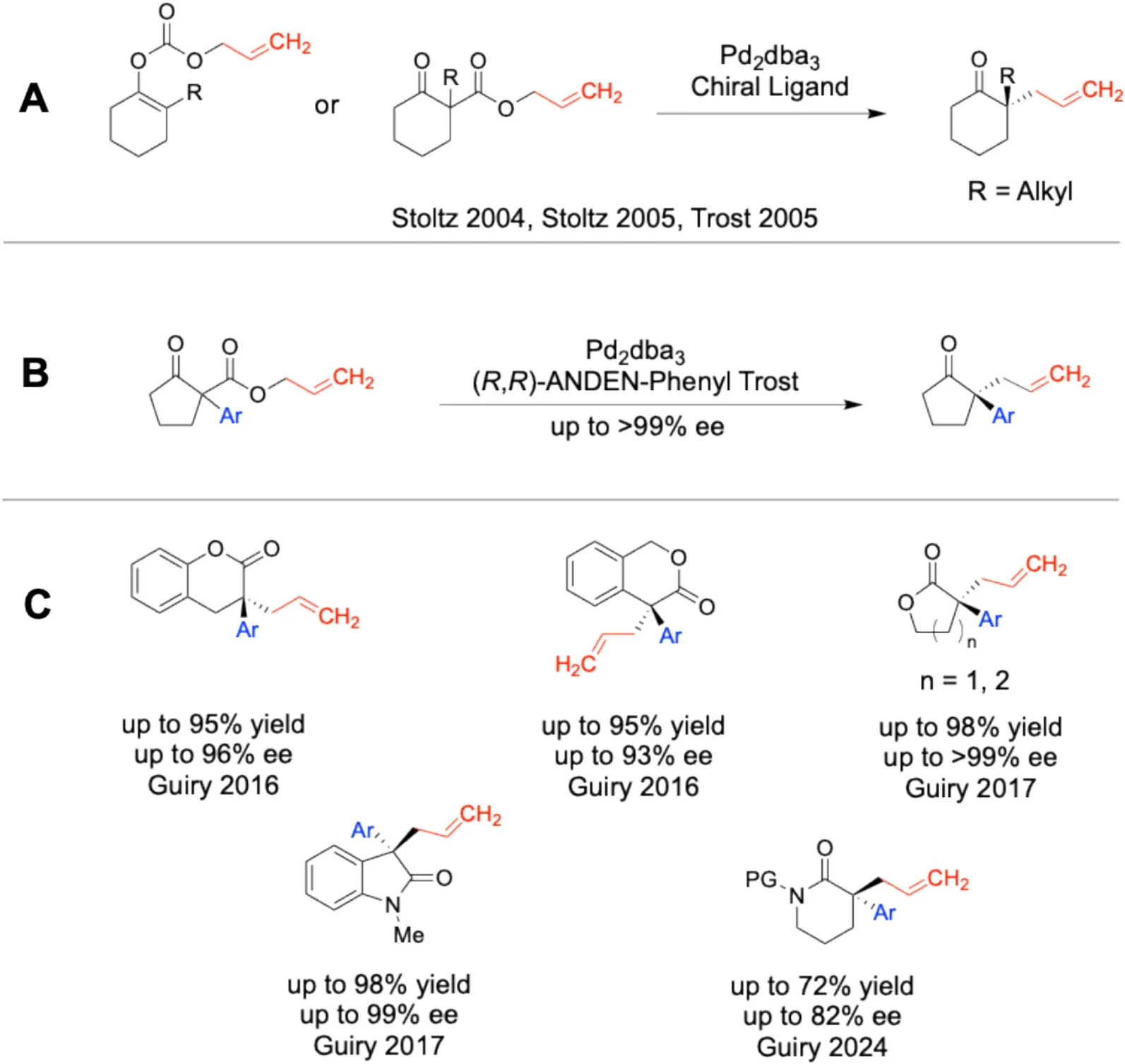
**A** Traditional α-alkyl DAAA reactions (2004–05). [50–52] **B** Initial α-aryl DAAA reaction reported by Guiry. [53] **C** Recent motifs accessible by α-aryl DAAA reactions. [54–57] dba stands for dibenzylideneacetone; the structures of the chiral ligands are discussed later

While we recognise the valuable contributions made in this field, these approaches may face certain challenges when applied to real-world catalyst optimisation processes. First, many of these studies rely on relatively large datasets (> > 100 reactions), which may be less representative of the early lab stages of reaction optimisation [31, 34, 40, 42]. Second, several of these studies adopt random splitting for model evaluation, which might not fully capture how a model performs when exploring new chemical space (either substrates or catalysts) [27, 34, 40, 42]. Third, a number of these studies depend on prior knowledge of the transition state and reaction mechanism [33, 36, 46], which is not always readily available, particularly for recently discovered or newly developed reactions. Finally, such benchmarking can sometimes give less attention to the interpretability of descriptors, an aspect that can be especially valuable for synthetic chemists seeking to understand the underlying chemistry of a reaction. Here, we fill this gap in the literature by systematically comparing various representation-model pairs using different train/test splitting strategies (random and substrate-scaffold based) in the context of a common asymmetric catalytic reaction: palladium-catalysed decarboxylative asymmetric allylic alkylation (DAAA, Fig. 1) [47].

The DAAA reaction is a mild, but powerful tool for the synthesis of compounds possessing α-quaternary stereocentres [48, 49]. The DAAA reaction has evolved to give both high yields and enantioselectivities when using allyl enol carbonates and β-keto allyl ester substrates (Fig. 1A) [50–52]. While the enol/ketone and allyl substitution patterns have been extensively varied, the identity of the α-substituent (R in Fig. 1A) has received much less attention. Normally, this R group is a compact alkyl group such as a methyl or ethyl, or a benzyl group. In 2016 Guiry and co-workers expanded the scope of DAAA reactions to include bulky α-aryl cyclopentanones (Fig. 1B) showing high yields and excellent enantioselectivities (> 99% *ee*) [53]. These studies could be generalized to other α-aryl containing related substrates (Fig. 1C) [54–57].

Despite the insights that ML can provide in chemistry, its uptake by mainstream synthetic chemists has been slow, due in part to limited awareness and easily applied approaches. Here we compare simple and effective methodologies for supervised ML modelling of the DAAA reaction. We use this to illustrate which ligand/substrate features lead to the best reaction selectivity predictions when only small data sets are available (e.g. at the onset of the development of a new asymmetric reaction). We compare a bespoke feature set (based on fragment volumes and electronic factors) [30] against two molecular fingerprints (Morgan fingerprints and CircuS fingerprints) [24, 58, 59], physicochemical molecular descriptors (calculated using RDKit) [26, 27], embedding representations from foundation models (ChemBERTa [60], MolT5 [61], and UniMol [62]), and graph representations [28]. All five representations were used to predict reaction enantioselectivity, for the same set of Pd-catalysed α-aryl DAAA reactions, to highlight substrate/ligand features that lead to high enantioselectivity and with the ultimate goal of providing insights of considerations to take when choosing a molecular representation in different scenarios, including small training datasets and extrapolation of chemical space.

## Methods

### Datasets

A total of 103 DAAA reactions of α-aryl containing substrates were gathered from this reaction’s initial experimental development [53–57]. These reactions were compiled into a single dataset, the *benchmarking* dataset. This dataset contains information about the substrate, ligand and solvent of the reaction represented by their SMILES string. Further, information about the product structure, the product chirality, the time of the reaction, the temperature, the yield and the enantiomeric excess (ee) is also available. The purpose of this dataset is to evaluate each model’s and representation’s performance in different scenarios, such as changing the size of the training data and extrapolation tasks based on the substrate scaffold.

Further, another dataset with the same information was built from later, more developed, DAAA reaction studies [63, 64], the *external validation* dataset (19 reactions). The purpose of this dataset was to evaluate the different models’ and representations’ abilities to predict stereoselectivity outcomes for real-world non-seen exemplars. While within the *benchmarking dataset* there is, of course, an internal test set used for evaluation, it is argued that these testing exemplars are so similar to those in the training set, that prediction into nearby (but less known) chemical space is, by definition, biased. By creating a new test set based on completely different catalysis studies, it is ensured that there is a wider gap in chemical space between training and test sets that can be used to evaluate the model’s robustness to extrapolate to genuinely new (unseen, but in nearby chemical space) reactions. We aimed to answer the question of how useful existing ML models/data would have been if these were used as a guide to predict into this new chemical space: a typical experimental question in the development of new asymmetric reactions.

To further validate our proposed framework, we included studies of two extra asymmetric catalysis datasets collected from the recent literature. First, the asymmetric hydrogenation dataset from Singh et al. was studied [27]. This dataset contains a total of 368 asymmetric hydrogenation reactions catalysed by BINOL-phosphite, BINOL-phosphoramidite, BINAP, BINAP-O, and BINOL-phosphoric acid catalysts families. The dataset encompasses a total of 59 ligands, 178 substrates and 7 solvents. The palladium-electrocatalysed C-H activation of biaryl systems dataset from Xu et al. is also benchmarked [36]. This dataset includes a total of 127 reactions, with 12 biaryl molecules, 21 olefins, and 20 transient directing groups (TDG). Unlike the DAAA datasets, we use these datasets to demonstrate that a general workflow for ML benchmarking studies in asymmetric catalysis is established. For space reasons these additional validations are partially confined to the Supporting information, with a majority focus on the DAAA studies here.

### Molecular representations

For our studies, five different representations families were used to encode the molecules: (1) physicochemical molecular descriptors [65], (2) molecular fingerprints (Morgan fingerprints [58] and CircuS fingerprints [59]), (3) graph representations [28], (4) machine-learnt representations from transformer models (ChemBERTa [60], MolT5 [61], and UniMol [62]) and (5) bespoke steric and electronic descriptors [30]. The aim is to compare these representations and their performance in predicting the enantioselectivity of unseen reactions in different circumstances, including machine learning algorithm used, the number of training datapoints, and the similarity of molecules in the training and test set.

The participant molecules in each reaction (substrate, ligand, and solvent for DAAA and asymmetric hydrogenation datasets and biaryl substrate, olefin, and TDG used for the C-H functionalisation dataset) were transformed into their SMILES strings. In the case of the molecular descriptors, all the available descriptors from RDKit on the rdkit.Chem.Descriptors module were calculated for the different molecules (see Supporting Information RDKit physicochemical descriptors section) [65]. Morgan fingerprints were calculated for each molecule from their SMILES string using the RDKit package, with a radius of 2 and 1024 and 2048 bits, allowing us to test bit size on model performance (see Supporting Information Morgan fingerprints section) [58, 65]. Lastly, CircuS descriptors were calculated using the DOPtools package [66], setting a lower value of 0 and a radius of 2 (see Supporting Information CircuS fingerprints section). The reaction representation was obtained by concatenating the descriptors/fingerprints of the participant molecules in a single fixed-length reaction-level vector representation.

In the case of graph representations, graphs were built using RDKit. To do this SMILES strings were converted into Mol objects. Graphs for each reaction component were created by iterating through the atoms within the Mol object, obtaining information on their connectivity (shared bonds) and atomic properties, including element identity, the number of neighbouring atoms (atom degree), hybridisation, formal charge, chirality, aromaticity, and if they are part of a cyclic structure. Edges were featurised with bond properties, including bond type, bond stereochemistry, if it was part of a ring, and if it was conjugated. Finally, the reaction representation was created by concatenating the three graphs into a single reaction graph. Further details are given in Supporting Information Graph representation section.

For embedding-based representations, numerical representations were obtained from the canonicalized SMILES string. In the case of the ChemBERTa [60] and MolT5 [61] models, these were sourced from the HuggingFace repository [67]. With the model loaded, the canonical SMILES were inputted to the network, and the last hidden states of the model-generated embedding were obtained. As each token receives its own embedding, the molecule was represented by the average of the hidden states of all the tokens within the SMILES string (see Supporting Information ChemBERTA and MolT5 sections for further details). For the UniMol representation, the unimol-tools package was used [62]. From the package, the UniMolRepr class was used to get the molecule representation from the SMILES strings. The reaction representation was the concatenated embeddings of the participant molecules. See Supporting Information UniMol section for details.

**Fig. 2:**
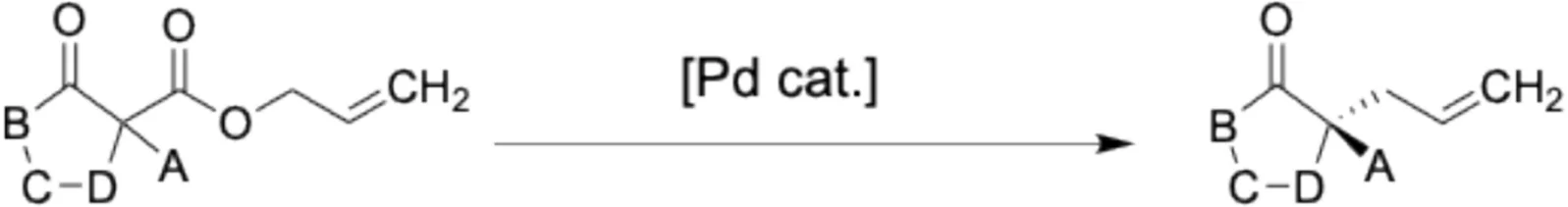
Fragmentation approach used to featurise DAAA substrates. For example, the left most structure in Fig. 1C is defined this way as containing the fragments: *A*  Ar, *B*  O, *C ortho*-C_6_H_4_, *D *CH_2_

**Fig. 3:**
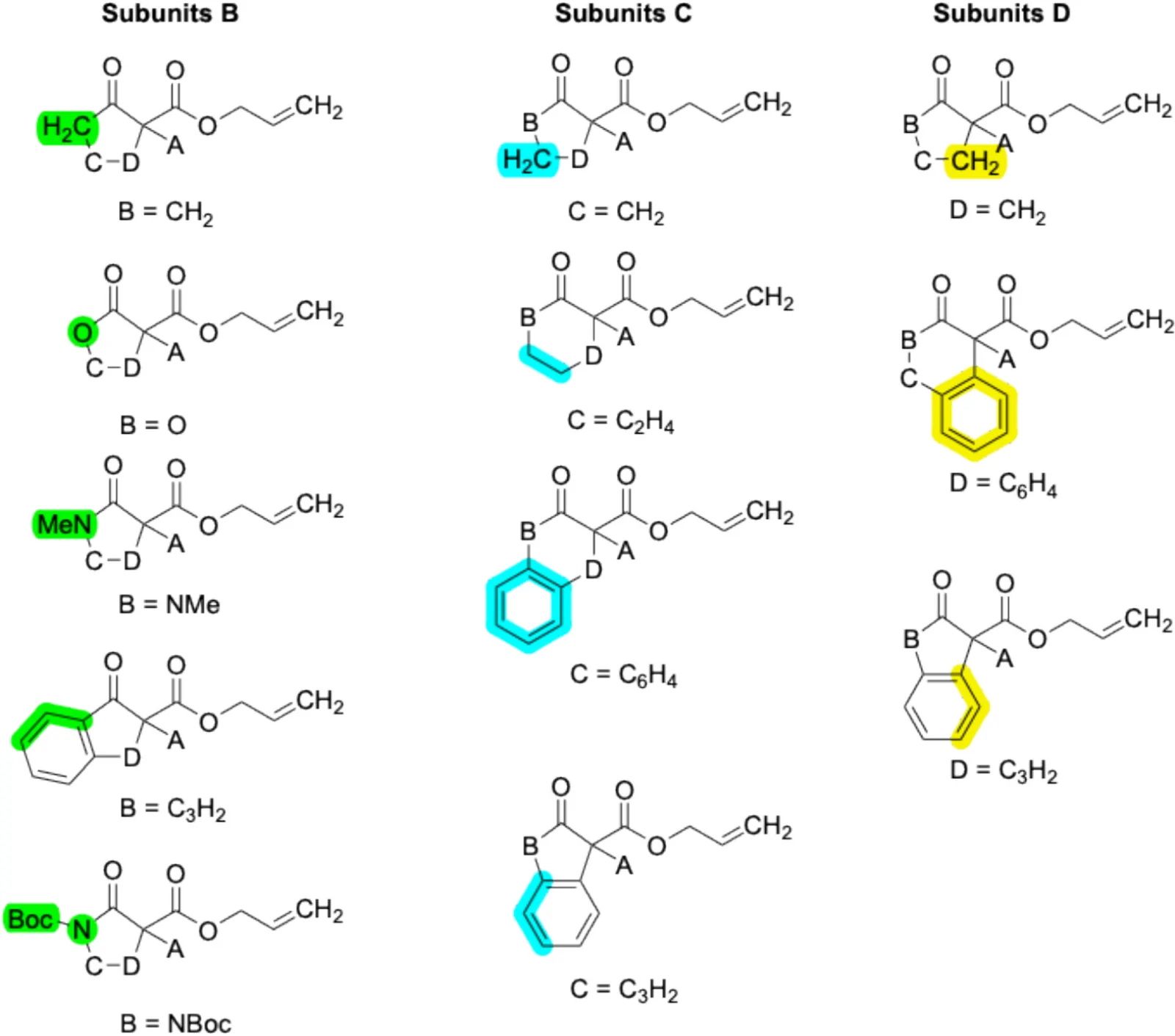
Structures of subunits B, C and D found the in the DAAA benchmarking dataset

For the bespoke strategy in the DAAA datasets, we aimed to encode the substrate and the catalyst fragments so as to maximise interpretability of the ML results (for use in onward reaction development) [57]. To attain this the core of the DAAA substrates are broken down into elements A-D (Fig. 2), using the approach of Owen [30]. To minimize the descriptors needed for featurization, the structural units B-D were approximated just by their van der Waals volume (as calculated by the method of Zhao [68]) and electronically estimated by a Hammett parameter. The Hammett parameter has been taken from a survey [69]. In cases where the Hammett parameter is not known (for example for –CH_2_–CH_2_– it is impossible to determine this experimentally), we took the closest analogue, which in all the cases consisted in adding a hydrogen to the subunit (e.g. CH_3_–CH_2_–). While the addition of this hydrogen changes the identity of the subunit, the electronic contribution of the extra hydrogen atom is likely to be negligible. The subunits B, C and D present in the dataset are summarized in Fig. 3.

**Fig. 4:**
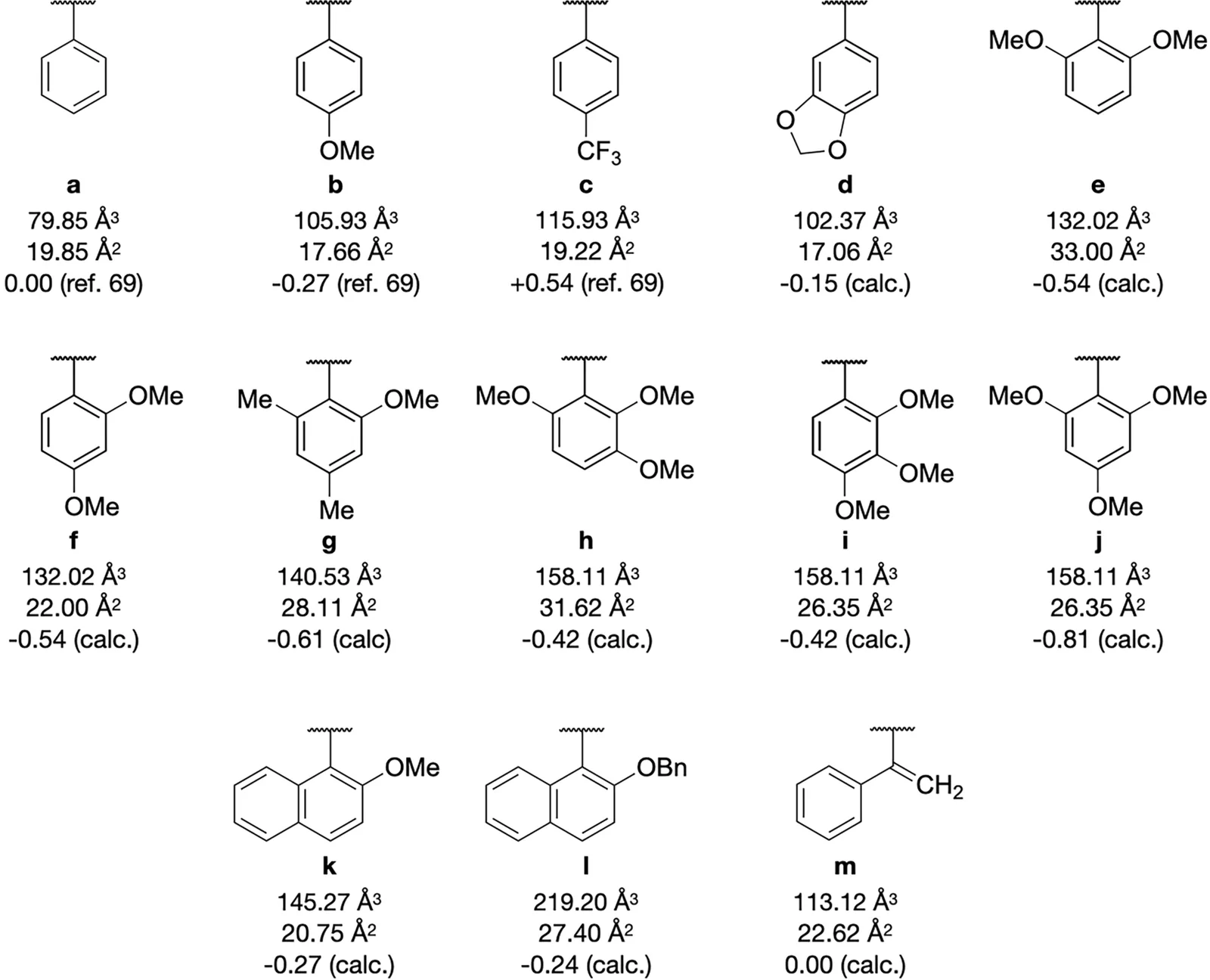
Structures of subunits A in the dataset. The calculated [68] fragment volumes used are given in Å^3^ first, then their ‘stoutness’ (in Å.^2^), and finally the actual, or calculated Hammett parameter using the method V2 (see text and Supporting Information “Calculated Hammett Parameters for Fragment A” Section)

The structural unit A denotes the α-aryl substituent, whose electronic and steric factors have significant impact on the enantioselectivity of the DAAA reaction. We chose to represent the bias in the steric demand of this type of fragment by its ‘stoutness’ (van der Waals volume over longest perpendicular chain length, as proposed by *Owen *et al*.*) [30] and its electronics by Hammett parameters. The 14 aryl fragments (a-m) present in the dataset used are shown in Fig. 4. The used Hammett parameter [69] of a is + 0.00 by definition (i.e. that derived from benzoic acid), only two other substitution patterns have experimentally derived Hammett values (fragments b and c). Therefore, we approximated the Hammett parameter of the ten remaining examples (d-m) by four different methods, discussed in depth in Supporting Information “Calculated Hammett Parameters for Fragment A” Section.

**Fig. 5:**
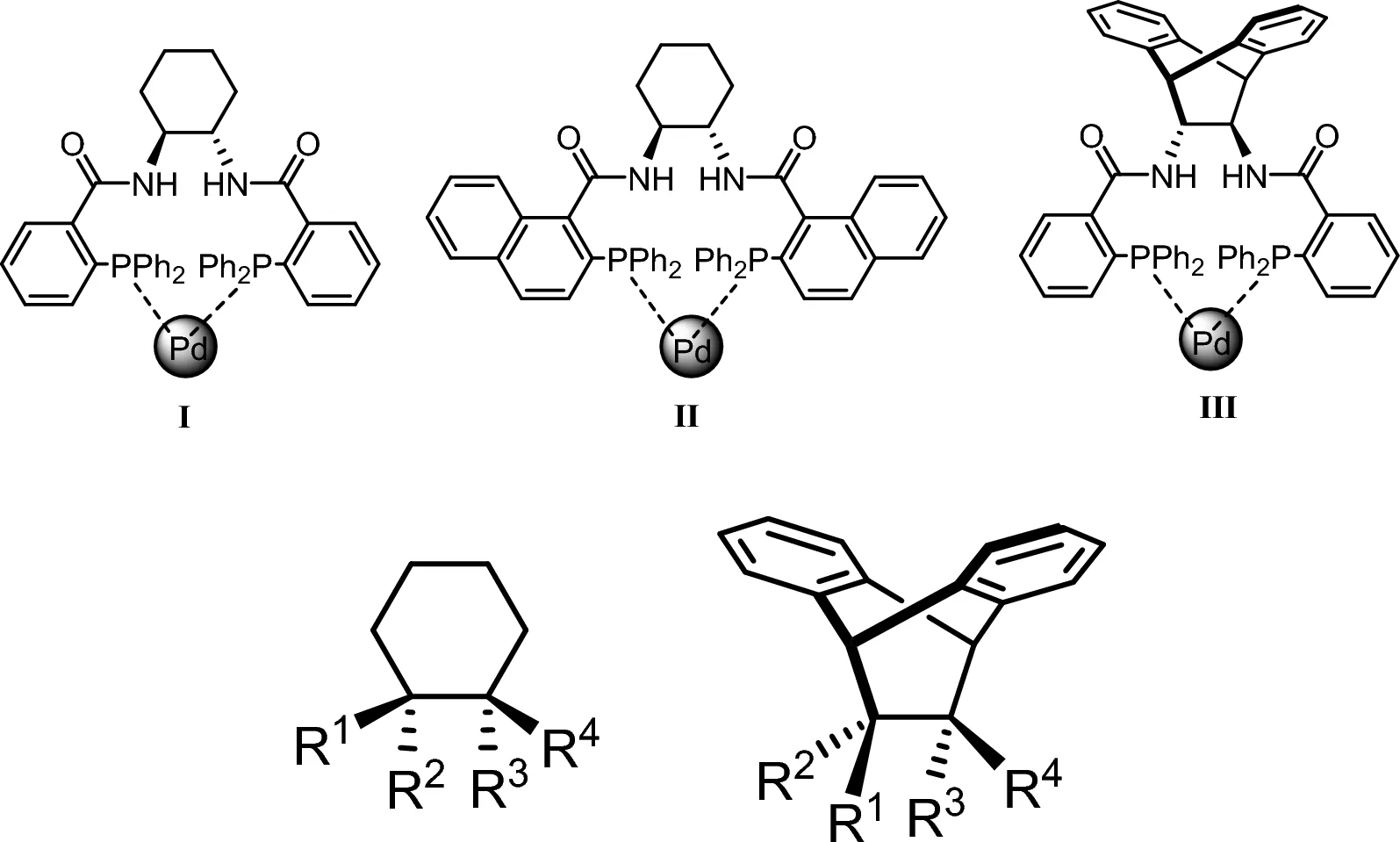
Structure of the three Trost ligands present in the dataset and the fragmentation approach taken for their featurisation here

Which enantiomer of the DAAA product is formed is controlled by the ligand, e.g. the Trost ligand chirality (*R*, *R*) vs (*S*, *S*). In our DAAA transformation dataset, three different Pd-complexes are represented (**I-III**, Fig. 5). The solution behaviour of these catalysts is complicated by association phenomena [70], and it was not until Arseniyadis and Leitch (2023) reported the preparation of chiral, air stable, and reliable Pd (0) pre-catalysts for asymmetric allylic alkylation chemistry, including DAAA using Trost-type ligands, that a crystal structure became available [71]. Given the challenges faced in getting this crystal structure [71], equivalent problems likely co-exist for new catalysts, so we decided to create a bespoke representation for such catalysts that neither requires extensive DFT computations nor relies on challenging experimental X-ray data that may not be available.

Thus, the Trost ligands were featurised based on the substituents (R^1^-R^4^) within the cyclohexane backbone (Fig. 5**)**. Due to C_2_ symmetry of the catalysts studied herein, only one of the two substituents was featurised (R^1^ in the case of **I** and **II** and R^2^ in the case of **III**, as adding R^3^/R^4^ information will not lead to new information). Then, using Zhao’s [68] approach, the steric burden (Å^3^) of the substituent was approximated. Due to low exemplar numbers we only featurised the steric profile of the chiral space around catalysts **I-III** and did *not* include any electronic description, as these Trost-type ligands all have similar electronic properties. To differentiate the ANDEN subunit in catalyst **III** from the cyclohexane subunit in **I** and **II**, a “ANDEN” descriptor was used, which one hot encoded the presence of this moiety. Finally, the solvent was encoded by its dielectric constant.

Each DAAA reaction is thus represented by: (i) a volume calculated by Zhao’s method [68] and Hammett parameter taken from a survey [69] for each of the subunits B, C, and D of the substrate (6 descriptors); (ii) the volume occupied for the catalyst’s (**I-III**) substituents at each position in Fig. 5 (1 descriptor); (iii) the ANDEN descriptor (1 descriptor); (iv) the dielectric constant of the solvent (1 descriptor); and finally (v) the subunit’s A stoutness and it’s electronic descriptor (2 descriptors), totalling 11 descriptors. As the electronic contribution of the subunit A has been approximated by 4 different methods (see Supporting Information, Calculated Hammett Parameters for Fragment A” Section), 4 different representations of each reaction were created (V1-V4), each one using a different method to approximate the values for A’s electronic descriptor, while the other descriptors were unchanged.

In the case of the asymmetric hydrogenation and C-H activation datasets, the bespoke descriptors generated in each original publication were utilised [27, 36]. Details on these descriptors are available on Supporting Information (Asymmetric hydrogenation bespoke descriptors and C-H activation bespoke descriptor sections).

To account for experimental variables in each reaction, these were concatenated to each representation created for each reaction for all datasets. For graph representations, these variables were concatenated to the graph embedding vector after the pooling operation of the GNN. In the case of DAAA and asymmetric hydrogenation, the temperature was added as feature. In the case of the C-H activation dataset, temperature and current were added as features.

### Target variable definition

**Fig. 6:**
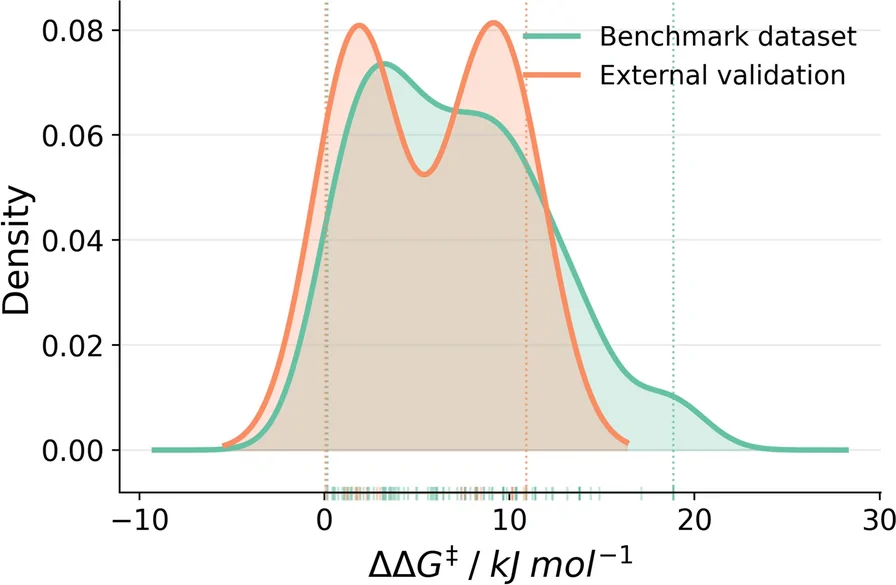
Distribution of ΔΔG^‡^ values in kJ mol^ −1^ for the benchmark and external validation datasets. The bimodal benchmark distribution is typical of initial chiral ligand/substrate experimental studies

While in the dataset there are exemplars with different facial selectivity outcomes, we decided to model the enantioselectivity of each reaction, regardless of the specific configuration of the product. The reason for this is that the selectivity is the most challenging optimisable outcome in asymmetric catalysis, while the specific product stereochemical configuration is trivially selected by the chiral ligand’s stereochemistry. Therefore, ΔΔG^‡^ (in kJ mol ^−1^) were obtained from enantiomeric excess values of the DAAA reactions collected from the literature (see Supporting Information “ΔΔG^‡^ target variable calculation” section to see how these values were calculated) [28, 30]. The value ΔΔG^‡^ is thus the difference in energy between the transition states providing different product stereochemical outcomes. The distribution of ΔΔG^‡^ values for the DAAA datasets are shown in Fig. 6.

In the case of the asymmetric hydrogenation dataset and the C-H activation dataset, ΔΔG^‡^ values are available from the original dataset sources. The distribution of values is shown in Figure S2.

### Data splitting

The aim of this study was to evaluate different representations and machine learning algorithms on different scenarios. Using the astartes package [72], the dataset was split into a train and test set. The astartes package does not only allow the partitioning of the data in a desired train/test ratio, but it also allows splitting based on molecular structure scaffolds. This way, the *benchmark* datasets were partitioned randomly (as usually done in ML evaluation protocols) and based on the substrate scaffold, with the goal of evaluating the model’s ability to extrapolate into chemical space and understand which protocol leads to more realistic evaluation of model performance.

### Data preprocessing

Before the ML modelling, the different molecular representations were pre-processed to ensure optimal training conditions. The preprocessing pipeline consisted of two steps: (i) feature selection and (ii) feature scaling. Feature selection eliminates non-relevant or redundant features that can ultimately lead to overfitting. For our studies, the first feature selection approach applied is a variance filter, in which the variance of each feature across the training set is calculated, and all features with 0 variance are eliminated. Then, a correlation filter is applied. In this filter, the Pearson correlation between all remaining features is calculated. If two or more features have a correlation greater than 0.95, all these features but one are eliminated. Lastly, the features were linearly scaled to values between 0 and 1 by using a min max scaler, using the formula:

$${X}_{scaled}=\frac{X-{X}_{min}}{{X}_{max}-{X}_{min}}$$

where *X* is the value of the feature, *X*_*min*_ is the minimum value of the feature found in the dataset and *X*_*max*_ is the maximum value of the feature found in the dataset. The modelled target variable training values were also scaled between 0 and 1 using the same approach.

After preprocessing had been applied to the training data, the test data were also transformed. For this, the same features that were eliminated on the training set were also eliminated from the test set. Lastly, the features are scaled using the same formula as the training set, meaning that the min and max values used were the same as those used in the training set.

### Machine learning algorithms and training

For the vectorial representations adopted (molecular descriptors, fingerprints, embedding representations, and bespoke descriptors), Support Vector Regressor (SVR) [73], Random Forests (RF) [74], eXtreme Gradient Boosting (XGB) [75], and MultiLayer Perceptron (MLP) [76] were evaluated. In the case of graph representations, Graph Neural Networks were used [77]. The decision of the use of these algorithms is based on their widespread use in cheminformatics for modelling of many molecular properties [78–82]. For all cases except GNNs, the hyperparameters of the models were tuned using a cross-validation approach on the train set. For this, the training set was split into 3 folds, a model was trained using 2 folds with randomly selected hyperparameters and tested on the remaining fold. This process was repeated until all folds have been used as test set. The set of hyperparameters that led to the lowest average mean absolute error for the three trials was used to train the final model on the totality of the training set and evaluated on the test set. In the case of GNNs, the architecture of HCat-GNet was adopted (details on architecture and training are given in Supporting Information “HCat-GNet architecture and training” section) [28], as hyperparameter optimisation would have led to significant computational cost. We also tested HCat-GNet with different convolutional layers, including Graph Convolutional Network (GCN, original implementation) [83], Graph Attention Networks (GAT) [84], and Graph Isomorphism Network (GIN) [85] to study effects of different convolutional layers on model performance. Details of GNN training and architecture are given in Supporting Information HCat-GNet architecture and training section.

As the models were trained on scaled target variables, the predictions were unscaled using the formula:

$$X={X}_{scaled}\bullet \left({X}_{max}-{X}_{min}\right)+{X}_{min}$$

### Benchmarking

As our goal is to evaluate a methodology (i.e. representation in combination with ML algorithm) and not individual models, we carried out benchmarking studies to analyse statistical significance on the results. To do this, all possible combinations of different parameters of molecular representations, ML algorithm, split sizes, and split types were generated. All possible options for each category considered are:

- Molecular representation: RDKit descriptors, Morgan Fingerprints radius 2 and 1024 bits, Morgan Fingerprints radius 2 and 2048 bits, CircuS fingerprints radius 2, graph representation, ChemBERTa, MolT5, UniMol, bespoke V1, bespoke V2, bespoke V3, and bespoke V4.
- ML algorithm: GCN, GAT, GIN, SVR, RF, XGB, and MLP.
- Splits size: 8/2, 6/4, 4/6 train/test ratio.
- Split type: substrate scaffold, random.

In the case of graphs, these were only combined with GNNs for trials, and non-graph representations were only combined with non-GNN ML methods. Each unique combination of experimental settings was tested a total of 5 times using different random seeds. For performance analyses, the mean absolute error (MAE) was predominantly used for our analyses, while R^2^ information is also used to complement discussions.

### Statistical tests

Due to the unbalanced and partially crossed experimental design-where graph-based models (GNNs) are only defined for graph representations, while traditional machine learning models are evaluated across multiple descriptor-based representations-a hybrid statistical framework was adopted (see Supporting Information Statistical analysis section for details). This framework allowed us to test statistically how the GNNs compared vs the rest of ML algorithms and representations using a Linear Mixed Effects (LME) and then paired test the non-graph approaches using a Wilcoxon test. For the latter, Holm correction was applied to p-values to account for multiple comparisons.

### Final external evaluation

The DAAA benchmarking dataset was used to train models and then to predict the enantioselectivity of the external validation dataset. To do this, models were trained on the entire benchmark dataset and were used to predict the enantioselectivity values of the external validation dataset. This last evaluation aims to unveil how the molecular representation can impact model predictive power on truly unseen exemplars.

### Explainability

Explainability analysis can be used to understand the features driving reaction (stereo) selectivity and thus provide pointers to catalytic reaction mechanisms. Each of the models trained using the entire *benchmark* dataset were explained using SHAP as implemented in Python [86] for vectorial representations, and in the case of GNNs, integrated gradients [87] was used. In the case of the latter, integrated gradients lead to node feature level attributions, that, on their own, are not of chemical relevance. Thus, we estimate importance of fragments by summing the attributions for all node features for all the nodes belonging to a fragment. For definitions of fragments, we used the BRICS definition [88]. Further details are available on Supplementary Information Explainability section.

### Benchmark applicability to other asymmetric reactions

To demonstrate the broader applicability of the framework, we show benchmarking studies on two previously studied asymmetric catalysis datasets. These datasets come from dedicated studies to the development of highly informative descriptors for enantioselectivity prediction. In this regard, these were used as case studies of how the pipeline could be easily extended to other reactions that have different pre-defined bespoke representations and compare them against the in silico created representations (i.e. fingerprints, graphs).

## Results and discussion

### Evaluation of the different representation families

**Fig. 7:**
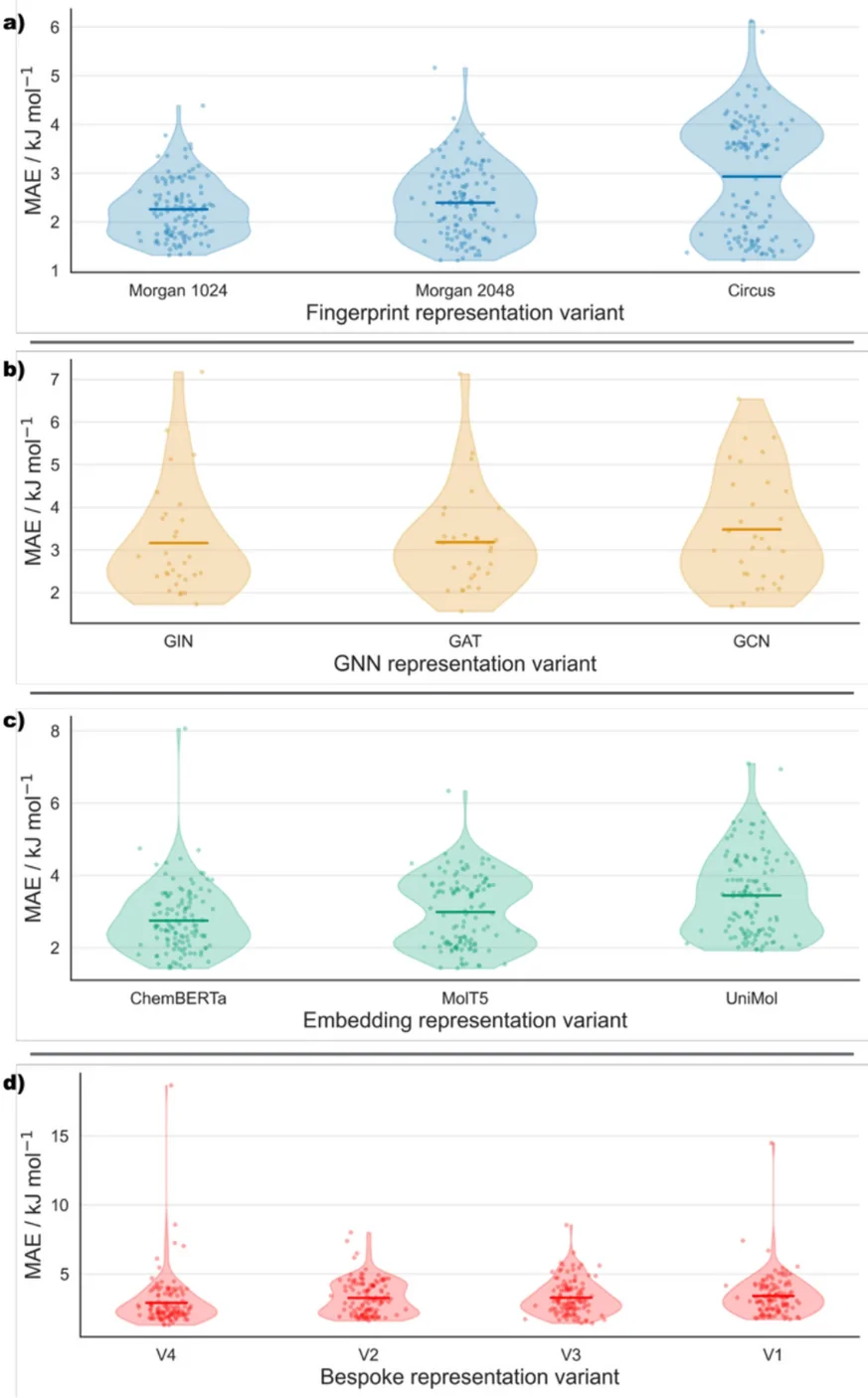
Distribution of MAE values in kJ mol^ −1^ for each representation tested. The plots are grouped by representation ‘families’, **a** molecular fingerprints, **b** GNN convolutional layers, **c** transformer embedding representations, and **d** bespoke strategies developed herein. Distributions in each plot are ordered by increasing MAE

First, the best representation among representation families was identified. To do this, the MAE of prediction of the ΔΔG^‡^ values from all the test sets from the different benchmarking experiments were studied. The MAE values were grouped by representation. The results are shown in Fig. 7.

In the case of the fingerprints, a total of 4 ML algorithms, 3 split sizes, 2 split types and 5 random seeds led to a total of 120 datapoints for each distribution. Tests showed statistical difference between all pairs of fingerprint representations evaluated. This result demonstrates that for this case study, the CircuS fingerprints are the least adequate for modelling purposes, with a MAE of 2.93 ± 1.17 kJ mol^ −1^. This result is rather interesting, as this fingerprinting approach was developed for asymmetric catalysis studies [59]. A possible reason for this is that these fingerprints require a training set to define the feature space (see Supporting Information CircuS fingerprints section), thus, leading to less transferable representations to new chemical spaces. In the case of the Morgan fingerprints, it was found that 1024 bits led to lower error (2.26 ± 0.59 kJ mol^ −1^) than the 2048 bits (2.39 ± 0.73 kJ mol^ −1^), possibly because of overfitting of the latter case. Morgan 1024 bits fingerprints were found to be the best representation for the fingerprint family.

For graph representations, instead of testing the representation, the convolutional layer of the GNN architecture is analysed. The total quantity of points for each distribution is 30 (3 split sizes, 2 split types and 5 random seeds). No statistical difference was found between any of the GNN models evaluated. This result suggests that the different architectures led to similar predictions for this case study. For further analyses, the GIN architecture was chosen because of its lower mean of 3.17 ± 1.29 kJ mol^ −1^, compared to GAT (3.18 ± 1.16 kJ mol^ −1^) and GCN (3.48 ± 1.32 kJ mol^ −1^).

In the case of the embedding-based representation family, statistical difference between all representations (p-value < 0.05 for all cases, n = 120) were found, showing that ChemBERTa leads to the most accurate models (MAE of 2.75 ± 0.73 kJ mol^ −1^). Interestingly, UniMol led to the worst performing models (MAE of 3.45 ± 1.14 kJ mol^ −1^). This representation is created from a transformer model trained on 209 million molecular conformations. A possible reason for its poor performance in this dataset is because it creates conformation utilising simple RDKit molecular dynamics-based optimisation, which may not be ideal for the molecules in this dataset. In this case, the sequential information derived from the 77 million SMILES on the ChemBERTa model seems to be richer in information for predictive tasks.

For the bespoke representations, statistical difference was found between all pairs of distributions (p-value < 0.05 for all cases, n = 120), except V2 and V3 (p-value = 0.24). The representation V4 was found to be the most accurate, with a MAE of 2.90 ± 1.88 kJ mol^ −1^. A hypothesis for this is that the electronic descriptor used in V4 is related to [13] C-NMR data, leading to experimental information about the electronic environment of the substrate. In contrast, V1 (3.41 ± 1.50 kJ mol^ −1^), V2 (3.26 ± 1.26 kJ mol^ −1^), and V3 (3.28 ± 1.25 kJ mol^ −1^) make use of approximations of the Hammett parameter, which leads to poorer representation of the molecules and worst performing models.

For the rest of the paper, we will only present results and discuss the best performing representations among families identified in this section: Morgan using 1024 bits, ChemBERTa, and V4 variant of the bespoke strategy. In the case of GNNs, analyses will focus on the GIN-based architecture. RDKit physicochemical descriptors are also included in next discussions.

### Model performance as a function of training set size

#### Random splitting

**Fig. 8:**
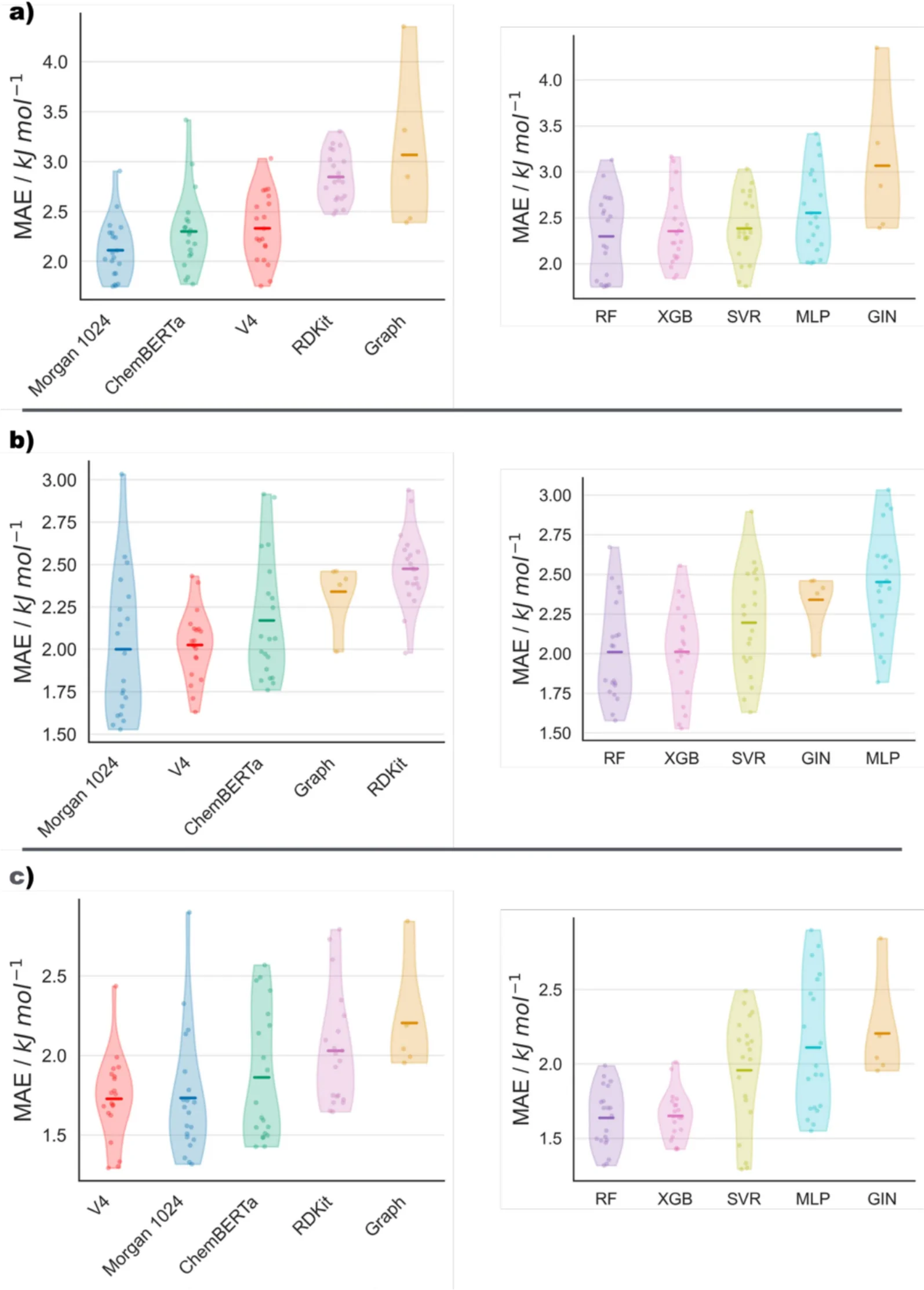
Distribution of MAEs of the test set over 5 random seeds for different representations and ML algorithms using random splitting for train/test splitting ratios of **a** 4/6, **b** 6/4, **c** 8/2. The plots on the left show the grouping based on feature, and plots on the right show grouping by ML algorithm. Distributions are ordered by increasing mean MAE value

Random splitting is typically used for ML model evaluation. To mimic scenarios with limited datapoints, we trained models on different train/test ratios (4/6, 6/4, and 8/2). The MAE distributions (Table S1 and Figure S3, see Table S2 and Figure S4 for R^2^ values) were grouped by representation and by model, with the goal of identifying if there was a representation or model that performed statistically better than the rest. The results are shown in Fig. 8.

For 4/6 train/test split ratio (Fig. 8a), pairwise statistical tests for representation distributions showed that only the pair of Morgan Fingerprints and V4 and the pair of ChemBERTa and V4 were not statistically different (*p-*values of 0.14 and 0.70 respectively, Table S3). From linear mixed-effects, it was found that graphs are not statistically different only with RDKit descriptors (p value = 0.22, Table S4). This result shows that the best representations for this train set size domain are Morgan Fingerprints, ChemBERTa, and V4. When analysing the model distributions, no statistical differences were found among non-GNN approaches (Table S5). In the case of GIN, statistical difference was found with all non-GNN algorithms (p-value < 0.05 for all cases, Table S6). These results suggest that GNNs are the worst algorithm to use in low data regimes. Furthermore, the fact that non-graph representations showed statistical difference while non-GNN algorithms did not, implies that the representation of the reaction is a more important factor for model performance than the ML algorithm used.

This procedure was repeated for a train/test set ratio of 6/4 (Fig. 8b, Table S7 for MAE values, and Table S8 for R^2^ values). As in the case for 4/6 splitting, all non-graph representation pairs were found to be statistically different except ChemBERTa and V4 and Morgan Fingerprints and V4 (p-values of 0.33 and 0.62 respectively, Table S9). The graph representation was found to be statistically different from Morgan Fingerprints and V4 (p-values of 0.03 and 0.04 respectively, Table S10). Among non-GNN approaches, MLP showed to be statistically different from the rest of approaches (p-value < 0.05, Table S11), being statistically the worst non-GNN algorithm. GIN was found to be statistically undistinguishable from MLP and SVR (p-values of 0.475 and 0.358 respectively Table S12). In comparison with the smaller 4/6 train/test splitting ratios, the graph approach starts having comparable performance to standard approaches with high-level features. Furthermore, with slightly more data, differences in model approaches start becoming noticeable, with the RF and XGB showing to be statistical better.

Lastly, for train/test ratio of 8/2 (Fig. 8c, Table S13 for MAE values and Table S14 for R^2^ values), it was found that the statistically different representations were Morgan Fingerprints and RDKit descriptors (p-value < 0.05, Table S15), graph and Morgan Fingerprints and graph and V4 (p-values < 0.05, Table S16). In the case of ML algorithms, MLP was found to be different from RF and XGB (p values < 0.05, Table S17), and SVR and XGB were also found to be different (p value 0.03). GIN was found to be statistically different from RF and XGB (p values < 0.05, Table S18). Similarly to the 6/4 train/test splitting scenario, the algorithm used becomes important in model performance.

In general, it is demonstrated that Morgan fingerprints, ChemBERTa and V4 are consistently the best representations across splitting sizes. The reason for this is that V4 and ChemBERTa approaches contain high level information derived from human-experts and machine-experts, thus, creating rich representations for the predictive task. In the case of Morgan Fingerprints, its good performance is likely due to the model learning to correlate molecular fragments with the selectivity outcome. As more datapoints become available, graph representations can attain comparable performance to traditional approaches, suggesting that in low data regimes it is susceptible to overfitting. RDKit is consistently found to be statistically worse in all cases, suggesting this representation is potentially less useful for this asymmetric catalysis studies. When analysing model distributions, in low data regimes, the ML used seems to be less critical for performance. However, as more data becomes available it may present a possibility for improvement. Particularly, the ensemble methods of RF and XGB were found to perform statistically better in higher number of training data points regimes.

### Scaffold-based splitting

**Fig. 9:**
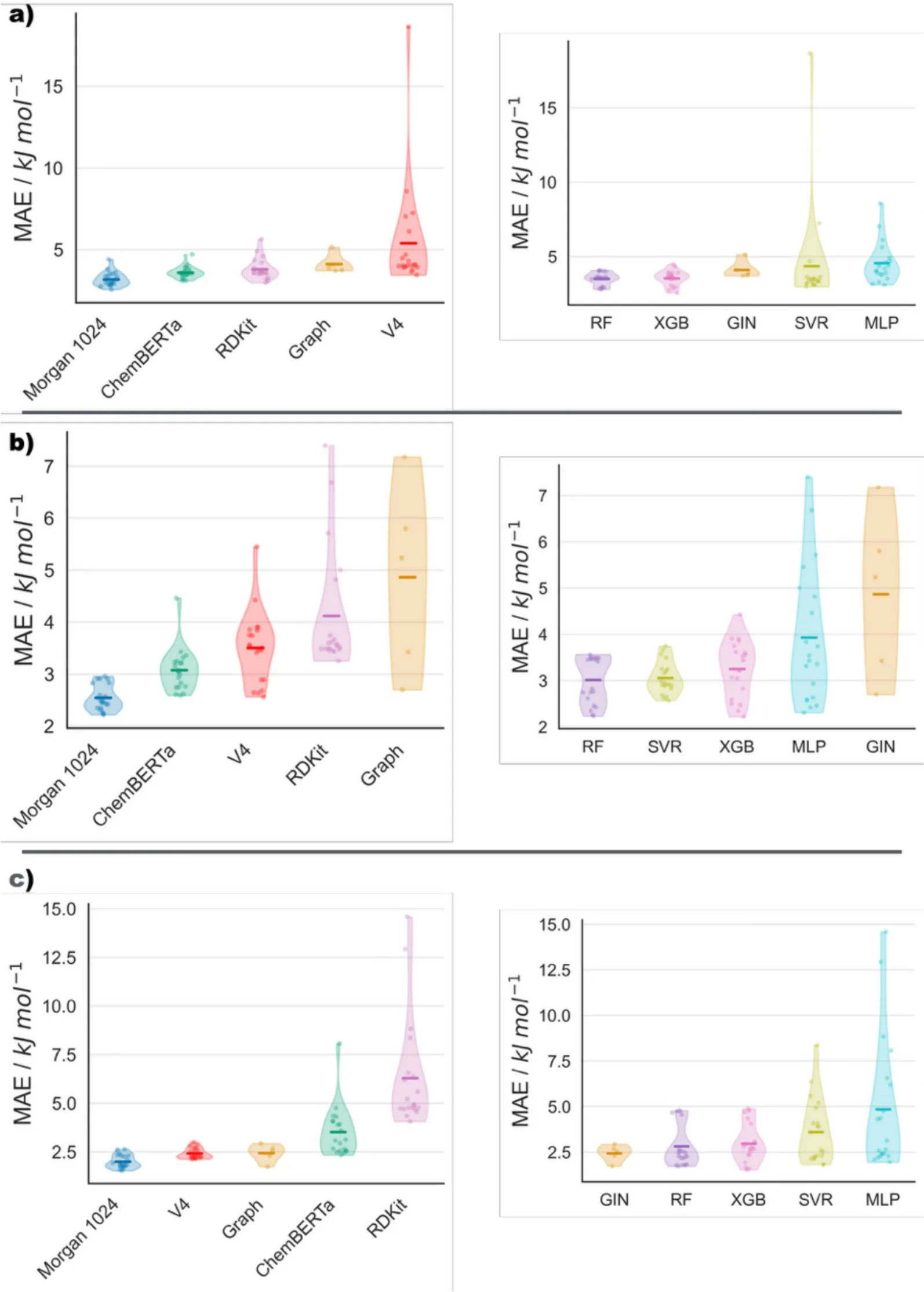
Distribution of MAEs of the test set over 5 random seeds for different representations and ML algorithms using scaffold-based splitting for train/test splitting ratios of **a** 4/6, **b** 6/4, **c** 8/2. The plots on the left show the grouping based on feature, and plots on the right show grouping by ML algorithm. Distributions are ordered by increasing mean MAE value

QSAR modelling allows exploration of unknown chemical space with the ultimate goal of optimising a target property. While most ML studies use random splitting to evaluate an ML pipeline or model, sometimes such approaches can be misleading regarding how the model will behave in extreme circumstances (e.g. predicting the properties of molecules whose structures are significantly different from those in the training data). Such extrapolation into chemical space is a core problem in asymmetric catalyst development, which ideally the ML model should help aid. In this study, we evaluate how the representation used impacts the model’s performance under simulated extrapolation tasks and ask how this compares with simple random splitting. The results obtained are shown in Fig. 9.

For scaffold-based splitting for 4/6 train/test ratio split (Fig. 9a, see Table S19 and Figure S5 for MAE values for different representation–ML algorithm pairs and Table S20 and Figure S6 for R^2^ values), it was found that only ChemBERTa and RDKit were not statistically different (p-value = 0.11, Table S21) among non-graph representations, while the graph representation was found to be statistically comparable to all representations (Table S22). By studying model distributions, it was found that only MLP was statistically different from all other non-GNN algorithms (p-value < 0.05 for all cases, Table S23), suggesting it performs the worst among ML models. GIN was not found to be statistically different from any other algorithm (Table S24). These results suggest that the ML algorithms are less critical for chemical space extrapolation in low data regimes, while representations have the highest impact on model performance. Further analyses of R^2^ distributions (Table S20 and Figure S7) revealed that only Morgan fingerprints always attained high R^2^ values for all the ML algorithms evaluated, while the other representations had only negative R^2^ when modelled with some ML algorithms, revealing this representation is robust for this low data extrapolation scenario.

For the 6/4 train/test ratio split (Table S25 and TableS26 for R^2^ values), it was found that all non-graph representations were statistically different between them (Fig. 9b, Table S27), while graph representation was found to be different from the rest of representations except RDKit (p-value = 0.07, Table S28). In the case of models, RF was found to be different from XGB and MLP (p-value = 0.001 for both cases, Table S29), while GIN was found to be statistically different from all other models (Table S30). The results suggest that the representation has more impact for model performance than the ML algorithm used. However, in this case, RF was found to be statistically better. Analyses of R^2^ values (Table S26 and Figure S7) showed that with this increase in training set size, all representations can attain positive values of R^2^, indicating increased predictive power on unknown chemical space compared to the 4/6 train/test scenario.

Lastly, train/test 8/2 MAE analyses (Fig. 9c, see Table S31 for MAE values and Table S32 for R^2^ values) revealed that all non-graph representations were statistically different between them (p-value < 0.05 for all cases, Table S33), while graph representation was only statistically different from RDKit (p-value > 0.05, Table S34). Among non-GNN methods, only RF and XGB showed no statistical difference (p-value = 0.11, Table S35). Interestingly, GIN showed to be the ML algorithm with the lowest error (Fig. 9c). However, analyses showed only statistical improvement compared to MLP (Table S36). This result suggests that, with enough data points (~ 80 points for this case study), GNNs can attain comparable performance to traditional methods, such as RF and XGB, in unknown chemical space extrapolation tasks. Furthermore, compared to the random splitting, in this case, the representation showed to have a statistical impact on the model performance regardless of the training set size, demonstrating the importance of robust representations for extrapolative tasks.

Compared to the random splitting, it was found that, for both tasks, Morgan is consistently the best representation. While ChemBERTa representation is among the best performing representations for random splitting, it showed poor performance on 8/2 train/test split size for the extrapolative task, likely due to overfitting. In the case of the bespoke V4 strategy, interestingly, it performed poorly for the 4/6 scaffold-based splitting. However, as the dataset size became bigger, it showed competitive performance, which is expected due to the domain expertise used to generate these high-level descriptors. Remarkably, graphs and GNN architectures showed to have competitive performance on extrapolative tasks when enough data becomes available, presenting an attractive representation in case that sufficient data points are available. Lastly, RDKit showed to be the poorest representation for all splitting methods and splitting ratios, implying that it is not suitable for this case study of asymmetric catalysis modelling. This demonstrates the importance of robust representations of reactions for ML modelling rather than focussing efforts on ML algorithm selection.

Lastly, we attempted to create train/test splits based on the target variable (ΔΔG^‡^) to study the capabilities of the models on extrapolating the values of the modelled variable. We found that correlations were poor (Table S37), suggesting that extrapolations with the ML algorithms and representations tested herein are rather challenging. This suggests that the model will be unable to predict accurately enantioselectivities higher than those seen during training. This way, in very early stages of reaction development programmes, it is useful to include exemplars with high enantioselectivities even though these lie in very different chemical space (such as chemically different substrates or catalysts), as our results indicate that models are more accurate extrapolating in chemical space than on target variable values.

### Performance on truly unseen exemplars

**Fig. 10:**
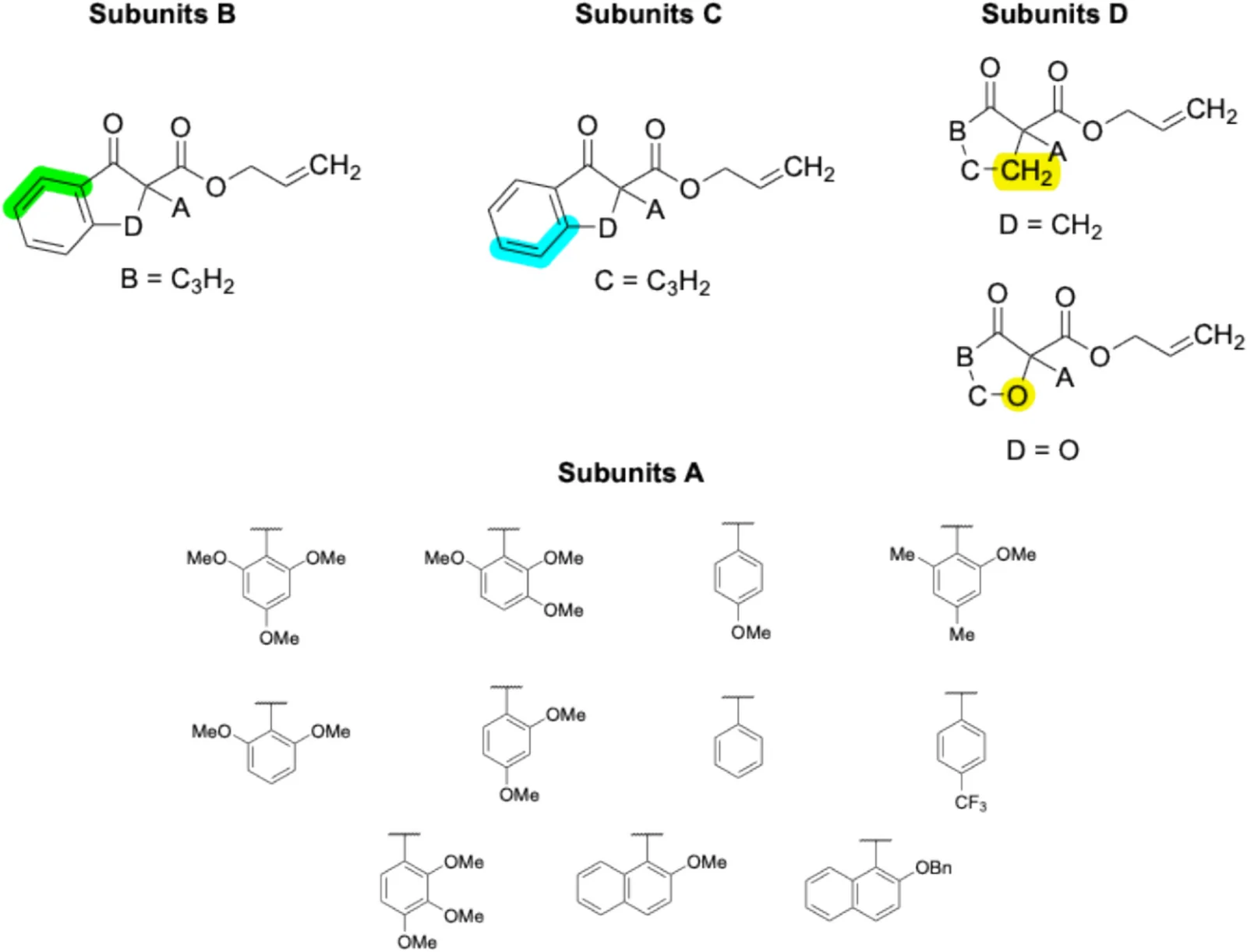
New substrate chemical space present in the *external validation* dataset

All the datapoints from the *benchmarking* datasets have been used to train ML models using the different algorithms and different representations. The *external validation* (test) dataset is different from the *benchmark* (training) dataset only by the nature of the substrates, as all the reactions were performed using catalyst **III** (Fig. 5) and 1,4-dioxane as solvent, both already present in the *benchmark* datasets. The new substrate chemical space to be extrapolated is shown in Fig. 10.

While subunits B and C are already contained within the training data (Fig. 3 and Fig. 4), the different combinations of A-B-C-D in the *external validation* dataset are completely new from those used in the benchmark dataset. This simulates a real-world synthetic chemistry problem of proposed exploration of new chemical space extrapolation using an existing catalyst.

**Fig. 11:**
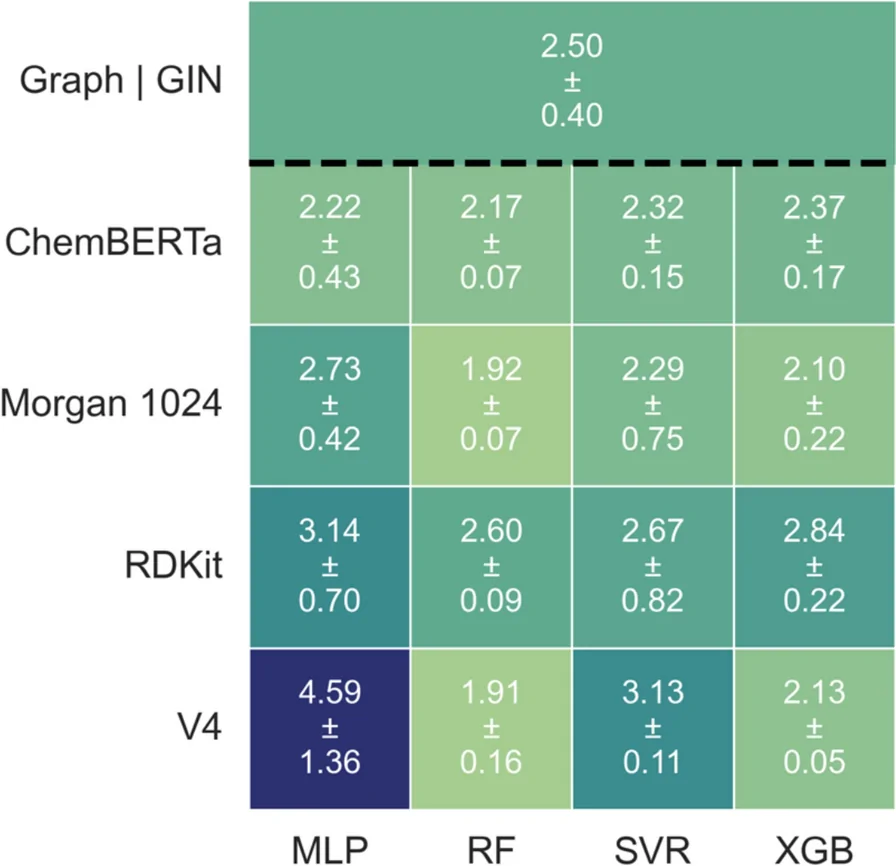
Values of the average ± standard deviation of MAE values in kJ mol^ −1^ for prediction of the enantioselectivity of reactions in the *external validation* dataset from 5 trials using different random seeds for each ML algorithm and representation pair. Darker colours represent worse fit

Each representation-algorithm pair was used to train five different models using different initialisations from different random seeds. Each trained model was then used to predict the stereoselectivity values of the external validation dataset reactions and the MAE and R^2^ for each trial was collected (Table S37 and Figure S8 and Table S38 and Figure S9 for R^2^ values). Figure 11 summarises the results.

The results show that Morgan is the representation that leads to most accurate predictions (average MAE of 2.33 across different ML algorithms, Figure S10), in agreement with the chemical space extrapolation experiment. However, no statistical difference was found between Morgan Fingerprints and ChemBERTa (p-value = 0.86, Table S39), suggesting the latter performs as well in chemical extrapolation tasks. This result is rather interesting, as the simulation of chemical space extrapolation showed that ChemBERTa would not be as accurate as Morgan Fingerprints. A possible reason for this is that as the training set is bigger for this test than the simulated experiments, allowing better ChemBERTa-target property ML model mapping. Furthermore, graph representations are found to be statistically undistinguishable from all other representations (Table S40), reaffirming that when enough data is available, it can attain comparable performance to traditional approaches in extrapolative tasks. The V4 bespoke descriptor developed herein leads, in general, to the worst results along with RDKit. A possible explanation for this is that V4 is more heavily affected by the changes of the A-B-C-D units as these are explicitly encoded, thus, overfitting to the training. However, this result is due to the overfitting of MLP, as evidenced by high MAE values (Fig. 11), while using RF and XGB led to similar results to Morgan fingerprints, suggesting high predictive power when combined with appropriate algorithms.

In the case of ML algorithms, RF is found to be the best performing ML algorithm (Figure S10, Table S41), also in agreement with the scaffold splitting experiments. GIN was found to be only statistically different from MLP (Table S42), similarly to the simulated extrapolative task experiment. The results suggest that the scaffold-based splitting strategy is able to provide more realistic trends of how ML models will perform on real-world extrapolative tasks for substrate discovery than simple random splitting.

**Fig. 12:**
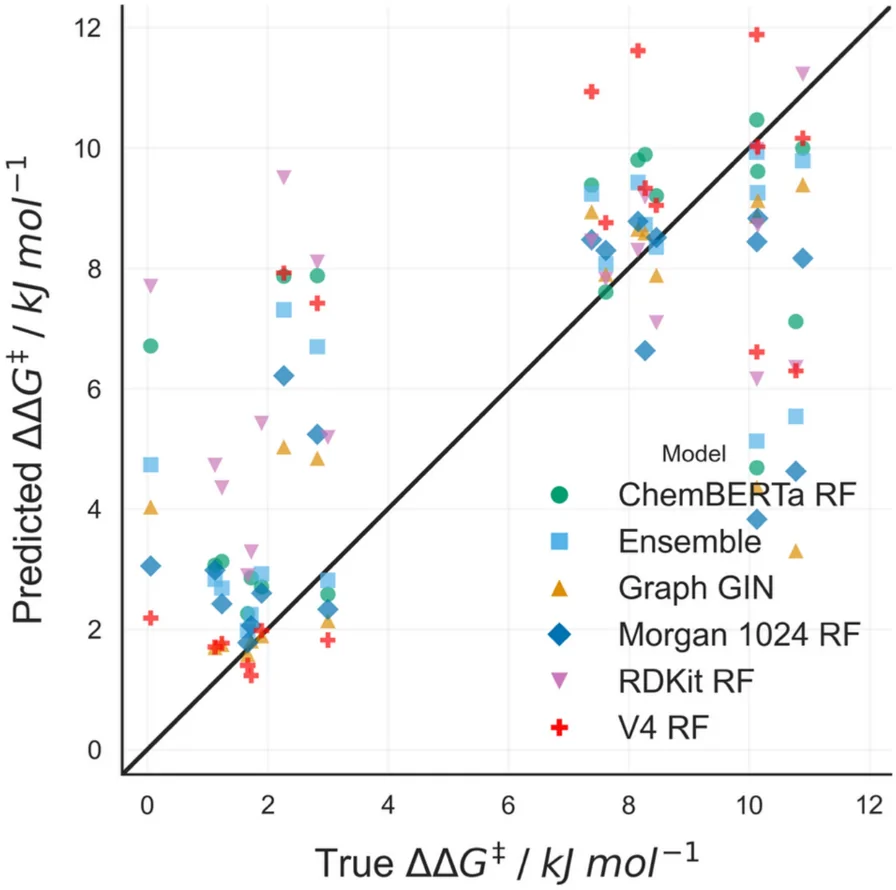
Parity plot of the predicted and real ΔΔG^‡^ in kJ mol^ −1^ for the best model attained with each analysed representation and the ensemble model from those models

To analyse correlations, the predictions from the best models for each representation (based on the mean MAE on Fig. 11) were averaged over the 5 random seed trials (effectively creating an ensemble of models from 5 random initialisations). Furthermore, predictions from all the models (25 models) were averaged to create an ensemble of models with different representations. The results are shown in Fig. 12, and corresponding metrics of ensemble models in Table 1.

**Table 1:** Metrics of ensemble model predictions on the external validation DAAA dataset

Ensemble model	MAE/kJ mol^ −^1	R^2^
ChemBERTa RF (●)	2.16	0.42
Ensemble all representations (■)	1.86	0.55
Graph GIN (▲)	1.64	0.56
Morgan RF (◆)	1.92	0.54
RDKit RF (▼)	2.60	0.22
V4 RF (✚)	1.89	0.57

From Fig. 12 and Table 1, it is noticeable that RDKit led to the worst performing models, in agreement with the observations made in other experiments, while other models perform very similarly. Very interestingly, while in general the ensemble approach lowered the MAE compared to the mean MAE from the 5 random seeds in Fig. 11, the GIN model showed the biggest gain (2.50 kJ mol^ −1^ for the mean approach compared to 1.64 kJ mol^ −1^ of the ensemble approach). This means that graph modelling can greatly benefit from ensemble predictors based on GNNs initialised from different random seeds. By making use of this approach, the ensemble of GNNs is found to be the best performing model. The bespoke V4 representation is found to be among the top performing models, which is explained by the descriptors generated from domain expertise in this transformation. Morgan Fingerprints are also found to be very competitive, which has been demonstrated across experiments. In the case of ChemBERTa, while representation grouping showed to be statistically similar to Morgan Fingerprints, its RF model is relatively worse than the other representations using the RF algorithm. In the case of the ensemble built from all different representations, its performance is comparable to the best performing individual models, suggesting asymmetric catalysis could really benefit from ensemble modelling using multiple representations.

Outliers on Fig. 12 were analysed further to understand what the models were failing to predict. It was found that five substrates with different relative methoxy positioning on the aryl unit of the substrate were being predicted with errors > 2 kJ mol^ −1^ by all the models (see Figure S11). A possible reason for the error of prediction is that substrates with similar aryl groups in the training data showed different selectivity values than those on these new molecules, thus, making the models fail the predictions. This suggests that these transformations suffer of electronic and/or steric effects that don’t occur on the training data.

While the exact order of performance of each representation-algorithm from the simulated extrapolation of chemical space experiment differs from the real-world studies, the general trends are kept. This indicates that the extrapolative simulated task was more useful than random splitting in estimating the performance of representation-algorithm pairs in a real-world extrapolation tasks. This result demonstrates the importance of more rigorous splitting strategies for model evaluation than random train/test splits.

### Explainability of the trained models

**Fig. 13:**
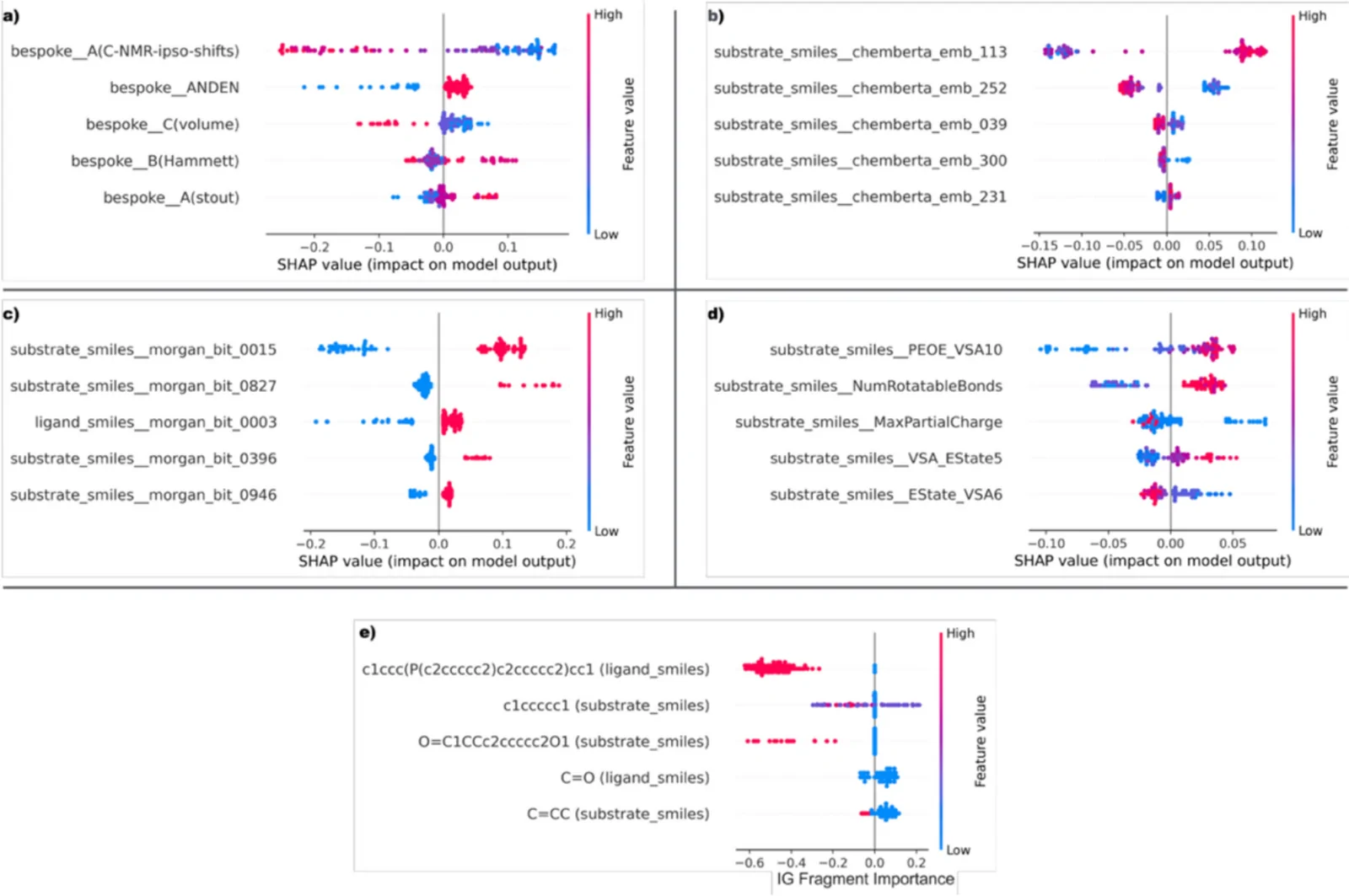
SHAP beeswarm plots for the models **a** V4-MLP, **b** CircuS-SVR, **c** Morgan-SVR and **d** RDKit-SVR. **e** Shows GNN fragment-based explanations using integrated gradients

Each of the best models trained using the *benchmark* dataset (shown in Fig. 12) were explained using SHAP as implemented in Python [86], and in the case of GIN, integrated gradients was used. The SHAP explanations of the models trained using the reproducibility seed 0 were analysed further. Only the top five most important features were analysed, shown in Fig. 13.

In the case of the bespoke V4 representation, the most important feature to deliver predictions is the [13] C NMR shift (of the aryl *ipso* carbon), which confirms the hypothesis that this descriptor is important within the V4 prediction using the bespoke family assessment. The ANDEN subunit was found to greatly affect the outcome, which can be explained as the catalyst **III** showed better levels of stereoselectivity. Interestingly, the volume of the unit C is found to be important as well, presumably due to steric effects. Furthermore, the Hammett parameter of B is found to be influential, which makes sense as this unit is neighbour of the carbon where the addition is made, thus, electronic effects of this unit may lead to stabilised transition states that impact the stereoselectivity. Lastly, steric descriptor of A was found to be important, that also agrees with the experimental observables that show that this substituent plays a major role on the enantioselectivity of the reaction.

In the case of ChemBERTa, clear trends are visible on the SHAP plot (Fig. 13b). Unfortunately, it is not straightforward to correlate what each feature means in terms of chemical structures. While some approaches have proposed the use of visualisation of Queries and Keys matrices to understand how tokens interplay for the transformer to create an embedding and/or prediction, this approach does not lead to chemical fragments that are relevant from a synthetic chemistry point of view. While the ChemBERTa model clearly encodes substrate information that is important for the prediction of enantioselectivities, its trade-off is the lack of interpretation that might complement chemical intuition for onward optimisation of new asymmetric catalytic reactions.

**Fig. 14:**
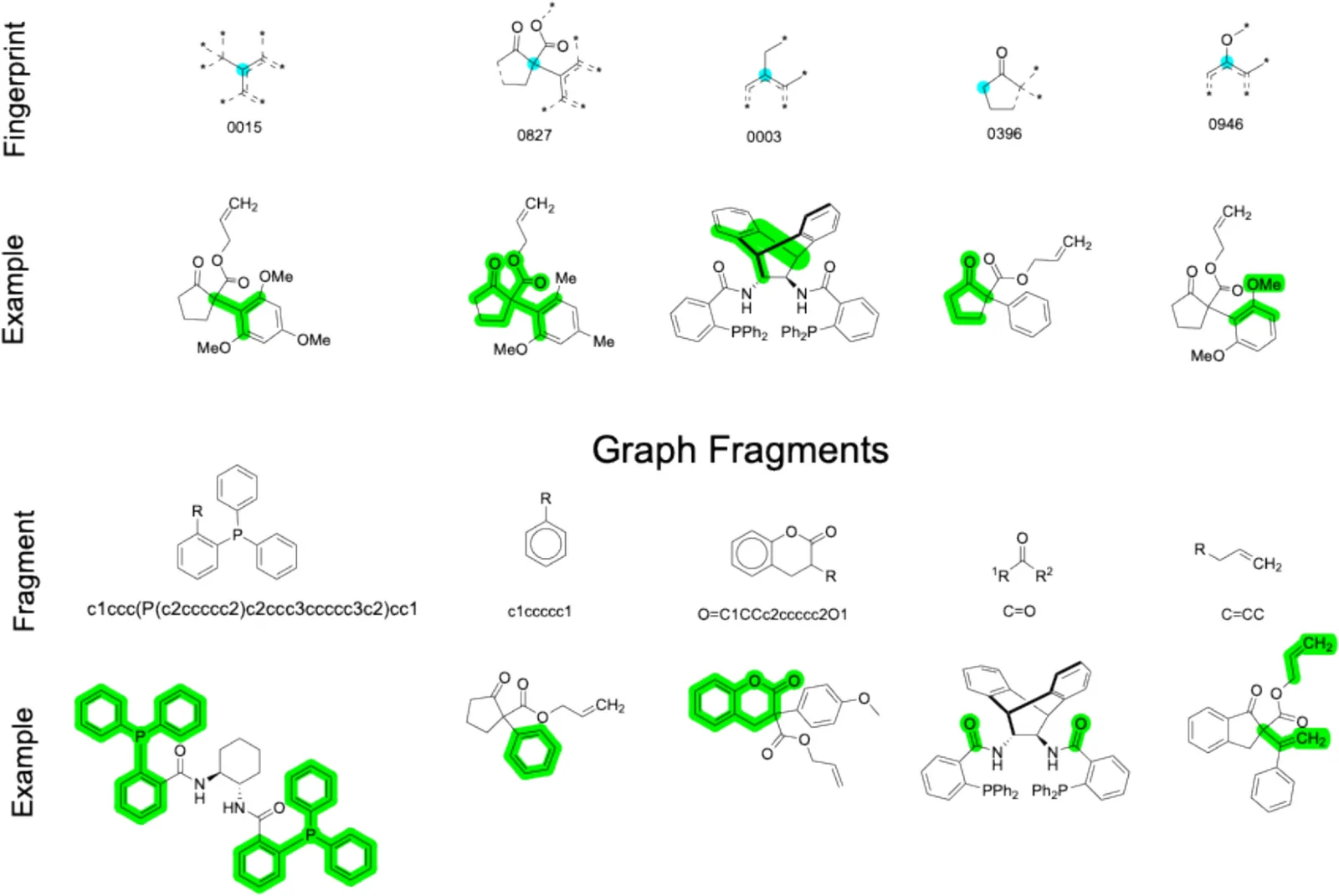
Top 5 most important fragments found by using SHAP on the Morgan-RF model and using Integrated Gradients on the Graph-GIN model. The fingerprint substructures highlight the central atom used for fingerprinting, while the example structures show highlighted the fingerprint in a real molecule example from within the dataset

In the case of Morgan fingerprints (Fig. 13c), only one ligand descriptor was found to be among the 5 most important features. This fingerprint (0003, Fig. 14) related to the ANDEN catalyst structure, similarly to the case the bespoke descriptors. This suggests that the model uses this feature to differentiate the ANDEN subunit of catalyst **III** from the rest. In the case of fingerprint 0015 (Fig. 14), this is directly related to the subunit A, helping differentiate between the subunit A “*m*” (Fig. 4) and the rest. In the case of 0396 and 0827 (Fig. 14), it is clear that this makes the model differentiate between different B-C-D combinations in the substrate. Lastly, fingerprint 0946 is related to oxygens in the *ortho* position of the subunit A. This suggests that oxygens there in the aryl subunit have a major effect on this reaction’s enantioselectivity.

In the case of RDKit descriptors, most of the descriptors do not have direct human intuitive information. From the knowledge of how these descriptors impact the selectivity outcomes further reaction improvements cannot be easily extracted. The only descriptor that is related to a chemically meaningful variable is the number of rotatable bonds in the substrate. However, this feature is not useful in understanding the reaction mechanism or in rationally improving its enantioselectivity.

Lastly, in the case of GNNs, two fragments related to the ligand were found important. The first one, is related to the triphenylphosphine unit within catalysts **I** and **III** (Fig. 5), showing that the model found that, in general, this leads to greater selectivity compared to the diphenyl-naphthyl-phosphine from catalyst **II**. Also, the carbonyl group (C = O) within the ligand is found to be important. This is a rather non-informative result, as all the catalysts have a carbonyl. This suggests that the GNN is likely collecting information about the atoms around this fragment. This hypothesis arises as the GNNs trained make two message passing phases, thus, two bonds away from the carbonyl will lead to information that helps distinguish between catalysts **I**, **II** and **III**. Furthermore, in Trost-type ligands it is recognised that the amide units are key elements in stereocontrol through non-covalent interactions and in defining the chiral ligand pocket. Lastly, substrate related fragments are also found to be important. The phenyl unit was found to be important, showing that the model learns the effect of different aryl units on the ‘a’ position of the substrate. The ‘O = C1CCc2ccccc2O1’ fragment of the substrate is usually correlated with lower selectivity outcome, showing that the model learnt that substrates with this moiety are challenging for enantioselective transformations. Lastly, the allyl group of the substrate is found to be important. It was found that substrates with the ethenyl bridge between (see Fig. 14 for an example) lead to less enantioselective transformations, making the GNN learn that this fragment correlates to a lower predicted ΔΔG^‡^. While these explanations may make sense from a chemical point of view, these must be taken with caution, as these rely on the definition of fragments used to attain higher level information from node attributes that, on their own, do not lead to chemically relevant interpretations. Thus, these explanations are dependent on the fragmentation approach used.

The examples discussed herein demonstrate how each descriptor impacts both model performance and interpretability. While all the descriptors attained comparable performance on the external validation dataset, not all of them deliver the same information from interpretability pipelines. In the case of the bespoke approach, it was possible to understand the impact of the electronic effect of the subunits A and C, as well as the steric effect of the subunits A and the substituents within the ligand. ChemBERTa, while useful for modelling, it did not provide any information that can aid understanding of the reaction. The fingerprinting approaches demonstrated that the ANDEN subunit of the catalyst is preferred when enhancing selectivity, as are the oxygens in the *ortho* position of the subunit A. From RDKit it was not possible to gain any insights, thus limiting the model to be a black box. Lastly, in the case of the graph representation it was possible to identify regions from the catalyst and substrate that the GNN finds important to make a prediction. These considerations are also important when choosing a representation for modelling of asymmetric reactions.

### Protocol transferability to other reactions

**Fig. 15:**
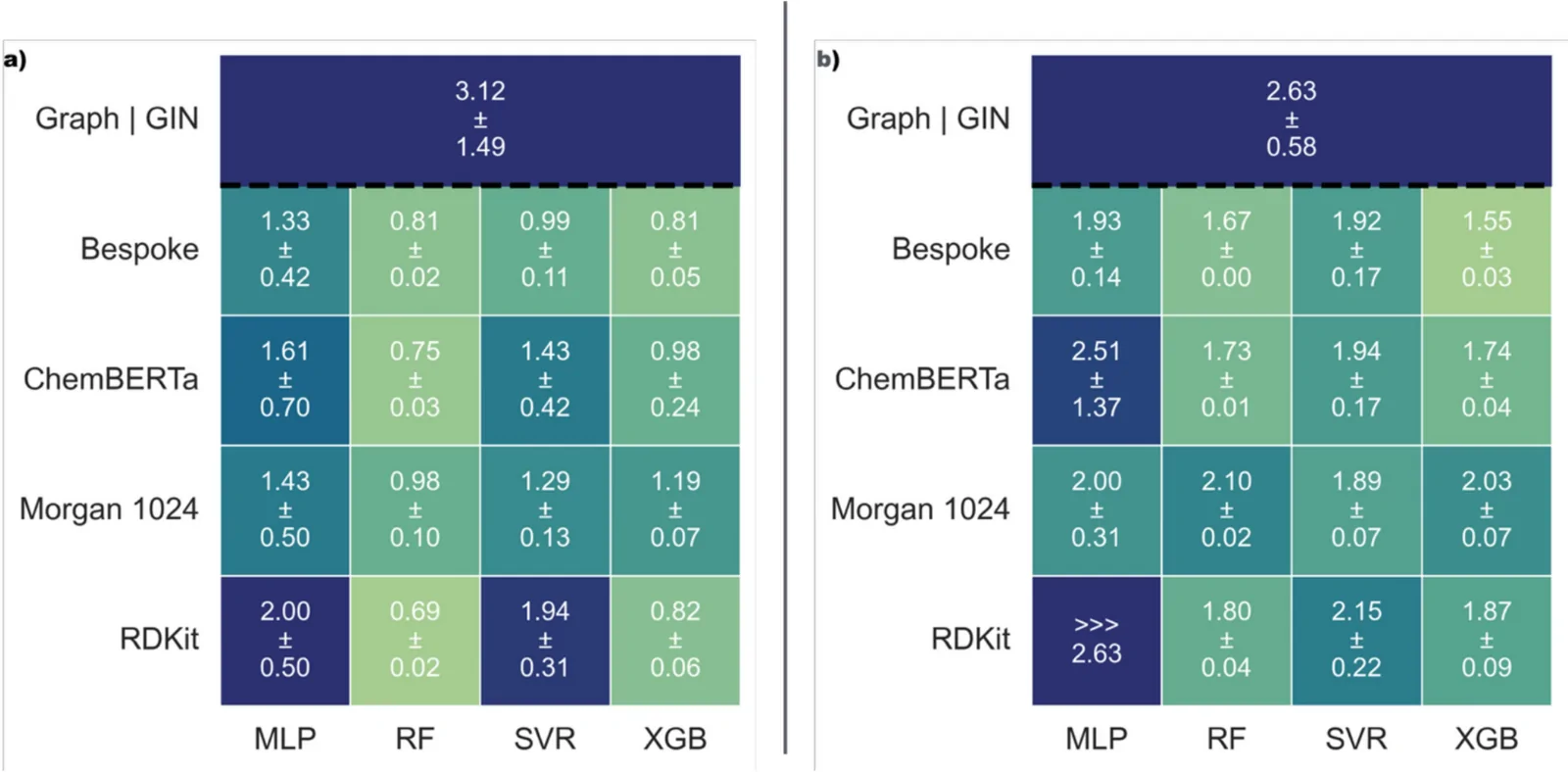
Values of the average ± standard deviation of MAE in kJ mol^−1^ for prediction of the enantioselectivity of reactions in the **a** C-H activation dataset [36] and **b** the asymmetric hydrogenation dataset. [27] from 5 trials using different random seeds for each ML algorithm and representation pair. Darker colours represent worse fit. As RDKit|MLP MAE was significantly larger than the rest of values, this value was ignored for the colour map (see Table S45-S46 for the MAE raw values)

The protocol presented herein is not specific to the DAAA, and it can potentially be applied to any asymmetric reaction. To demonstrate this, we have run the same experiments for the asymmetric hydrogenation from Singh et al. [27], and for the C-H activation dataset from Xu et al*.* [36] As the analyses can be very extensive, in this section only the 8/2 train/test split ratio for scaffold-based splitting results are presented. The results are shown in Fig. 15.

As depicted in Fig. 15, the bespoke strategies are the most consistent representation across different ML algorithms (see Figures S12-S13 and Tables S43-S46). This is an encouraging result, as the biggest contribution in each of the original publications was the developed reaction representation for enantioselectivity prediction. This confirms that this protocol can be applied to a wide range of asymmetric reactions and it will deliver reliable estimations of how good a representation-ML algorithm pair will perform on real-world enantioselectivity optimization developments. Furthermore, the bespoke strategies in both cases made use of DFT descriptors, showing that these can be easily put into the pipeline developed and making possible analyses of their performance compared to simpler strategies.

## Conclusions

A benchmark framework for molecular representations for asymmetric catalysis modelling has been proposed and used for a study case of decarboxylative asymmetric allylic alkylation reaction. The framework aims to test statistically the performance of different representations and ML models in different circumstances to mimic real-world experimentation (especially at the early stages of experimental studies). First, random splitting was tested using different train/test ratios. It was found that Morgan Fingerprints, ChemBERTa, and V4 in general led to the best results. In the case of ML algorithm, RF was found to be statistically better only in high training data domains. It was observed that, with increasing number of datapoints, the representation loses importance and the ML algorithm wins it.

In the case of scaffold-based splitting, the training set size was also varied. It was found that representation plays a critical role in performance for this splitting strategy, regardless of train/test splitting size, demonstrating that representation is critical for chemical extrapolation tasks. Morgan fingerprints were found consistently to be statistically better than the rest of representations, while the relative order of performance of the other approaches depended on the training set size. For this case, RF was also found to deliver more accurate predictions, while MLP in general led to poor models. In the case of GNNs, these only showed comparable performance on the high training data regimes.

An external validation test set was used to evaluate each representation-algorithm pair. It was found that the most accurate representation was, in general, Morgan fingerprints and RF the most accurate ML algorithm, when testing individual models. This agrees with the findings of the chemical space extrapolation task. However, when taking an ensemble approach, it was found that the ensemble of GNNs performed the best among all representations. This result shows that ensembles of GNNs can be useful for asymmetric catalysis modelling.

Using explainability analysis, it was possible to understand variables that affect greatly the performance of the reaction. From the bespoke descriptors, it was found that the ANDEN subunit was key for high selectivity. It was also found that the subunit A steric and electronic effects have major impact on the selectivity. In the case of ChemBERTa, while clear trends were seen on the beeswarm plot, the lack of chemical meaning of the descriptors made impossible to understand what chemical features are important for the model to deliver a prediction. In the case of Morgan Fingerprints, it was seen that most relevant fingerprints related to the ANDEN subunit of the catalyst and to the presence of an oxygen in the *ortho* position of the subunit A, suggesting major electronic effects from this substituent. From the RDKit descriptors, it was not possible to gain any reaction insights due to their complexity. In the case of GNNs, it was found that the fragments with higher attributions were those that are critical for the reaction enantioselectivity, such as the chiral ligand substituents, the substrate allyl group, and the substrate aryl group. This must be taken into consideration if aiming for mechanistic insights from the ML models.

Lastly, it was shown how the pipeline can be easily transferred to other asymmetric reactions to understand how the representation affects the model accuracy. For this, a C-H activation dataset and an asymmetric hydrogenation dataset were tested. It was found that the pipeline finds the bespoke strategies developed in the original studies to be the most consistent for the modelling of the reaction, demonstrating that the methodology can be applied for the identification of robust representations in the asymmetric catalysis context for a wider range of reactions.

## Supplementary Information

Supplementary Material 1.

## Data Availability

All the code necessary to replicate these experiments has been publicly available on GitHub: https://github.com/EdAguilarB/AsymBench

## Abbreviations

DAAA: Decarboxylative asymmetric allylic alkylation
ML: Machine learning
MLP: Multilayer perceptron
RF: Random forest
SVR: Support vector regressor
