# Supplementary material for: Benchmarking molecular representations and machine learning algorithms for asymmetric catalysis: a palladium-catalysed decarboxylative asymmetric allylic alkylation case study
Source: J Cheminform. 2026 Jun 12;18:120. doi: 10.1186/s13321-026-01236-z (PMC13495154; doi:10.1186/s13321-026-01236-z)
Supplement: Supplementary file 1 [file 13321_2026_1236_MOESM1_ESM.docx]

Benchmarking Molecular Representations and Machine Learning Algorithms for Asymmetric Catalysis: A Palladium-catalysed Decarboxylative Asymmetric Allylic Allylation Case Study

Eduardo Aguilar-Bejarano, ^1,2,3 ‡^ Declan Galvin,^4 ‡^ David M. Rogers,^1^ Ender Özcan,^3^

Simon Woodward,^1,2*^ Patrick J. Guiry,^4*^ Grazziela Figueredo^5*^

^1^School of Chemistry, University of Nottingham, University Park, Nottingham, NG7 2RD, United Kingdom.

^2^GSK Carbon Neutral Laboratories for Sustainable Chemistry, University of Nottingham, Jubilee Campus, Triumph Road, Nottingham, NG7 2TU, United Kingdom.

^3^School of Computer Science, University of Nottingham, Jubilee
Campus, Nottingham, NG8 1BB, United Kingdom

^4^School of Chemistry, Centre for Synthesis and Chemical Biology, University College Dublin, Belfield, D04 N2E5, Dublin, Ireland

^5^School of Medicine, University of Nottingham, University Park, Nottingham, NG7 2RD, Nottinghamshire, United Kingdom.

‡ These authors contributed equally.

*Corresponding author: E-mail: [simon.woodward@nottingham.ac.uk](mailto:simon.woodward@nottingham.ac.uk), [patrick.guiry@ucd.ie](mailto:patrick.guiry@ucd.ie), [g.figueredo@nottingham.ac.uk](mailto:g.figueredo@nottingham.ac.uk)

Supporting Information

Table of Contents

[1.	Molecular representations	3](#_Toc228962790)

[1.1	RDKit physicochemical descriptors	3](#_Toc228962791)

[1.2	Molecular fingerprints	3](#_Toc228962792)

[1.2.1	Morgan fingerprints	3](#_Toc228962793)

[1.2.2	CircuS fingerprints	3](#_Toc228962794)

[1.3	Graph representation	4](#_Toc228962795)

[1.4	Embedding-based representation	4](#_Toc228962796)

[1.4.1	ChemBERTa	5](#_Toc228962797)

[1.4.2	MolT5	5](#_Toc228962798)

[1.4.3	UniMol	5](#_Toc228962799)

[1.5	Pre-computed bespoke descriptors	6](#_Toc228962800)

[1.5.1	Calculated Hammett Parameters for fragment A (Figure 3 main paper)	6](#_Toc228962801)

[1.5.2	Asymmetric hydrogenation bespoke descriptors	7](#_Toc228962802)

[1.5.3	C-H activation bespoke descriptors	8](#_Toc228962803)

[2.	ΔΔG^‡^ target variable calculation	8](#_Toc228962804)

[2.1	ΔΔG^‡^ values of benchmarking datasets	8](#_Toc228962805)

[3.	HCat-GNet architecture and training	9](#_Toc228962806)

[3.1	Message-passing architectures	9](#_Toc228962807)

[3.1.1	Graph Convolutional Network (GCN)	9](#_Toc228962808)

[3.1.2	Graph Attention Network (GAT)	9](#_Toc228962809)

[3.1.3	Graph Isomorphism Network (GIN)	10](#_Toc228962810)

[3.2	Graph pooling	10](#_Toc228962811)

[3.3	Readout Network	11](#_Toc228962812)

[3.4	Training procedure	11](#_Toc228962813)

[4.	Statistical analysis	11](#_Toc228962814)

[5.	Explainability	12](#_Toc228962815)

[5.1	SHAP Attributions for Tabular Models	12](#_Toc228962816)

[5.2	Integrated Gradients for GNN Models	13](#_Toc228962817)

[5.2.1	Fragment-level aggregation	13](#_Toc228962818)

[6.	Random splitting raw data and supporting figures	13](#_Toc228962819)

[6.1	Train/test 4/6 ratio splitting	13](#_Toc228962820)

[6.2	Train/test 6/4 ratio splitting	15](#_Toc228962821)

[6.3	Train/test 8/2 ratio splitting	18](#_Toc228962822)

[6.4	Random splitting complementary figures	20](#_Toc228962823)

[7.	Scaffold-based splitting raw data and supporting figures	22](#_Toc228962824)

[7.1	Train/test 4/6 ratio splitting	22](#_Toc228962825)

[7.2	Train/test 6/4 ratio splitting	25](#_Toc228962826)

[7.3	Train/test 8/2 ratio splitting	27](#_Toc228962827)

[7.4	Scaffold-based splitting complementary figures	29](#_Toc228962828)

[8.	Target variable-based splitting raw data	32](#_Toc228962829)

[9.	External validation dataset raw data and complimentary figures	33](#_Toc228962830)

[9.1	Raw data external validation test set	33](#_Toc228962831)

[9.2	Complementary figures	35](#_Toc228962832)

[10.	Other asymmetric reactions results	37](#_Toc228962833)

[10.1	C-H activation	37](#_Toc228962834)

[10.1.1	Raw data C-H activation	37](#_Toc228962835)

[10.1.2	Complementary figures	39](#_Toc228962836)

[10.2	Asymmetric hydrogenation	39](#_Toc228962837)

[10.2.1	Raw data asymmetric hydrogenation	39](#_Toc228962838)

[10.2.2	Complementary figures	40](#_Toc228962839)

[11.	References	41](#_Toc228962840)

# 1. Molecular representations

All representations are computed per reaction component: each SMILES column (substrate, ligand, solvent, etc.) is featurised independently, and the resulting vectors are concatenated to form a single fixed-length reaction-level descriptor. The sections below describe each representation in detail.

## 1.1 RDKit physicochemical descriptors

Physicochemical descriptors were computed using RDKit (version 2025.9).^1^ For each molecule, the full set of two-dimensional descriptors available in rdkit.Chem. Descriptors were calculated, yielding a vector of approximately 200 properties per molecule. These descriptors capture a broad range of molecular characteristics, including topological indices (e.g. Wiener index, Zagreb indices), electronic properties (e.g. TPSA, Crippen log P and molar refractivity), fragment counts (e.g. number of hydrogen bond donors and acceptors, number of rotatable bonds), and graph-theoretic quantities (e.g. χ connectivity indices, κ shape indices). The complete descriptor list is enumerated at featuriser initialisation and applied uniformly to all molecules; no descriptor selection or filtering is performed at this stage. For each molecule in a reaction, descriptors are computed from its SMILES string, and the per-molecule vectors are concatenated across all reaction components to produce the reaction-level feature vector.

## 1.2 Molecular fingerprints

### 1.2.1 Morgan fingerprints

Reaction components were encoded as Morgan circular fingerprints,^2^ computed using the rdFingerprintGenerator.GetMorganGenerator interface in RDKit. The algorithm operates by iteratively aggregating the atomic environment of each heavy atom up to a user-specified radius *r*, hashing the resulting circular substructure identifiers, and folding them into a fixed-length bit vector of size *n*. A bit is set to 1 if the corresponding hashed substructure is present in the molecule. In this work, a radius of *r* = 2 bonds (equivalent to ECFP4) and a vector length of *n* = 2048 bits and *n* = 1024 were used as the configuration. Because bits encode the presence or absence of structural motifs rather than their count, the representation is binary. Each reaction component is fingerprinted independently, and the per-molecule bit vectors are concatenated to form the reaction-level descriptor.

### 1.2.2 CircuS fingerprints

CircuS (**Circu**lar **S**ubstructures) fingerprints^3^ were computed using the ChythonCircus class from the doptools library.^4^ Unlike Morgan fingerprints, which project substructures into a fixed-length hashed bit vector, CircuS defines its feature space empirically: the descriptor columns are determined solely by the circular substructures observed in the training set. Each substructure of bond radius between lower (default 0) and upper (default 2) is extracted from the training molecules and forms one column of the feature matrix, with the corresponding entry recording its frequency of occurrence in a given molecule. Molecules in the test set are featurised based on the chemical space seen on the training set, thus, new fragments in the test set are not featurised.

## 1.3 Graph representation

Each reaction is encoded as a single disconnected molecular graph, constructed by merging the individual molecular graphs of all reaction components (substrate, ligand, solvent) into one PyTorch Geometric Data object.^5^ Molecules are parsed from SMILES strings using RDKit. Each molecule is converted to a directed graph in which nodes correspond to heavy atoms (or all atoms if hydrogens are included) and edges correspond to bonds, with both directions represented for each bond. The component molecular graphs are concatenated by offsetting the node indices of each successive component by the cumulative node count of all preceding components, preserving intra-molecular connectivity while introducing no inter-molecular edges.

**Atom (node) features** comprise a 33-dimensional vector constructed by concatenating:

| **Feature** | **Encoding** | **Dimension** |
| --- | --- | --- |
| Element | one-hot over {H, B, C, N, O, F, Si, P, S, Cl, Br, I} | 12 |
| Degree | one-hot over {0, 1, 2, 3, 4, 5, 6} | 7 |
| Hybridisation | one-hot over {SP, SP², SP³, SP³D, SP³D²} | 5 |
| Formal charge | one-hot over {−2, −1, 0, +1, +2} | 5 |
| Chirality | one-hot over {tetrahedral CW, tetrahedral CCW} | 2 |
| Is aromatic | binary | 1 |
| Is in ring | binary | 1 |

Unrecognised values (e.g. an element outside the allowed set, or unspecified chirality) produce an all-zeros entry for that sub-vector. Stereochemistry is perceived via Chem.AssignStereochemistry before featurisation to ensure chiral tags and bond-stereo flags are correctly populated.

**Bond (edge) features** comprise an 8-dimensional vector constructed by concatenating:

| **Feature** | **Encoding** | **Dimension** |
| --- | --- | --- |
| Bond type | one-hot over {single, double, triple, aromatic} | 4 |
| Bond stereo | one-hot over {Z, E} | 2 |
| Is in ring | binary | 1 |
| Is conjugated | binary | 1 |

The final reaction graph has shape **x** ∈ ℝ^{N × 33} for node features and **edge_attr** ∈ ℝ^2E × 8^ for edge features, where *N* is the total number of atoms across all reaction components and *E* is the total number of bonds.

## 1.4 Embedding-based representation

Three transformer-based featurisation methods are implemented, all operating on SMILES strings as input. For reactions with multiple components, each molecule is embedded independently, and the resulting embedding vectors are concatenated across all SMILES columns to produce a single reaction-level representation. Input SMILES are canonicalised via RDKit prior to tokenisation in all cases, ensuring a consistent and unambiguous string representation.

### 1.4.1 ChemBERTa

ChemBERTa^6^ is a family of RoBERTa-based^7^ language models pre-trained on large collections of SMILES strings from PubChem using masked-language modelling (MLM) and/or chemical-property prediction objectives. The model treats a SMILES string as a sequence of tokens and learns contextualised representations through self-attention, capturing implicit chemical knowledge encoded in the SMILES syntax without requiring any hand-crafted descriptors.

Molecular embeddings are obtained by passing a canonicalised SMILES string through the model's tokeniser and then through the full encoder stack. The tokeniser applies a pre-trained byte-pair encoding (BPE) vocabulary and truncates sequences to a maximum of 512 tokens. The encoder produces a tensor of last-layer hidden states of shape [1, *L*, *H*], where *L* is the sequence length and *H* is the model's hidden size. The molecular embedding is computed by pooling across the sequence dimension. Two pooling strategies are supported: **mean pooling**, in which the hidden states of all non-padding tokens are averaged (masked by the attention mask to exclude padding positions); and **CLS pooling**, in which the hidden state of the first (classification) token is taken directly. Mean pooling was used in this work. The resulting embedding is a vector of dimension *H*, transferred to CPU as a NumPy array.

The model is loaded via the HuggingFace transformers library using the AutoModel and AutoTokenizer interfaces. The default checkpoint used was DeepChem/ChemBERTa-77M-MLM, a 77-million-parameter model pre-trained on approximately 77 million molecules using a masked-language modelling objective, yielding embedding vectors of dimension 600.^6^

### 1.4.2 MolT5

MolT5^8^ is a T5-based^9^ encoder-decoder model pre-trained on large unlabelled SMILES datasets using a text-denoising objective adapted to molecular strings, with the goal of learning rich sequence-level representations of molecules for downstream property prediction and molecule captioning tasks.

For featurisation, only the encoder stack of MolT5 is used; the decoder is discarded. The encoder is accessed via the HuggingFace T5EncoderModel interface, which exposes the encoder hidden states without performing any autoregressive decoding. A canonicalised SMILES string is tokenised with the corresponding T5-based tokeniser (maximum length 512 tokens) and passed through the encoder to produce a last-layer hidden state tensor of shape [1, *L*, *D*], where *D* is the encoder model dimension d_model. The same pooling strategies as for ChemBERTa are supported (mean or CLS); mean pooling was used for this work. The default checkpoint used was laituan245/molt5-base, the base-scale variant of the MolT5 family, yielding embedding vectors of dimension 768.

### 1.4.3 UniMol

UniMol^10^ is a universal molecular pre-training framework built on a transformer architecture that operates on three-dimensional atomic representations rather than one-dimensional SMILES strings. The model is pre-trained on approximately 209 million molecular conformers from PubChem using masked-atom prediction and 3D coordinate denoising objectives, enabling it to learn spatially aware molecular representations that capture geometric and conformational information inaccessible to sequence-based models.

Embeddings are computed using the UniMolRepr class from the unimol_tools library, which internally generates low-energy 3D conformers from each input SMILES string (using RDKit's distance-geometry and force-field optimisation pipeline), encodes the atomic coordinates and chemical identities through the UniMol transformer encoder, and returns a fixed-length molecular-level representation obtained by pooling over the per-atom encoder outputs. Only the molecule-level embedding is used; per-atom representations are not requested.

Two model versions are supported: **UniMol v1**, a 48-layer transformer pre-trained on conformer ensembles, and **UniMol v2**, available in multiple parameter scales (84M, 164M, 310M, 570M, and 1.1B parameters). Hydrogen atoms are retained by default (remove_hs=False), as the 3D pre-training objective was designed to include explicit hydrogen geometries. For this work, **UniMol v1** was used, and explicit hydrogen atoms were used.

## 1.5 Pre-computed bespoke descriptors

For reactions where domain-specific or externally computed descriptors are available (e.g. DFT-derived steric and electronic parameters, experimental solvent properties), AsymBench supports loading pre-computed feature tables from CSV or Parquet files. Each file must contain a unique identifier column that matches the index of the main reaction dataset; all remaining columns are treated as numerical features. At initialisation, the features file is loaded, the identifier column is set as the index, and — if an explicit list of feature_columns is provided — only those columns are retained. When no explicit column list is given, all columns remaining after the index is removed are used as features, allowing the full contents of the file to be incorporated without manual enumeration.

Feature look-up at transform time is performed by reindexing the pre-loaded feature table against the indices of the current data partition (training set or test set), ensuring that rows are aligned to the correct reactions regardless of their order in the DataFrame. In strict mode (default), a KeyError is raised if any reaction index is absent from the features table, preventing silent feature misalignment. In non-strict mode, missing rows are filled with zeros. An optional string prefix may be prepended to all feature column names to avoid naming collisions when multiple bespoke feature tables are used in the same benchmark.

Because bespoke features do not require any molecular computation, they are classified as fit-free representations: the complete feature matrix is computed once on the full dataset at the start of a benchmark run and persisted to disk. Subsequent experiments retrieve features by O(1) index look-up into the in-memory DataFrame, incurring no additional file I/O or computation during the training loop.

### 1.5.1 Calculated Hammett Parameters for fragment A (Figure 3 main paper)

For the reaction representation **V1,** the DFT method developed by Ziegler^11^ was employed, where theoretical Hammett values for the gas phase were obtained for substituted phenylacetic acids that we desired. Substitution patterns with known solution phase Hammett values were then graphed comparing their theoretical gas phase values against their known solution phase values. The resulting correlation was then used to estimate the remaining substituted phenylacetic acids theoretical solution phase Hammett values.

Density functional theory (DFT) computations were performed using Q-Chem 5.3. The B3LYP hybrid exchange-correlation functional was employed with the 6-311++G(d,p) basis set. Geometry optimisation followed by analytic harmonic vibrational frequency calculation was carried out for all species for thermochemical corrections (enthalpy and entropy) at 298.15 K and 1 atm.

More simplistic, but computationally less demanding, approaches were also explored. Here known σ_para_ and σ_meta_ values are combined to approximate the overall electronics of the aryl unit. In approach **V2**, we assumed an σ_ortho_ parameter that would have electronic effect to the equivalent σ_para_ substituent value. The Hammett parameters were then simply summed, to give an approximate overall electronic factor (**Figure S1, left**). Typically, pure σ_ortho_ electronic Hammett parameters are not available, as the effects of steric and electronic cannot be separated experimentally. A second approximation was also evaluated (**V3**), whereby substituents on the aryl ring were assumed to be their equivalent to an equivalent σ_para_ Hammett value and these were then averaged over the possible ring substituent locations (**Figure S1, right**).

**Figure S1.** Strategies explored herein to generate electronic descriptors in the different A substituents within the substrate.

For comparison, a final ­­^13^C NMR spectroscopic descriptor was also included as **V4**. The ^13^C NMR chemical shifts of the *ipso*-carbon of the aryl reflects the electronics at that position. More electron deficient aryl units have *ipso*-carbon ^13^C NMR chemical shifts more downfield, compared to electron-rich aryl units. Thus, we compiled the chemical shifts (in ppm) of the *ipso*-carbon of the aryl unit of the each of the substrates, taken from the original data source of each reaction, and employed them as a final electronic descriptor.

### 1.5.2 Asymmetric hydrogenation bespoke descriptors

The descriptors from the work of Singh *et al.* were sourced from the original publication and were not recalculated by us.^12^ These consisted on descriptors of the catalyst and substrate were generated from optimised structures at the M06-2X/6-31G** in the condensed phase. The descriptors consisted of structural and electronic variables of the molecules, such as bond lengths, bond angles, dihedral angles, distance between non-bonded atoms, vibrational frequencies, vibrational intensities, chemical shifts, charges and global descriptors (such as HOMO and LUMO energies, dipole moment, polar surface area, etc). For the generation of features, these were generated from common core structures within all catalysts in the dataset. To do this, core structures of the catalysts were canonically numbered, which led to a systematic approach to generate descriptors around certain atoms within the catalyst structure. For complete details, refer to the original publication.^12^

### 1.5.3 C-H activation bespoke descriptors

The descriptors from the work by Xu *et al.* were sourced from the original publication and were not recalculated by us.^13^ To calculate them, Xu *et al.* developed an approach to generate ‘transition state structure-like’ distorted geometries. From these distorted geometries, 405 physical organic features were generated for two different transition states in the reaction. For complete details, refer to the original publication.^13^

# 2. ΔΔG^‡^ target variable calculation

The measured values of selectivity of the different reactions within our two databases were reported in terms of the enantiomeric ratio and enantiomeric excess. To transform this variable into a ΔΔG^‡^ value, we first converted the enantiomeric excess values to enantiomeric ratio using the Equation [1].

$$er=\frac{(ee+100)}{2}\cdot100 [1]$$

The *ee* of the top isomer was obtained. Then, we assumed chemical equilibrium of the transition states leading to the two possible enantiomeric products of the reaction, which led to Equation [2].

$$K=\frac{er_{top}}{100-er_{top}} [2]$$

Lastly, to attain the Gibbs free energy, we took the K values and used Equation [3].

$$\Delta\Delta G^{\ddagger}=-RTln\left( K \right) [3]$$

## 2.1 ΔΔG^‡^ values of benchmarking datasets

**Figure S2.** Distribution of ΔΔG^‡^ values for the **a)** asymmetric hydrogenation database from Singh *et al.*^12^ and **b)** the C-H activation dataset from Xu *et al*.^13^

# 3. HCat-GNet architecture and training

HCat-GNet is a GNN that consists of two phases: message-passing phase and readout. In the message passing phase, the GNN explores the topology of the graph to iteratively update the node features based on the neighbouring nodes. In the case of the readout, the high-level learnt node features are summarised to get graph-level vectorial representations that are then put into a multilayer perceptron that delivers the final prediction. These phases are discussed in depth in the following sections.

## 3.1 Message-passing architectures

All GNNs effectively explore the topology of a graph based on a message passing mechanism. In the case of this study, different algorithms were used: GCN, GAT and GIN. For all cases, the network received the raw node features (size 33) and were expanded to 64 by a first convolution layer. Then, another convolution was applied, effectively allowing each node to see two neighbours away from itself. In the next sections the specific architectures and implementations are discussed.

### 3.1.1 Graph Convolutional Network (GCN)

The GCN architecture follows Kipf & Welling,^14^ implemented via GCNConv from PyTorch Geometric. Each layer computes a symmetrically normalised sum over the local neighbourhood, including a self-loop:

$$\mathbf{h}_{i}^{\left( k \right)}=\mathbf{W}^{\left( k \right)}\sum_{j\in\mathcal{N}(i)\cup\{i\}} \frac{1}{\sqrt{\hat{d}_{i}\hat{d}_{j}}}\text{ }\mathbf{h}_{j}^{\left( k - 1 \right)}$$

where $\hat{d}_{i}=1+\sum_{j\in\mathcal{N(}i)} 1$ is the degree of node $i$ after adding the self-loop, and $\mathbf{W}^{\left( k \right)}$ is a trainable weight matrix. An optional `improved` variant replaces the self-loop weight with 2, which has been shown to improve performance on some benchmarks. Unlike GIN-E and GAT, GCN does not incorporate edge features during message passing — all aggregation is purely topology-based. After each `GCNConv` layer, batch normalisation and the chosen activation function are applied sequentially:

$$\mathbf{h}_{i}^{\left( k \right)}\leftarrow\mathrm{Activation}\left( \mathrm{BatchNorm}\left( \mathbf{h}_{i}^{\left( k \right)} \right) \right)$$

### 3.1.2 Graph Attention Network (GAT)

The GAT architecture uses GATv2Conv^15^ from PyTorch Geometric. Attention coefficients depend only on the source or target node independently, not on their combination. GATv2 fixes this by computing attention *after* concatenating source and target features, making the scoring function strictly more expressive:

$$e_{ij}^{\left( k \right)}=\mathbf{a}^{\top}\mathrm{LeakyReLU}\left( \mathbf{W}\left[ \mathbf{h}_{i}^{\left( k - 1 \right)}\text{ }\parallel\text{ }\mathbf{h}_{j}^{\left( k - 1 \right)}+\mathbf{W}_{e}\text{ }\mathbf{e}_{ij} \right] \right)$$

$$\alpha_{ij}^{\left( k \right)}=\frac{\exp(e_{ij}^{\left( k \right)})}{\sum_{l\in\mathcal{N(}i)} \exp(e_{il}^{\left( k \right)})},\mathbf{h}_{i}^{\left( k \right)}=\sigma\left( \sum_{j\in\mathcal{N(}i)} \alpha_{ij}^{\left( k \right)}\text{ }\mathbf{W}^{\left( k \right)}\mathbf{h}_{j}^{\left( k - 1 \right)} \right)$$

where $\mathbf{W}_{e}$ is a learnable projection of the bond feature vector $\mathbf{e}_{ij}$ into the attention scoring space. Multi-head attention is used, with $H$ independent attention functions computed in parallel. In contrast to the original GAT where head outputs are concatenated (producing an $H$-fold expansion of the embedding dimension), here head outputs are averaged (`concat=False`), keeping the node embedding width fixed at $d_{h}$ regardless of the number of heads. This avoids a dimension mismatch across layers without requiring an additional projection. Dropout is applied both inside the attention coefficient computation (to the $\alpha_{ij}$ weights) and after the full aggregation step. As in GCN, batch normalisation follows each convolutional layer before the activation function.

### 3.1.3 Graph Isomorphism Network (GIN)

We use a Graph Isomorphism Network with Edge features,^16^ implemented via GINConv from PyTorch Geometric. GINE extends the Weisfeiler–Leman graph isomorphism test to incorporate edge attributes by adding the (linearly projected) edge feature to each neighbour's node feature before aggregation:

$$\mathbf{h}_{i}^{\left( k \right)}=\mathrm{MLP}^{\left( k \right)}\text{ }\left( (1+\varepsilon)\text{ }\mathbf{h}_{i}^{\left( k - 1 \right)}+\sum_{j\in\mathcal{N}(i)} \mathrm{ReLU}\left( \mathbf{h}_{j}^{\left( k - 1 \right)} + \mathbf{e}_{ij} \right) \right)$$

where $\varepsilon$ is a fixed scalar (set to 0; train_eps=False). The neighbour aggregation function inside each GINEConv layer is a two-layer MLP:

$$\mathrm{MLP}^{\left( k \right)}:\mathbf{x}\mapsto\mathrm{Linear}(d_{\mathrm{in}},d_{h})\to\mathrm{BatchNorm}(d_{h})\to\mathrm{LeakyReLU}\to\mathrm{Linear}(d_{h},d_{h})$$

with $d_{h}=64$. Two consecutive message-passing layers ($K=2$) are applied, giving each node a receptive field spanning its two-hop neighbourhood. Layer-level batch normalisation is performed inside the MLP of each GINEConv block; no additional normalisation is applied between layers.

## 3.2 Graph pooling

After $2$ rounds of message passing, node embeddings are aggregated into a fixed-length graph embedding using the mean_max pooling strategy. Global mean pooling and global max pooling are computed independently over all nodes belonging to each graph, and the resulting vectors are concatenated:

$$\mathbf{z}=\left[ \frac{1}{\mid\mathcal{V}\mid}\sum_{i\in\mathcal{V}} \mathbf{h}_{i}^{\left( K \right)}\text{ } \parallel\text{ }\max_{i\in\mathcal{V}}\mathbf{h}_{i}^{\left( K \right)} \right]\in\mathbb{R}^{2d_{h}}$$

With $d_{h}=64$, this yields a 128-dimensional graph embedding that captures both the mean activation pattern and the most salient individual-node features.

## 3.3 Readout Network

The graph embedding $\mathbf{z}\in\mathbb{R}^{128}$ is passed through a two-layer readout MLP that produces the scalar property prediction. Each intermediate layer halves the representation dimension and applies batch normalisation followed by LeakyReLU activation; the final layer is a plain linear projection to a scalar output with no activation:

$$128\overset{\mathrm{Linear}+\mathrm{BN}+\mathrm{LeakyReLU}}{\to}64\overset{\mathrm{Linear}}{\to}1$$

Dropout is not applied (dropout=0.0).

## 3.4 Training procedure

The model is trained end-to-end by minimising the mean squared error (MSE) between the scaled predictions and scaled ground-truth labels:

$$\mathcal{L=}\frac{1}{\mid\mathcal{B}\mid}\sum_{i\mathcal{\in B}} \left( {\hat{\tilde{y}}}_{i} - \tilde{y}_{i} \right)^{2}$$

Parameters are updated with the Adam optimiser^17^ at a fixed learning rate of $\eta={10}^{-3}$ with default momentum parameters ($\beta_{1}=0.9$, $\beta_{2}=0.999$). Training runs for a fixed budget of 100 epochs with a mini-batch size of 32 graphs. No learning-rate scheduling or early stopping is applied; the model state at the final epoch is used for evaluation.

# 4. **Statistical analysis**

All statistical comparisons were conducted on mean absolute error (MAE) as the performance metric. The experimental design comprised five machine learning models — Random Forest (RF), Support Vector Regression (SVR), Multi-Layer Perceptron (MLP), eXtreme Gradient Boosting (XGB), and a Graph Neural Network (GNN) — and five molecular representations: Morgan 1024, ChemBERTa, V4, RDKit, and graph-based representations. Each non-graph model was evaluated across all non-graph representations, yielding 20 observations per (model, representation) combination (4 models × 5 random seeds). The GNN was evaluated exclusively with graph representations, yielding 5 observations (5 random seeds). All random seeds were shared across conditions, making observations paired by seed.

Three research questions were addressed, each requiring a tailored statistical approach given the structure of the data.

**Q1: Representation comparison.** The comparison of molecular representations was conducted in two steps due to a structural asymmetry in the design. Graph representations are, by construction, inseparable from the GNN architecture — no non-graph model can be applied to graph inputs and no graph model can be applied to non-graph inputs. This coupling means that any observed difference involving graph representations reflects the joint effect of the representation and the algorithm, i.e. it cannot be attributed to the representation alone. This limitation is inherent to the experimental design and is explicitly acknowledged.

For the comparison among non-graph representations (Morgan 1024, ChemBERTa, V4, and RDKit), the 20 observations per representation (4 models × 5 seeds) provided sufficient power for non-parametric testing. A Friedman test with seed and model jointly defining the blocking structure was applied as an omnibus test, followed by pairwise Wilcoxon signed-rank tests corrected with the Benjamini–Hochberg procedure.

To compare graph representations against non-graph representations, a Linear Mixed Effects Model (LME) was employed, with graph representation set as the reference category. This approach was chosen over Wilcoxon because the graph condition contains only 5 observations — one per random seed — making the Wilcoxon signed-rank test unable to reach conventional significance levels at α = 0.05 due to the discrete nature of its rank-based null distribution (minimum achievable p-value = 0.0625 for n = 5). The LME instead uses a continuous z-distribution for inference, enabling valid hypothesis testing at this sample size. Random seed was modelled as a grouping factor (random intercept), capturing the shared variance introduced by identical train–test splits across conditions. Model coefficients for each non-graph representation directly quantify the difference in MAE relative to the graph representation, controlling for seed-level variability.

**Q2: Algorithm comparison.** By symmetry with Q1, the comparison of non-graph algorithms was conducted using the Friedman test followed by pairwise Wilcoxon signed-rank tests on the 20 observations per algorithm (4 representations × 5 seeds). The GNN was compared against non-graph algorithms using an LME with identical structure to that described above, with GNN set as the reference category. The same caveat regarding the inseparability of the GNN from its representation applies here.

All tests were two-sided. Significance was assessed at α = 0.05 after multiple testing correction.

# 5. Explainability

Both tabular and graph-based models are accompanied by post-hoc feature attribution to identify which molecular features and structural motifs drive yield predictions.

## 5.1 SHAP Attributions for Tabular Models

For models operating on fixed-length feature vectors, attributions are computed using SHAP.^18^ Tree-based models (random forest, XGBoost) use TreeExplainer, which exploits the tree structure to compute exact Shapley values efficiently; all other models (SVR, MLP) use a permutation-based estimator against a background sample drawn from the training set. Global feature importance is reported as the mean absolute SHAP value across the test set and visualised as a beeswarm summary plot.

## 5.2 Integrated Gradients for GNN Models

Graph neural networks are explained using Integrated Gradients (IG)^19^, which attributes the model output to each node feature by integrating the gradient along a straight-line path from an all-zeros baseline to the actual input. This produces a signed attribution matrix over all atoms and node features for each reaction graph: positive values indicate a feature pushes the prediction above the baseline; negative values indicate suppression. Edge attributes and global reaction features are held fixed throughout the interpolation, so that attributions are specific to node-level molecular features.

### 5.2.1 Fragment-level aggregation

Atom-level IG scores are aggregated to chemically interpretable fragment importances. Each molecule is decomposed by BRICS fragmentation^20^ using RDKit, and each fragment is matched back to the parent structure via substructure search to obtain atom indices. The importance of a fragment occurrence is then the sum of all IG attributions over its constituent atoms and features. Because reactions involve multiple components, fragments are tracked by both their SMILES and their source column (e.g. substrate vs. ligand), so that the same substructure appearing in different roles is attributed independently.

To create beeswarm plots, the attributions of all the fragments from all the different molecules in all the different reactions were obtained. Since the graph representation is not structures as a vector representation, in some cases, a given fragment was completely absent in some molecules. In these cases, the attribution of such fragment for such molecule was given a score of 0. Furthermore, the plot feature values were coloured based on the quantity of times that such fragment appeared in a molecule, delivering a visual depiction of how the frequency of a fragment could ultimately affect the GNN prediction.

# 6. Random splitting raw data and supporting figures

## 6.1 Train/test 4/6 ratio splitting

**Table S1.** Raw MAE values of different representation – ML algorithm combinations for random splitting trials and 4/6 train/test ratio for the DAAA benchmark dataset.

| **Random seed** | **0** | **1** | **2** | **3** | **4** | **mean ± std** |
| --- | --- | --- | --- | --- | --- | --- |
| Morgan 1024 RF MAE mean | 1.756 | 1.763 | 1.772 | 1.878 | 1.747 | 1.78 ± 0.05 |
| ChemBertA 77M-MLM RF MAE mean | 2.194 | 2.174 | 1.81 | 2.115 | 1.771 | 2.01 ± 0.21 |
| ChemBertA 77M-MLM XGB MAE mean | 1.963 | 1.841 | 2.487 | 2.073 | 2.056 | 2.08 ± 0.24 |
| Morgan 1024 XGB MAE mean | 2.085 | 2.238 | 2.021 | 1.876 | 2.36 | 2.12 ± 0.19 |
| V4 MLP MAE mean | 2.012 | 2.442 | 2.15 | 2.015 | 2.21 | 2.17 ± 0.18 |
| Morgan 1024 SVR MAE mean | 2.109 | 2.34 | 1.975 | 2.284 | 2.285 | 2.20 ± 0.15 |
| V4 SVR MAE mean | 1.753 | 2.654 | 3.03 | 1.8 | 1.966 | 2.24 ± 0.57 |
| V4 XGB MAE mean | 2.327 | 2.225 | 2.224 | 2.16 | 2.429 | 2.27 ± 0.11 |
| ChemBertA 77M-MLM SVR MAE mean | 2.269 | 2.342 | 2.336 | 2.42 | 2.293 | 2.33 ± 0.06 |
| Morgan 1024 MLP MAE mean | 2.004 | 2.247 | 2.038 | 2.548 | 2.903 | 2.35 ± 0.38 |
| V4 RF MAE mean | 2.546 | 2.575 | 2.724 | 2.715 | 2.71 | 2.65 ± 0.09 |
| RDKit RF MAE mean | 2.471 | 3.129 | 2.513 | 2.956 | 2.639 | 2.74 ± 0.29 |
| RDKit SVR MAE mean | 2.793 | 2.793 | 2.627 | 2.88 | 2.736 | 2.77 ± 0.09 |
| ChemBertA 77M-MLM MLP MAE mean | 2.312 | 3.414 | 2.399 | 2.973 | 2.746 | 2.77 ± 0.45 |
| RDKit MLP MAE mean | 3.019 | 3.18 | 3.301 | 2.504 | 2.654 | 2.93 ± 0.34 |
| RDKit XGB MAE mean | 2.616 | 2.811 | 3.163 | 3.117 | 2.998 | 2.94 ± 0.23 |
| Graph GIN MAE mean | 2.43 | 2.389 | 2.846 | 3.313 | 4.349 | 3.07 ± 0.81 |

**Table S2.** Raw R^2^ values of different representation – ML algorithm combinations for random splitting trials and 4/6 train/test ratio for the DAAA benchmark dataset.

| **Random seed** | **0** | **1** | **2** | **3** | **4** | **mean ± std** |
| --- | --- | --- | --- | --- | --- | --- |
| Morgan 1024 RF R2 mean | 0.737 | 0.736 | 0.739 | 0.707 | 0.743 | 0.73 ± 0.01 |
| ChemBertA 77M-MLM RF R2 mean | 0.586 | 0.586 | 0.72 | 0.608 | 0.746 | 0.65 ± 0.08 |
| Morgan 1024 XGB R2 mean | 0.615 | 0.616 | 0.691 | 0.728 | 0.598 | 0.65 ± 0.06 |
| Morgan 1024 SVR R2 mean | 0.656 | 0.627 | 0.657 | 0.652 | 0.598 | 0.64 ± 0.03 |
| ChemBertA 77M-MLM XGB R2 mean | 0.668 | 0.68 | 0.531 | 0.615 | 0.629 | 0.62 ± 0.06 |
| V4 MLP R2 mean | 0.633 | 0.541 | 0.616 | 0.645 | 0.621 | 0.61 ± 0.04 |
| V4 SVR R2 mean | 0.732 | 0.481 | 0.343 | 0.704 | 0.687 | 0.59 ± 0.17 |
| ChemBertA 77M-MLM SVR R2 mean | 0.585 | 0.558 | 0.55 | 0.57 | 0.572 | 0.57 ± 0.01 |
| V4 XGB R2 mean | 0.534 | 0.553 | 0.587 | 0.578 | 0.529 | 0.56 ± 0.03 |
| Morgan 1024 MLP R2 mean | 0.657 | 0.565 | 0.655 | 0.49 | 0.315 | 0.54 ± 0.14 |
| RDKit SVR R2 mean | 0.4 | 0.406 | 0.511 | 0.391 | 0.418 | 0.43 ± 0.05 |
| ChemBertA 77M-MLM MLP R2 mean | 0.583 | 0.188 | 0.57 | 0.351 | 0.427 | 0.42 ± 0.16 |
| V4 RF R2 mean | 0.437 | 0.422 | 0.372 | 0.371 | 0.375 | 0.40 ± 0.03 |
| RDKit RF R2 mean | 0.423 | 0.223 | 0.44 | 0.312 | 0.409 | 0.36 ± 0.09 |
| RDKit MLP R2 mean | 0.306 | 0.107 | 0.053 | 0.523 | 0.43 | 0.28 ± 0.20 |
| RDKit XGB R2 mean | 0.404 | 0.352 | 0.134 | 0.266 | 0.219 | 0.28 ± 0.11 |
| Graph GIN R2 mean | 0.526 | 0.538 | 0.375 | 0.072 | -0.45 | 0.21 ± 0.42 |

**Table S3.** Wilcoxon statistical tests for comparing non-graph representations MAE value distributions for 4/6 train/test splitting ratio and random splitting for the DAAA benchmark dataset.

| **group a** | **Group b** | **n** | **Wilcoxon stat** | **P value** | **Median diff a minus b** | **P value corrected** | **Reject null** |
| --- | --- | --- | --- | --- | --- | --- | --- |
| Morgan 1024 | RDKit | 20 | 3.0 | 0.000 | -0.700 | 0.000 | True |
| ChemBertA 77M-MLM | RDKit | 20 | 11.0 | 0.000 | -0.665 | 0.000 | True |
| RDKit | V4 | 20 | 14.0 | 0.000 | 0.561 | 0.000 | True |
| ChemBertA 77M-MLM | Morgan 1024 | 20 | 38.0 | 0.011 | 0.179 | 0.016 | True |
| Morgan 1024 | V4 | 20 | 62.0 | 0.114 | -0.199 | 0.137 | False |
| ChemBertA 77M-MLM | V4 | 20 | 94.0 | 0.701 | -0.200 | 0.701 | False |

**Table S4.** Linear mixed effects model results for comparing representations taking graph representation as baseline model for 4/6 train/test splitting ratio and random splitting for the DAAA benchmark dataset.

| **Variable** | **Coef.** | **Std.Err.** | **z** | **P>\|z\|** | **[0.025** | **0.975]** |
| --- | --- | --- | --- | --- | --- | --- |
| Intercept | 3.065 | 0.164 | 18.732 | 0.000 | 2.745 | 3.386 |
| C(representation, Treatment(reference='Graph'))  [T.ChemBertA 77M-MLM] | -0.766 | 0.182 | -4.214 | 0.000 | -1.122 | -0.410 |
| C(representation, Treatment(reference='Graph'))  [T.Morgan 1024] | -0.954 | 0.182 | -5.248 | 0.000 | -1.310 | -0.598 |
| C(representation, Treatment(reference='Graph'))  [T.RDKit] | -0.221 | 0.182 | -1.213 | 0.225 | -0.577 | 0.136 |
| C(representation, Treatment(reference='Graph'))  [T.V4] | -0.732 | 0.182 | -4.028 | 0.000 | -1.088 | -0.376 |
| Group Var | 0.002 | 0.019 |  |  |  |  |

**Table S5.** Wilcoxon statistical tests for comparing non-graph ML algorithms MAE value distributions for 4/6 train/test splitting ratio and random splitting for the DAAA benchmark dataset.

| **group a** | **Group b** | **n** | **Wilcoxon stat** | **P value** | **Median diff a minus b** | **P value corrected** | **Reject null** |
| --- | --- | --- | --- | --- | --- | --- | --- |
| MLP | SVR | 20 | 54.0 | 0.058 | 0.221 | 0.350 | False |
| MLP | RF | 20 | 57.0 | 0.076 | 0.257 | 0.379 | False |
| MLP | XGB | 20 | 65.0 | 0.143 | 0.077 | 0.572 | False |
| RF | SVR | 20 | 70.0 | 0.202 | -0.185 | 0.607 | False |
| RF | XGB | 20 | 90.0 | 0.596 | -0.072 | 1.000 | False |
| SVR | XGB | 20 | 99.0 | 0.841 | 0.003 | 1.000 | False |

**Table S6.** Linear mixed effects model results for comparing ML algorithms taking GIN as baseline model for 4/6 train/test splitting ratio and random splitting for the DAAA benchmark dataset.

| **Variable** | **Coef.** | **Std.Err.** | **z** | **P>\|z\|** | **[0.025** | **0.975]** |
| --- | --- | --- | --- | --- | --- | --- |
| Intercept | 3.065 | 0.199 | 15.386 | 0.000 | 2.675 | 3.456 |
| C(model, Treatment(reference='GIN'))[T.MLP] | -0.512 | 0.223 | -2.298 | 0.022 | -0.949 | -0.075 |
| C(model, Treatment(reference='GIN'))[T.RF] | -0.767 | 0.223 | -3.445 | 0.001 | -1.204 | -0.331 |
| C(model, Treatment(reference='GIN'))[T.SVR] | -0.681 | 0.223 | -3.059 | 0.002 | -1.118 | -0.245 |
| C(model, Treatment(reference='GIN'))[T.XGB] | -0.712 | 0.223 | -3.196 | 0.001 | -1.149 | -0.275 |
| Group Var | 0.000 | 0.024 |  |  |  |  |

## 6.2 Train/test 6/4 ratio splitting

**Table S7.** Raw MAE values of different representation – ML algorithm combinations for random splitting trials and 6/4 train/test ratio for the DAAA benchmark dataset.

| **Random seed** | **0** | **1** | **2** | **3** | **4** | **mean ± std** |
| --- | --- | --- | --- | --- | --- | --- |
| Morgan 1024 XGB MAE mean | 1.527 | 1.757 | 1.553 | 1.663 | 1.608 | 1.62 ± 0.09 |
| Morgan 1024 RF MAE mean | 1.578 | 1.741 | 1.715 | 1.813 | 1.614 | 1.69 ± 0.10 |
| V4 SVR MAE mean | 1.63 | 1.851 | 1.785 | 1.71 | 1.953 | 1.79 ± 0.12 |
| ChemBertA 77M-MLM RF MAE mean | 1.815 | 1.8 | 1.831 | 1.828 | 1.76 | 1.81 ± 0.03 |
| ChemBertA 77M-MLM XGB MAE mean | 1.954 | 1.986 | 1.882 | 2.059 | 2.076 | 1.99 ± 0.08 |
| V4 RF MAE mean | 2.051 | 2.105 | 2.047 | 2.113 | 2.119 | 2.09 ± 0.04 |
| V4 XGB MAE mean | 2.149 | 2.02 | 2.021 | 2.231 | 2.004 | 2.09 ± 0.10 |
| V4 MLP MAE mean | 1.947 | 2.431 | 2.121 | 2.393 | 1.82 | 2.14 ± 0.27 |
| Morgan 1024 SVR MAE mean | 1.976 | 2.31 | 2.51 | 2.144 | 2.093 | 2.21 ± 0.21 |
| ChemBertA 77M-MLM SVR MAE mean | 1.972 | 2.245 | 2.063 | 2.895 | 2.3 | 2.30 ± 0.36 |
| Graph GIN MAE mean | 1.986 | 2.457 | 2.413 | 2.46 | 2.38 | 2.34 ± 0.20 |
| RDKit XGB MAE mean | 2.166 | 2.392 | 2.285 | 2.554 | 2.361 | 2.35 ± 0.14 |
| RDKit RF MAE mean | 2.323 | 2.386 | 2.418 | 2.476 | 2.671 | 2.45 ± 0.13 |
| Morgan 1024 MLP MAE mean | 2.235 | 2.41 | 3.032 | 2.544 | 2.179 | 2.48 ± 0.34 |
| RDKit SVR MAE mean | 2.503 | 2.384 | 2.47 | 2.533 | 2.575 | 2.49 ± 0.07 |
| ChemBertA 77M-MLM MLP MAE mean | 2.617 | 2.328 | 2.458 | 2.608 | 2.914 | 2.58 ± 0.22 |
| RDKit MLP MAE mean | 2.874 | 2.615 | 2.938 | 1.978 | 2.586 | 2.60 ± 0.38 |

**Table S8.** Raw R^2^ values of different representation – ML algorithm combinations for random splitting trials and 6/4 train/test ratio for the DAAA benchmark dataset.

| **Random seed** | **0** | **1** | **2** | **3** | **4** | **mean ± std** |
| --- | --- | --- | --- | --- | --- | --- |
| Morgan 1024 XGB R2 mean | 0.747 | 0.689 | 0.752 | 0.709 | 0.738 | 0.73 ± 0.03 |
| Morgan 1024 RF R2 mean | 0.767 | 0.688 | 0.687 | 0.647 | 0.755 | 0.71 ± 0.05 |
| V4 SVR R2 mean | 0.724 | 0.664 | 0.676 | 0.701 | 0.61 | 0.68 ± 0.04 |
| ChemBertA 77M-MLM RF R2 mean | 0.651 | 0.652 | 0.628 | 0.65 | 0.668 | 0.65 ± 0.01 |
| ChemBertA 77M-MLM XGB R2 mean | 0.624 | 0.588 | 0.628 | 0.584 | 0.549 | 0.59 ± 0.03 |
| Morgan 1024 SVR R2 mean | 0.625 | 0.509 | 0.459 | 0.562 | 0.609 | 0.55 ± 0.07 |
| V4 MLP R2 mean | 0.593 | 0.402 | 0.532 | 0.376 | 0.639 | 0.51 ± 0.12 |
| V4 XGB R2 mean | 0.473 | 0.518 | 0.516 | 0.469 | 0.534 | 0.50 ± 0.03 |
| ChemBertA 77M-MLM SVR R2 mean | 0.586 | 0.483 | 0.573 | 0.259 | 0.46 | 0.47 ± 0.13 |
| V4 RF R2 mean | 0.416 | 0.428 | 0.464 | 0.472 | 0.451 | 0.45 ± 0.02 |
| Graph GIN R2 mean | 0.499 | 0.289 | 0.478 | 0.439 | 0.399 | 0.42 ± 0.08 |
| RDKit SVR R2 mean | 0.415 | 0.45 | 0.444 | 0.409 | 0.384 | 0.42 ± 0.03 |
| RDKit XGB R2 mean | 0.504 | 0.399 | 0.483 | 0.261 | 0.395 | 0.41 ± 0.10 |
| Morgan 1024 MLP R2 mean | 0.504 | 0.439 | 0.049 | 0.44 | 0.53 | 0.39 ± 0.20 |
| RDKit MLP R2 mean | 0.306 | 0.107 | 0.053 | 0.523 | 0.43 | 0.28 ± 0.20 |
| RDKit XGB R2 mean | 0.404 | 0.352 | 0.134 | 0.266 | 0.219 | 0.28 ± 0.11 |
| Graph GIN R2 mean | 0.526 | 0.538 | 0.375 | 0.072 | -0.45 | 0.21 ± 0.42 |

**Table S9.** Wilcoxon statistical tests for comparing non-graph representations MAE value distributions for 6/4 train/test splitting ratio and random splitting for the DAAA benchmark dataset.

| **group a** | **Group b** | **n** | **Wilcoxon stat** | **P value** | **Median diff a minus b** | **P value corrected** | **Reject null** |
| --- | --- | --- | --- | --- | --- | --- | --- |
| RDKit | V4 | 20 | 11.0 | 0.000 | 0.372 | 0.001 | True |
| Morgan 1024 | RDKit | 20 | 13.0 | 0.000 | -0.637 | 0.001 | True |
| ChemBertA 77M-MLM | RDKit | 20 | 33.0 | 0.006 | -0.405 | 0.011 | True |
| ChemBertA 77M-MLM | Morgan 1024 | 20 | 46.0 | 0.027 | 0.176 | 0.040 | True |
| ChemBertA 77M-MLM | V4 | 20 | 75.0 | 0.277 | 0.019 | 0.333 | False |
| Morgan 1024 | V4 | 20 | 91.0 | 0.622 | -0.142 | 0.622 | False |

**Table S10.** Linear mixed effects model results for comparing representations taking graph representation as baseline model for 6/4 train/test splitting ratio and random splitting for the DAAA benchmark dataset.

| **Variable** | **Coef.** | **Std.Err.** | **z** | **P>\|z\|** | **[0.025** | **0.975]** |
| --- | --- | --- | --- | --- | --- | --- |
| Intercept | 2.339 | 0.138 | 16.920 | 0.000 | 2.068 | 2.610 |
| C(representation, Treatment(reference='Graph'))  [T.ChemBertA 77M-MLM] | -0.170 | 0.154 | -1.103 | 0.270 | -0.472 | 0.132 |
| C(representation, Treatment(reference='Graph'))  [T.Morgan 1024] | -0.339 | 0.154 | -2.203 | 0.028 | -0.641 | -0.037 |
| C(representation, Treatment(reference='Graph'))  [T.RDKit] | 0.135 | 0.154 | 0.877 | 0.381 | -0.167 | 0.437 |
| C(representation, Treatment(reference='Graph'))  [T.V4] | -0.314 | 0.154 | -2.041 | 0.041 | -0.616 | -0.013 |
| Group Var | 0.001 | 0.038 |  |  |  |  |

**Table S11.** Wilcoxon statistical tests for comparing non-graph ML algorithms MAE value distributions for 6/4 train/test splitting ratio and random splitting for the DAAA benchmark dataset.

| **group a** | **Group b** | **n** | **Wilcoxon stat** | **P value** | **Median diff a minus b** | **P value corrected** | **Reject null** |
| --- | --- | --- | --- | --- | --- | --- | --- |
| MLP | XGB | 20 | 18.0 | 0.000 | 0.560 | 0.003 | True |
| MLP | RF | 20 | 19.0 | 0.001 | 0.539 | 0.003 | True |
| MLP | SVR | 20 | 29.0 | 0.003 | 0.327 | 0.013 | True |
| SVR | XGB | 20 | 56.0 | 0.070 | 0.199 | 0.209 | False |
| RF | SVR | 20 | 57.0 | 0.076 | -0.169 | 0.209 | False |
| RF | XGB | 20 | 105.0 | 1.000 | 0.000 | 1.000 | False |

**Table S12.** Linear mixed effects model results for comparing ML algorithms taking GIN as baseline model for 4/6 train/test splitting ratio and random splitting for the DAAA benchmark dataset.

| **Variable** | **Coef.** | **Std.Err.** | **z** | **P>\|z\|** | **[0.025** | **0.975]** |
| --- | --- | --- | --- | --- | --- | --- |
| Intercept | 2.339 | 0.140 | 16.684 | 0.000 | 2.065 | 2.614 |
| C(model, Treatment(reference='GIN'))[T.MLP] | 0.112 | 0.157 | 0.714 | 0.475 | -0.195 | 0.419 |
| C(model, Treatment(reference='GIN'))[T.RF] | -0.329 | 0.157 | -2.100 | 0.036 | -0.637 | -0.022 |
| C(model, Treatment(reference='GIN'))[T.SVR] | -0.144 | 0.157 | -0.920 | 0.358 | -0.451 | 0.163 |
| C(model, Treatment(reference='GIN'))[T.XGB] | -0.327 | 0.157 | -2.085 | 0.037 | -0.634 | -0.020 |
| Group Var | 0.000 | 0.026 |  |  |  |  |

## 6.3 Train/test 8/2 ratio splitting

**Table S13.** Raw MAE values of different representation – ML algorithm combinations for random splitting trials and 8/2 train/test ratio for the DAAA benchmark dataset.

| **Random seed** | **0** | **1** | **2** | **3** | **4** | **mean ± std** |
| --- | --- | --- | --- | --- | --- | --- |
| Morgan 1024 RF MAE mean | 1.487 | 1.47 | 1.356 | 1.328 | 1.316 | 1.39 ± 0.08 |
| V4 SVR MAE mean | 1.756 | 1.332 | 1.301 | 1.452 | 1.292 | 1.43 ± 0.19 |
| ChemBertA 77M-MLM XGB MAE mean | 1.484 | 1.548 | 1.609 | 1.427 | 1.426 | 1.50 ± 0.08 |
| ChemBertA 77M-MLM RF MAE mean | 1.703 | 1.55 | 1.497 | 1.51 | 1.484 | 1.55 ± 0.09 |
| Morgan 1024 XGB MAE mean | 1.721 | 1.557 | 1.507 | 1.434 | 1.64 | 1.57 ± 0.11 |
| RDKit RF MAE mean | 1.704 | 1.651 | 1.703 | 1.749 | 1.743 | 1.71 ± 0.04 |
| V4 XGB MAE mean | 1.765 | 1.777 | 1.68 | 1.689 | 1.637 | 1.71 ± 0.06 |
| RDKit XGB MAE mean | 2.011 | 1.748 | 1.965 | 1.646 | 1.723 | 1.82 ± 0.16 |
| V4 MLP MAE mean | 1.623 | 1.698 | 1.685 | 2.435 | 1.926 | 1.87 ± 0.33 |
| V4 RF MAE mean | 1.867 | 1.917 | 1.988 | 1.853 | 1.882 | 1.90 ± 0.05 |
| Morgan 1024 MLP MAE mean | 2.899 | 1.899 | 1.716 | 1.549 | 1.705 | 1.95 ± 0.54 |
| Morgan 1024 SVR MAE mean | 2.16 | 1.675 | 2.325 | 2.135 | 1.783 | 2.02 ± 0.27 |
| RDKit SVR MAE mean | 2.045 | 2.03 | 2.348 | 2.152 | 2.096 | 2.13 ± 0.13 |
| ChemBertA 77M-MLM MLP MAE mean | 1.592 | 1.986 | 2.568 | 2.472 | 2.139 | 2.15 ± 0.39 |
| Graph GIN MAE mean | 1.991 | 2.189 | 2.844 | 1.954 | 2.041 | 2.20 ± 0.37 |
| ChemBertA 77M-MLM SVR MAE mean | 2.491 | 2.26 | 2.408 | 2.189 | 1.908 | 2.25 ± 0.23 |
| RDKit MLP MAE mean | 2.73 | 2.602 | 2.792 | 1.927 | 2.25 | 2.46 ± 0.36 |

**Table S14.** Raw R^2^ values of different representation – ML algorithm combinations for random splitting trials and 8/2 train/test ratio for the DAAA benchmark dataset.

| **Random seed** | **0** | **1** | **2** | **3** | **4** | **mean ± std** |
| --- | --- | --- | --- | --- | --- | --- |
| Morgan 1024 RF R2 mean | 0.768 | 0.761 | 0.793 | 0.784 | 0.787 | 0.78 ± 0.01 |
| V4 SVR R2 mean | 0.619 | 0.808 | 0.794 | 0.772 | 0.81 | 0.76 ± 0.08 |
| Morgan 1024 XGB R2 mean | 0.689 | 0.737 | 0.725 | 0.777 | 0.722 | 0.73 ± 0.03 |
| ChemBertA 77M-MLM XGB R2 mean | 0.746 | 0.689 | 0.714 | 0.73 | 0.729 | 0.72 ± 0.02 |
| ChemBertA 77M-MLM RF R2 mean | 0.661 | 0.691 | 0.701 | 0.697 | 0.703 | 0.69 ± 0.02 |
| Morgan 1024 SVR R2 mean | 0.62 | 0.665 | 0.541 | 0.598 | 0.662 | 0.62 ± 0.05 |
| V4 MLP R2 mean | 0.691 | 0.699 | 0.669 | 0.334 | 0.625 | 0.60 ± 0.15 |
| V4 XGB R2 mean | 0.559 | 0.537 | 0.594 | 0.598 | 0.61 | 0.58 ± 0.03 |
| RDKit XGB R2 mean | 0.503 | 0.58 | 0.491 | 0.631 | 0.641 | 0.57 ± 0.07 |
| Morgan 1024 MLP R2 mean | 0.061 | 0.622 | 0.695 | 0.747 | 0.664 | 0.56 ± 0.28 |
| RDKit SVR R2 mean | 0.581 | 0.594 | 0.487 | 0.554 | 0.575 | 0.56 ± 0.04 |
| RDKit RF R2 mean | 0.569 | 0.591 | 0.553 | 0.554 | 0.555 | 0.56 ± 0.02 |
| ChemBertA 77M-MLM SVR R2 mean | 0.476 | 0.535 | 0.495 | 0.565 | 0.601 | 0.53 ± 0.05 |
| ChemBertA 77M-MLM MLP R2 mean | 0.716 | 0.569 | 0.344 | 0.36 | 0.514 | 0.50 ± 0.15 |
| Graph GIN R2 mean | 0.524 | 0.476 | 0.19 | 0.578 | 0.581 | 0.47 ± 0.16 |
| V4 RF R2 mean | 0.423 | 0.411 | 0.371 | 0.456 | 0.435 | 0.42 ± 0.03 |
| RDKit MLP R2 mean | 0.238 | 0.317 | 0.266 | 0.492 | 0.449 | 0.35 ± 0.11 |

**Table S15.** Wilcoxon statistical tests for comparing non-graph representations MAE value distributions for 8/2 train/test splitting ratio and random splitting for the DAAA benchmark dataset.

| **group a** | **Group b** | **n** | **Wilcoxon stat** | **P value** | **Median diff a minus b** | **P value corrected** | **Reject null** |
| --- | --- | --- | --- | --- | --- | --- | --- |
| Morgan 1024 | RDKit | 20 | 9.0 | 0.000 | -0.302 | 0.000 | True |
| ChemBertA 77M-MLM | Morgan 1024 | 20 | 49.0 | 0.036 | 0.114 | 0.060 | False |
| RDKit | V4 | 20 | 49.0 | 0.036 | 0.265 | 0.060 | False |
| ChemBertA 77M-MLM | RDKit | 20 | 50.0 | 0.040 | -0.203 | 0.060 | False |
| ChemBertA 77M-MLM | V4 | 20 | 87.0 | 0.522 | -0.051 | 0.626 | False |
| Morgan 1024 | V4 | 20 | 97.0 | 0.784 | -0.108 | 0.784 | False |

**Table S16.** Linear mixed effects model results for comparing representations taking graph representation as baseline model for 8/2 train/test splitting ratio and random splitting for the DAAA benchmark dataset.

| **Variable** | **Coef.** | **Std.Err.** | **z** | **P>\|z\|** | **[0.025** | **0.975]** |
| --- | --- | --- | --- | --- | --- | --- |
| Intercept | 2.204 | 0.161 | 13.653 | 0.000 | 1.887 | 2.520 |
| C(representation, Treatment(reference='Graph'))  [T.ChemBertA 77M-MLM] | -0.341 | 0.180 | -1.891 | 0.059 | -0.695 | 0.012 |
| C(representation, Treatment(reference='Graph'))  [T.Morgan 1024] | -0.471 | 0.180 | -2.608 | 0.009 | -0.824 | -0.117 |
| C(representation, Treatment(reference='Graph'))  [T.RDKit] | -0.173 | 0.180 | -0.959 | 0.337 | -0.527 | 0.181 |
| C(representation, Treatment(reference='Graph'))  [T.V4] | -0.476 | 0.180 | -2.638 | 0.008 | -0.830 | -0.122 |
| Group Var | 0.000 | 0.016 |  |  |  |  |

**Table S17.** Wilcoxon statistical tests for comparing non-graph ML algorithms MAE value distributions for 8/2 train/test splitting ratio and random splitting for the DAAA benchmark dataset.

| **Group a** | **Group b** | **n** | **Wilcoxon stat** | **P value** | **Median diff a minus b** | **P value corrected** | **Reject null** |
| --- | --- | --- | --- | --- | --- | --- | --- |
| MLP | XGB | 20 | 9.0 | 0.000 | 0.390 | 0.000 | True |
| MLP | RF | 20 | 19.0 | 0.001 | 0.432 | 0.003 | True |
| SVR | XGB | 20 | 34.0 | 0.006 | 0.378 | 0.026 | True |
| RF | SVR | 20 | 43.0 | 0.019 | -0.414 | 0.058 | False |
| MLP | SVR | 20 | 65.0 | 0.143 | 0.227 | 0.286 | False |
| RF | XGB | 20 | 98.0 | 0.812 | 0.011 | 0.812 | False |

**Table S18.** Linear mixed effects model results for comparing ML algorithms taking GIN as baseline model for 8/2 train/test splitting ratio and random splitting for the DAAA benchmark dataset.

| **Variable** | **Coef.** | **Std.Err.** | **z** | **P>\|z\|** | **[0.025** | **0.975]** |
| --- | --- | --- | --- | --- | --- | --- |
| Intercept | 2.204 | 0.145 | 15.222 | 0.000 | 1.920 | 2.488 |
| C(model, Treatment(reference='GIN'))[T.MLP] | -0.094 | 0.161 | -0.585 | 0.559 | -0.409 | 0.221 |
| C(model, Treatment(reference='GIN'))[T.RF] | -0.566 | 0.161 | -3.518 | 0.000 | -0.881 | -0.251 |
| C(model, Treatment(reference='GIN'))[T.SVR] | -0.247 | 0.161 | -1.535 | 0.125 | -0.562 | 0.068 |
| C(model, Treatment(reference='GIN'))[T.XGB] | -0.554 | 0.161 | -3.444 | 0.001 | -0.870 | -0.239 |
| Group Var | 0.001 | 0.017 |  |  |  |  |

## 6.4 Random splitting complementary figures

**Figure S3.** Distribution of test set MAE values for different reaction representation – ML algorithm pairs for random splitting for **a)** 4/6, **b)** 6/4, and **c)** 8/2 train/test splitting ratios. Distributions are ordered from lower to higher mean MAE.

**Figure S4.** Distribution of test set R^2^ values for different reaction representation – ML algorithm pairs for random splitting for **a)** 4/6, **b)** 6/4, and **c)** 8/2 train/test splitting ratios. Distributions are ordered from lower to higher mean R^2^.

# 7. Scaffold-based splitting raw data and supporting figures

## 7.1 Train/test 4/6 ratio splitting

**Table S19.** Raw MAE values of different representation – ML algorithm combinations for scaffold-based splitting trials and 4/6 train/test ratio for the DAAA benchmark dataset.

| **Random seed** | **0** | **1** | **2** | **3** | **4** | **mean ± std** |
| --- | --- | --- | --- | --- | --- | --- |
| Morgan 1024 XGB MAE mean | 3.036 | 2.543 | 2.721 | 2.849 | 2.951 | 2.82 ± 0.19 |
| Morgan 1024 RF MAE mean | 2.922 | 2.777 | 2.894 | 2.923 | 2.839 | 2.87 ± 0.06 |
| RDKit SVR MAE mean | 3.511 | 3.173 | 3.259 | 3.103 | 2.951 | 3.20 ± 0.21 |
| ChemBertA 77M-MLM SVR MAE mean | 3.082 | 3.085 | 3.501 | 3.184 | 3.426 | 3.26 ± 0.20 |
| Morgan 1024 SVR MAE mean | 3.492 | 3.329 | 3.592 | 3.345 | 3.356 | 3.42 ± 0.11 |
| RDKit RF MAE mean | 3.558 | 3.523 | 3.592 | 3.533 | 3.505 | 3.54 ± 0.03 |
| ChemBertA 77M-MLM RF MAE mean | 3.644 | 3.513 | 3.5 | 3.506 | 3.603 | 3.55 ± 0.07 |
| Morgan 1024 MLP MAE mean | 3.773 | 4.386 | 3.513 | 3.082 | 3.151 | 3.58 ± 0.53 |
| ChemBertA 77M-MLM XGB MAE mean | 3.556 | 3.439 | 3.624 | 3.506 | 3.901 | 3.61 ± 0.18 |
| RDKit XGB MAE mean | 3.646 | 3.84 | 3.59 | 3.665 | 3.571 | 3.66 ± 0.11 |
| ChemBertA 77M-MLM MLP MAE mean | 4.047 | 3.272 | 3.889 | 3.782 | 4.698 | 3.94 ± 0.51 |
| V4 RF MAE mean | 4.011 | 4.056 | 4.052 | 4.017 | 3.976 | 4.02 ± 0.03 |
| V4 XGB MAE mean | 4.465 | 3.923 | 3.753 | 4.292 | 3.911 | 4.07 ± 0.30 |
| Graph GIN MAE mean | 4.071 | 3.84 | 3.736 | 5.129 | 3.697 | 4.09 ± 0.60 |
| RDKit MLP MAE mean | 5.621 | 4.186 | 4.59 | 4.337 | 4.9 | 4.73 ± 0.57 |
| V4 MLP MAE mean | 6.102 | 7.017 | 8.566 | 3.891 | 4.003 | 5.92 ± 2.00 |
| V4 SVR MAE mean | 18.654 | 3.614 | 7.233 | 4.682 | 3.426 | 7.52 ± 6.41 |

**Table S20.** Raw R^2^ values of different representation – ML algorithm combinations for random scaffold-based trials and 4/6 train/test ratio for the DAAA benchmark dataset.

| **Random seed** | **0** | **1** | **2** | **3** | **4** | **mean ± std** |
| --- | --- | --- | --- | --- | --- | --- |
| Morgan 1024 XGB R2 mean | 0.124 | 0.319 | 0.212 | 0.183 | 0.113 | 0.19 ± 0.08 |
| Morgan 1024 RF R2 mean | 0.16 | 0.212 | 0.171 | 0.153 | 0.193 | 0.18 ± 0.02 |
| ChemBertA 77M-MLM SVR R2 mean | 0.221 | 0.217 | -0.015 | 0.18 | 0.01 | 0.12 ± 0.12 |
| RDKit SVR R2 mean | -0.156 | 0.125 | 0.043 | 0.169 | 0.201 | 0.08 ± 0.14 |
| V4 SVR R2 mean | -35.645 | -0.191 | -3.342 | -0.949 | -0.07 | -8.04 ± 15.49 |
| V4 MLP R2 mean | -2.348 | -5.941 | -10.024 | -0.442 | -0.753 | -3.90 ± 4.06 |
| RDKit MLP R2 mean | -4.436 | -1.245 | -1.921 | -0.649 | -1.195 | -1.89 ± 1.49 |
| Graph GIN R2 mean | -0.571 | -0.374 | -0.412 | -1.542 | -0.181 | -0.62 ± 0.54 |
| V4 RF R2 mean | -0.559 | -0.584 | -0.593 | -0.523 | -0.508 | -0.55 ± 0.04 |
| V4 XGB R2 mean | -0.907 | -0.45 | -0.282 | -0.647 | -0.391 | -0.54 ± 0.25 |
| ChemBertA 77M-MLM MLP R2 mean | -0.463 | -0.084 | -0.298 | -0.298 | -0.961 | -0.42 ± 0.33 |
| Morgan 1024 MLP R2 mean | -0.373 | -0.696 | -0.001 | 0.113 | 0.104 | -0.17 ± 0.35 |
| RDKit XGB R2 mean | -0.108 | -0.212 | -0.228 | -0.189 | -0.128 | -0.17 ± 0.05 |
| ChemBertA 77M-MLM XGB R2 mean | -0.094 | -0.01 | -0.105 | -0.021 | -0.353 | -0.12 ± 0.14 |
| RDKit RF R2 mean | -0.126 | -0.113 | -0.145 | -0.114 | -0.099 | -0.12 ± 0.02 |
| ChemBertA 77M-MLM RF R2 mean | -0.155 | -0.066 | -0.058 | -0.069 | -0.129 | -0.10 ± 0.04 |
| Morgan 1024 SVR R2 mean | -0.137 | 0.004 | -0.106 | -0.006 | 0.019 | -0.05 ± 0.07 |

**Table S21.** Wilcoxon statistical tests for comparing non-graph representations MAE value distributions for 4/6 train/test splitting ratio and scaffold-based splitting for the DAAA benchmark dataset.

| **group a** | **Group b** | **n** | **Wilcoxon stat** | **P value** | **Median diff a minus b** | **P value corrected** | **Reject null** |
| --- | --- | --- | --- | --- | --- | --- | --- |
| Morgan 1024 | V4 | 20 | 0.0 | 0.000 | -1.219 | 0.000 | True |
| ChemBertA 77M-MLM | V4 | 20 | 13.0 | 0.000 | -0.536 | 0.001 | True |
| Morgan 1024 | RDKit | 20 | 20.0 | 0.001 | -0.652 | 0.001 | True |
| RDKit | V4 | 20 | 20.0 | 0.001 | -0.478 | 0.001 | True |
| ChemBertA 77M-MLM | Morgan 1024 | 20 | 35.0 | 0.007 | 0.595 | 0.009 | True |
| ChemBertA 77M-MLM | RDKit | 20 | 62.0 | 0.114 | -0.089 | 0.114 | False |

**Table S22.** Linear mixed effects model results for comparing representations taking graph representation as baseline model for 4/6 train/test splitting ratio and scaffold-based splitting for the DAAA benchmark dataset.

| **Variable** | **Coef.** | **Std.Err.** | **z** | **P>\|z\|** | **[0.025** | **0.975]** |
| --- | --- | --- | --- | --- | --- | --- |
| Intercept | 4.095 | 0.773 | 5.298 | 0.000 | 2.580 | 5.609 |
| C(representation, Treatment(reference='Graph'))  [T.ChemBertA 77M-MLM] | -0.507 | 0.856 | -0.592 | 0.554 | -2.185 | 1.172 |
| C(representation, Treatment(reference='Graph'))  [T.Morgan 1024] | -0.921 | 0.856 | -1.076 | 0.282 | -2.599 | 0.757 |
| C(representation, Treatment(reference='Graph'))  [T.RDKit] | -0.312 | 0.856 | -0.364 | 0.716 | -1.990 | 1.366 |
| C(representation, Treatment(reference='Graph'))  [T.V4] | 1.288 | 0.856 | 1.504 | 0.133 | -0.391 | 2.966 |
| Group Var | 0.053 | 0.096 |  |  |  |  |

**Table S23.** Wilcoxon statistical tests for comparing non-graph ML algorithms MAE value distributions for 4/6 train/test splitting ratio and scaffold-based splitting for the DAAA benchmark dataset.

| **Group a** | **Group b** | **n** | **Wilcoxon stat** | **P value** | **Median diff a minus b** | **P value corrected** | **Reject null** |
| --- | --- | --- | --- | --- | --- | --- | --- |
| MLP | RF | 20 | 6.0 | 0.000 | 0.733 | 0.000 | True |
| MLP | XGB | 20 | 10.0 | 0.000 | 0.704 | 0.000 | True |
| MLP | SVR | 20 | 37.0 | 0.009 | 0.782 | 0.038 | True |
| RF | XGB | 20 | 84.0 | 0.452 | -0.033 | 1.000 | False |
| RF | SVR | 20 | 88.0 | 0.546 | 0.112 | 1.000 | False |
| SVR | XGB | 20 | 99.0 | 0.841 | -0.222 | 1.000 | False |

**Table S24.** Linear mixed effects model results for comparing ML algorithms taking GIN as baseline model for 4/6 train/test splitting ratio and scaffold-based splitting for the DAAA benchmark dataset.

| **Variable** | **Coef.** | **Std.Err.** | **z** | **P>\|z\|** | **[0.025** | **0.975]** |
| --- | --- | --- | --- | --- | --- | --- |
| Intercept | 4.095 | 0.832 | 4.919 | 0.000 | 2.463 | 5.726 |
| C(model, Treatment(reference='GIN'))[T.MLP] | 0.446 | 0.927 | 0.480 | 0.631 | -1.372 | 2.263 |
| C(model, Treatment(reference='GIN'))[T.RF] | -0.597 | 0.927 | -0.644 | 0.520 | -2.415 | 1.220 |
| C(model, Treatment(reference='GIN'))[T.SVR] | 0.255 | 0.927 | 0.275 | 0.783 | -1.562 | 2.073 |
| C(model, Treatment(reference='GIN'))[T.XGB] | -0.556 | 0.927 | -0.599 | 0.549 | -2.373 | 1.262 |
| Group Var | 0.023 | 0.088 |  |  |  |  |

## 7.2 Train/test 6/4 ratio splitting

**Table S25.** Raw MAE values of different representation – ML algorithm combinations for scaffold-based splitting trials and 6/4 train/test ratio for the DAAA benchmark dataset.

| **Random seed** | **0** | **1** | **2** | **3** | **4** | **mean ± std** |
| --- | --- | --- | --- | --- | --- | --- |
| Morgan 1024 RF MAE mean | 2.449 | 2.255 | 2.419 | 2.229 | 2.346 | 2.34 ± 0.10 |
| Morgan 1024 MLP MAE mean | 2.589 | 2.303 | 2.45 | 2.566 | 2.416 | 2.46 ± 0.12 |
| Morgan 1024 XGB MAE mean | 2.493 | 2.215 | 2.824 | 2.466 | 2.344 | 2.47 ± 0.23 |
| V4 SVR MAE mean | 2.64 | 2.677 | 2.885 | 2.562 | 2.887 | 2.73 ± 0.15 |
| ChemBertA 77M-MLM RF MAE mean | 2.745 | 2.757 | 2.739 | 2.827 | 2.614 | 2.74 ± 0.08 |
| Morgan 1024 SVR MAE mean | 2.955 | 2.829 | 2.91 | 2.911 | 2.909 | 2.90 ± 0.05 |
| ChemBertA 77M-MLM XGB MAE mean | 3.223 | 3.068 | 3.047 | 3.216 | 2.587 | 3.03 ± 0.26 |
| ChemBertA 77M-MLM SVR MAE mean | 2.989 | 2.607 | 3.155 | 3.246 | 3.202 | 3.04 ± 0.26 |
| RDKit RF MAE mean | 3.496 | 3.486 | 3.479 | 3.462 | 3.446 | 3.47 ± 0.02 |
| V4 RF MAE mean | 3.56 | 3.508 | 3.462 | 3.454 | 3.411 | 3.48 ± 0.06 |
| ChemBertA 77M-MLM MLP MAE mean | 3.425 | 4.459 | 2.93 | 3.313 | 3.354 | 3.50 ± 0.57 |
| RDKit XGB MAE mean | 3.613 | 3.429 | 3.552 | 3.598 | 3.515 | 3.54 ± 0.07 |
| RDKit SVR MAE mean | 3.741 | 3.25 | 3.572 | 3.66 | 3.485 | 3.54 ± 0.19 |
| V4 MLP MAE mean | 5.448 | 3.834 | 2.63 | 3.536 | 3.737 | 3.84 ± 1.02 |
| V4 XGB MAE mean | 3.895 | 3.855 | 4.421 | 3.903 | 3.748 | 3.96 ± 0.26 |
| Graph GIN MAE mean | 3.422 | 7.176 | 5.797 | 2.693 | 5.231 | 4.86 ± 1.81 |
| RDKit MLP MAE mean | 7.394 | 5.712 | 4.813 | 6.681 | 5.0 | 5.92 ± 1.10 |

**Table S26.** Raw R^2^ values of different representation – ML algorithm combinations for random scaffold-based trials and 6/4 train/test ratio for the DAAA benchmark dataset.

| **Random seed** | **0** | **1** | **2** | **3** | **4** | **mean ± std** |
| --- | --- | --- | --- | --- | --- | --- |
| Morgan 1024 RF R2 mean | 0.484 | 0.547 | 0.502 | 0.561 | 0.518 | 0.52 ± 0.03 |
| Morgan 1024 MLP R2 mean | 0.496 | 0.544 | 0.497 | 0.412 | 0.533 | 0.50 ± 0.05 |
| Morgan 1024 XGB R2 mean | 0.405 | 0.484 | 0.314 | 0.387 | 0.451 | 0.41 ± 0.06 |
| ChemBertA 77M-MLM RF R2 mean | 0.417 | 0.398 | 0.415 | 0.378 | 0.46 | 0.41 ± 0.03 |
| V4 SVR R2 mean | 0.415 | 0.406 | 0.255 | 0.366 | 0.307 | 0.35 ± 0.07 |
| Morgan 1024 SVR R2 mean | 0.311 | 0.371 | 0.367 | 0.346 | 0.351 | 0.35 ± 0.02 |
| ChemBertA 77M-MLM XGB R2 mean | 0.252 | 0.332 | 0.34 | 0.271 | 0.483 | 0.34 ± 0.09 |
| ChemBertA 77M-MLM SVR R2 mean | 0.357 | 0.501 | 0.292 | 0.252 | 0.248 | 0.33 ± 0.11 |
| ChemBertA 77M-MLM MLP R2 mean | -0.005 | -0.296 | 0.399 | 0.079 | 0.16 | 0.07 ± 0.25 |
| RDKit RF R2 mean | 0.058 | 0.049 | 0.047 | 0.089 | 0.068 | 0.06 ± 0.02 |
| RDKit XGB R2 mean | -0.004 | 0.085 | -0.054 | 0.06 | 0.115 | 0.04 ± 0.07 |
| RDKit SVR R2 mean | -0.01 | 0.119 | 0.041 | 0.018 | 0.048 | 0.04 ± 0.05 |
| RDKit MLP R2 mean | -4.403 | -2.329 | -1.017 | -5.678 | -1.148 | -2.92 ± 2.06 |
| Graph GIN R2 mean | 0.138 | -2.696 | -1.087 | 0.358 | -1.074 | -0.87 ± 1.22 |
| V4 MLP R2 mean | -1.745 | -0.162 | 0.298 | -0.028 | -0.253 | -0.38 ± 0.79 |
| V4 XGB R2 mean | -0.294 | -0.132 | -0.613 | -0.204 | -0.125 | -0.27 ± 0.20 |
| V4 RF R2 mean | -0.106 | -0.011 | -0.03 | 0.059 | 0.05 | -0.01 ± 0.07 |

**Table S27.** Wilcoxon statistical tests for comparing non-graph representations MAE value distributions for 6/4 train/test splitting ratio and scaffold-based splitting for the DAAA benchmark dataset.

| **group a** | **Group b** | **n** | **Wilcoxon stat** | **P value** | **Median diff a minus b** | **P value corrected** | **Reject null** |
| --- | --- | --- | --- | --- | --- | --- | --- |
| ChemBertA 77M-MLM | RDKit | 20 | 0.0 | 0.000 | -0.734 | 0.000 | True |
| Morgan 1024 | RDKit | 20 | 0.0 | 0.000 | -1.126 | 0.000 | True |
| ChemBertA 77M-MLM | Morgan 1024 | 20 | 2.0 | 0.000 | 0.408 | 0.000 | True |
| Morgan 1024 | V4 | 20 | 17.0 | 0.000 | -1.168 | 0.001 | True |
| ChemBertA 77M-MLM | V4 | 20 | 37.0 | 0.009 | -0.650 | 0.011 | True |
| RDKit | V4 | 20 | 51.0 | 0.044 | 0.304 | 0.044 | True |

**Table S28.** Linear mixed effects model results for comparing representations taking graph representation as baseline model for 6/4 train/test splitting ratio and scaffold-based splitting for the DAAA benchmark dataset.

| **Variable** | **Coef.** | **Std.Err.** | **z** | **P>\|z\|** | **[0.025** | **0.975]** |
| --- | --- | --- | --- | --- | --- | --- |
| Intercept | 4.864 | 0.366 | 13.297 | 0.000 | 4.147 | 5.581 |
| C(representation, Treatment(reference='Graph'))  [T.ChemBertA 77M-MLM] | -1.789 | 0.409 | -4.373 | 0.000 | -2.590 | -0.987 |
| C(representation, Treatment(reference='Graph'))  [T.Morgan 1024] | -2.320 | 0.409 | -5.673 | 0.000 | -3.121 | -1.518 |
| C(representation, Treatment(reference='Graph'))  [T.RDKit] | -0.745 | 0.409 | -1.821 | 0.069 | -1.546 | 0.057 |
| C(representation, Treatment(reference='Graph'))  [T.V4] | -1.361 | 0.409 | -3.328 | 0.001 | -2.163 | -0.559 |
| Group Var | 0.053 | 0.096 |  |  |  |  |

**Table S29.** Wilcoxon statistical tests for comparing non-graph ML algorithms MAE value distributions for 6/4 train/test splitting ratio and scaffold-based splitting for the DAAA benchmark dataset.

| **Group a** | **Group b** | **n** | **Wilcoxon stat** | **P value** | **Median diff a minus b** | **P value corrected** | **Reject null** |
| --- | --- | --- | --- | --- | --- | --- | --- |
| RF | XGB | 20 | 11.0 | 0.000 | -0.272 | 0.001 | True |
| MLP | RF | 20 | 13.0 | 0.000 | 0.411 | 0.001 | True |
| MLP | SVR | 20 | 45.0 | 0.024 | 0.643 | 0.096 | False |
| MLP | XGB | 20 | 49.0 | 0.036 | 0.098 | 0.109 | False |
| SVR | XGB | 20 | 90.0 | 0.596 | 0.025 | 1.000 | False |
| RF | SVR | 20 | 92.0 | 0.648 | -0.221 | 1.000 | False |

**Table S30.** Linear mixed effects model results for comparing ML algorithms taking GIN as baseline model for 6/4 train/test splitting ratio and scaffold-based splitting for the DAAA benchmark dataset.

| **Variable** | **Coef.** | **Std.Err.** | **z** | **P>\|z\|** | **[0.025** | **0.975]** |
| --- | --- | --- | --- | --- | --- | --- |
| Intercept | 4.864 | 0.416 | 11.685 | 0.000 | 4.048 | 5.680 |
| C(model, Treatment(reference='GIN'))[T.MLP] | -0.934 | 0.465 | -2.007 | 0.045 | -1.846 | -0.022 |
| C(model, Treatment(reference='GIN'))[T.RF] | -1.857 | 0.465 | -3.989 | 0.000 | -2.769 | -0.944 |
| C(model, Treatment(reference='GIN'))[T.SVR] | -1.810 | 0.465 | -3.890 | 0.000 | -2.722 | -0.898 |
| C(model, Treatment(reference='GIN'))[T.XGB] | -1.613 | 0.465 | -3.466 | 0.001 | -2.525 | -0.701 |
| Group Var | 0.000 |  |  |  |  |  |

## 7.3 Train/test 8/2 ratio splitting

**Table S31.** Raw MAE values of different representation – ML algorithm combinations for scaffold-based splitting trials and 8/2 train/test ratio for the DAAA benchmark dataset.

| **Random seed** | **0** | **1** | **2** | **3** | **4** | **mean ± std** |
| --- | --- | --- | --- | --- | --- | --- |
| Morgan 1024 RF MAE mean | 1.78 | 1.818 | 1.785 | 1.713 | 1.756 | 1.77 ± 0.04 |
| Morgan 1024 XGB MAE mean | 1.581 | 1.639 | 1.92 | 1.521 | 2.332 | 1.80 ± 0.34 |
| Morgan 1024 SVR MAE mean | 2.256 | 2.303 | 1.814 | 1.802 | 2.591 | 2.15 ± 0.34 |
| V4 RF MAE mean | 2.197 | 2.27 | 2.289 | 2.158 | 2.209 | 2.22 ± 0.05 |
| V4 SVR MAE mean | 2.142 | 2.131 | 2.503 | 2.147 | 2.298 | 2.24 ± 0.16 |
| Morgan 1024 MLP MAE mean | 2.387 | 1.942 | 2.282 | 2.623 | 2.079 | 2.26 ± 0.27 |
| V4 MLP MAE mean | 2.41 | 2.275 | 2.435 | 2.576 | 2.203 | 2.38 ± 0.15 |
| Graph GIN MAE mean | 2.921 | 2.67 | 2.529 | 2.305 | 1.721 | 2.43 ± 0.45 |
| ChemBertA 77M-MLM RF MAE mean | 2.478 | 2.636 | 2.535 | 2.534 | 2.436 | 2.52 ± 0.08 |
| ChemBertA 77M-MLM XGB MAE mean | 2.542 | 2.932 | 2.328 | 2.658 | 3.068 | 2.71 ± 0.30 |
| V4 XGB MAE mean | 2.806 | 2.984 | 2.672 | 2.825 | 2.663 | 2.79 ± 0.13 |
| ChemBertA 77M-MLM SVR MAE mean | 3.912 | 4.059 | 3.879 | 3.573 | 4.071 | 3.90 ± 0.20 |
| RDKit XGB MAE mean | 4.693 | 4.342 | 4.875 | 4.053 | 4.757 | 4.54 ± 0.34 |
| RDKit RF MAE mean | 4.77 | 4.535 | 4.661 | 4.738 | 4.726 | 4.69 ± 0.09 |
| ChemBertA 77M-MLM MLP MAE mean | 4.298 | 4.348 | 3.12 | 4.744 | 8.059 | 4.91 ± 1.86 |
| RDKit SVR MAE mean | 8.36 | 6.354 | 4.925 | 5.203 | 5.58 | 6.08 ± 1.38 |
| RDKit MLP MAE mean | 8.824 | 14.581 | 6.549 | 12.92 | 6.186 | 9.81 ± 3.78 |

**Table S32.** Raw R^2^ values of different representation – ML algorithm combinations for random scaffold-based trials and 8/2 train/test ratio for the DAAA benchmark dataset.

| **Random seed** | **0** | **1** | **2** | **3** | **4** | **mean ± std** |
| --- | --- | --- | --- | --- | --- | --- |
| Morgan 1024 RF R2 mean | 0.78 | 0.77 | 0.771 | 0.779 | 0.776 | 0.78 ± 0.00 |
| Morgan 1024 XGB R2 mean | 0.805 | 0.787 | 0.752 | 0.832 | 0.599 | 0.76 ± 0.09 |
| Morgan 1024 MLP R2 mean | 0.606 | 0.736 | 0.673 | 0.596 | 0.718 | 0.67 ± 0.06 |
| Morgan 1024 SVR R2 mean | 0.678 | 0.65 | 0.718 | 0.727 | 0.542 | 0.66 ± 0.07 |
| Graph GIN R2 mean | 0.417 | 0.492 | 0.548 | 0.604 | 0.804 | 0.57 ± 0.15 |
| ChemBertA 77M-MLM RF R2 mean | 0.501 | 0.431 | 0.473 | 0.477 | 0.503 | 0.48 ± 0.03 |
| V4 RF R2 mean | 0.476 | 0.457 | 0.432 | 0.489 | 0.473 | 0.47 ± 0.02 |
| ChemBertA 77M-MLM XGB R2 mean | 0.509 | 0.358 | 0.586 | 0.498 | 0.32 | 0.45 ± 0.11 |
| V4 SVR R2 mean | 0.483 | 0.484 | 0.343 | 0.502 | 0.4 | 0.44 ± 0.07 |
| V4 MLP R2 mean | 0.358 | 0.399 | 0.324 | 0.277 | 0.455 | 0.36 ± 0.07 |
| V4 XGB R2 mean | 0.305 | 0.269 | 0.33 | 0.276 | 0.374 | 0.31 ± 0.04 |
| ChemBertA 77M-MLM SVR R2 mean | 0.062 | 0.004 | 0.028 | 0.301 | -0.013 | 0.08 ± 0.13 |
| RDKit MLP R2 mean | -4.011 | -19.308 | -1.721 | -11.953 | -1.724 | -7.74 ± 7.71 |
| RDKit SVR R2 mean | -2.812 | -1.567 | -0.718 | -0.849 | -1.13 | -1.41 ± 0.85 |
| ChemBertA 77M-MLM MLP R2 mean | -0.056 | -0.172 | 0.427 | -0.34 | -2.86 | -0.60 ± 1.29 |
| RDKit RF R2 mean | -0.387 | -0.295 | -0.345 | -0.389 | -0.373 | -0.36 ± 0.04 |
| RDKit XGB R2 mean | -0.33 | -0.271 | -0.4 | -0.086 | -0.414 | -0.30 ± 0.13 |

**Table S33.** Wilcoxon statistical tests for comparing non-graph representations MAE value distributions for 8/2 train/test splitting ratio and scaffold-based splitting for the DAAA benchmark dataset.

| **group a** | **Group b** | **n** | **Wilcoxon stat** | **P value** | **Median diff a minus b** | **P value corrected** | **Reject null** |
| --- | --- | --- | --- | --- | --- | --- | --- |
| ChemBertA 77M-MLM | Morgan 1024 | 20 | 0.0 | 0.0 | 1.215 | 0.0 | True |
| Morgan 1024 | RDKit | 20 | 0.0 | 0.0 | -3.068 | 0.0 | True |
| RDKit | V4 | 20 | 0.0 | 0.0 | 2.577 | 0.0 | True |
| ChemBertA 77M-MLM | RDKit | 20 | 7.0 | 0.0 | -2.178 | 0.0 | True |
| ChemBertA 77M-MLM | V4 | 20 | 15.0 | 0.0 | 0.545 | 0.0 | True |
| Morgan 1024 | V4 | 20 | 18.0 | 0.0 | -0.382 | 0.0 | True |

**Table S34.** Linear mixed effects model results for comparing representations taking graph representation as baseline model for 8/2 train/test splitting ratio and scaffold-based splitting for the DAAA benchmark dataset.

| **Variable** | **Coef.** | **Std.Err.** | **z** | **P>\|z\|** | **[0.025** | **0.975]** |
| --- | --- | --- | --- | --- | --- | --- |
| Intercept | 2.429 | 0.695 | 3.497 | 0.000 | 1.068 | 3.790 |
| C(representation, Treatment(reference='Graph'))  [T.ChemBertA 77M-MLM] | 1.081 | 0.777 | 1.393 | 0.164 | -0.441 | 2.603 |
| C(representation, Treatment(reference='Graph'))  [T.Morgan 1024] | -0.433 | 0.777 | -0.558 | 0.577 | -1.955 | 1.089 |
| C(representation, Treatment(reference='Graph'))  [T.RDKit] | 3.853 | 0.777 | 4.961 | 0.000 | 2.331 | 5.375 |
| C(representation, Treatment(reference='Graph'))  [T.V4] | -0.019 | 0.777 | -0.025 | 0.980 | -1.541 | 1.503 |
| Group Var | 0.000 |  |  |  |  |  |

**Table S35.** Wilcoxon statistical tests for comparing non-graph ML algorithms MAE value distributions for 8/2 train/test splitting ratio and scaffold-based splitting for the DAAA benchmark dataset.

| **Group a** | **Group b** | **n** | **Wilcoxon stat** | **P value** | **Median diff a minus b** | **P value corrected** | **Reject null** |
| --- | --- | --- | --- | --- | --- | --- | --- |
| MLP | RF | 20 | 2.0 | 0.000 | 0.758 | 0.000 | True |
| RF | SVR | 20 | 10.0 | 0.000 | -0.481 | 0.000 | True |
| MLP | XGB | 20 | 27.0 | 0.002 | 0.954 | 0.009 | True |
| MLP | SVR | 20 | 36.0 | 0.008 | 0.408 | 0.025 | True |
| SVR | XGB | 20 | 40.0 | 0.014 | 0.670 | 0.027 | True |
| RF | XGB | 20 | 62.0 | 0.114 | -0.129 | 0.114 | False |

**Table S36.** Linear mixed effects model results for comparing ML algorithms taking GIN as baseline model for 8/2 train/test splitting ratio and scaffold-based splitting for the DAAA benchmark dataset.

| **Variable** | **Coef.** | **Std.Err.** | **z** | **P>\|z\|** | **[0.025** | **0.975]** |
| --- | --- | --- | --- | --- | --- | --- |
| Intercept | 2.429 | 0.955 | 2.543 | 0.011 | 0.557 | 4.301 |
| C(model, Treatment(reference='GIN'))[T.MLP] | 2.413 | 1.068 | 2.260 | 0.024 | 0.320 | 4.506 |
| C(model, Treatment(reference='GIN'))[T.RF] | 0.372 | 1.068 | 0.348 | 0.727 | -1.721 | 2.465 |
| C(model, Treatment(reference='GIN'))[T.SVR] | 1.166 | 1.068 | 1.092 | 0.275 | -0.927 | 3.259 |
| C(model, Treatment(reference='GIN'))[T.XGB] | 0.530 | 1.068 | 0.497 | 0.619 | -1.563 | 2.624 |
| Group Var | 0.000 |  |  |  |  |  |

## 7.4 Scaffold-based splitting complementary figures

**Figure S5.** Distribution of test set MAE values for different reaction representation – ML algorithm pairs for random splitting for **a)** 4/6, **b)** 6/4, and **c)** 8/2 train/test splitting ratios. Distributions are ordered from lower to higher mean MAE.

**Figure S6.** Distribution of test set R^2^ values for different reaction representation – ML algorithm pairs for random splitting for **a)** 4/6, **b)** 6/4, and **c)** 8/2 train/test splitting ratios. Distributions are ordered from lower to higher mean R^2^.

**Figure S7.** Distribution of test set R^2^ values for different reaction representation – ML algorithm pairs for random splitting for **a)** 4/6, **b)** 6/4, and **c)** 8/2 train/test splitting ratios. Distributions are ordered from lower to higher mean R^2^. Only positive values of R2 are shown.

# 8. Target variable-based splitting raw data

**Table S37.** Raw R^2^ values of different representation – ML algorithm combinations for target variable-based split trials and 8/2 train/test ratio for the DAAA benchmark dataset.

| **Random seed** | **0** | **1** | **2** | **3** | **4** | **mean ± std** |
| --- | --- | --- | --- | --- | --- | --- |
| RDKit XGB R2 mean | -8.264 | -8.394 | -8.052 | -7.854 | -7.514 | -8.02 ± 0.35 |
| RDKit RF R2 mean | -7.614 | -8.333 | -7.714 | -7.874 | -7.736 | -7.85 ± 0.28 |
| RDKit SVR R2 mean | -6.618 | -6.168 | -5.649 | -7.014 | -6.109 | -6.31 ± 0.52 |
| V4 XGB R2 mean | -5.894 | -5.693 | -4.983 | -5.782 | -5.908 | -5.65 ± 0.38 |
| ChemBertA 77M-MLM MLP R2 mean | -7.027 | -5.7 | -5.005 | -4.966 | -5.464 | -5.63 ± 0.84 |
| Morgan 1024 XGB R2 mean | -5.998 | -5.42 | -5.467 | -5.421 | -5.284 | -5.52 ± 0.28 |
| ChemBertA 77M-MLM XGB R2 mean | -5.152 | -6.576 | -5.345 | -5.806 | -4.422 | -5.46 ± 0.80 |
| ChemBertA 77M-MLM RF R2 mean | -5.107 | -5.389 | -5.179 | -6.141 | -5.219 | -5.41 ± 0.42 |
| V4 RF R2 mean | -5.28 | -5.371 | -5.435 | -5.326 | -5.366 | -5.36 ± 0.06 |
| Morgan 1024 SVR R2 mean | -5.448 | -4.696 | -5.658 | -5.185 | -5.297 | -5.26 ± 0.36 |
| ChemBertA 77M-MLM SVR R2 mean | -6.11 | -4.446 | -5.118 | -4.243 | -6.185 | -5.22 ± 0.91 |
| Morgan 1024 MLP R2 mean | -4.896 | -4.395 | -7.029 | -4.885 | -4.845 | -5.21 ± 1.04 |
| Morgan 1024 RF R2 mean | -4.979 | -5.119 | -5.106 | -5.214 | -4.95 | -5.07 ± 0.11 |
| RDKit MLP R2 mean | -5.136 | -4.971 | -5.522 | -4.724 | -4.111 | -4.89 ± 0.52 |
| V4 SVR R2 mean | -4.809 | -4.635 | -4.459 | -4.642 | -4.318 | -4.57 ± 0.19 |
| V4 MLP R2 mean | -4.36 | -4.249 | -4.321 | -3.775 | -3.92 | -4.13 ± 0.26 |
| Graph GIN R2 mean | -2.179 | -3.936 | -4.045 | -2.698 | -4.12 | -3.40 ± 0.90 |

# 9. External validation dataset raw data and complimentary figures

## 9.1 Raw data external validation test set

**Table S37.** Raw MAE values of different representation – ML algorithm combinations for the DAAA external validation dataset when training models using the DAAA benchmark dataset.

| **Random seed** | **0** | **1** | **2** | **3** | **4** | **mean ± std** |
| --- | --- | --- | --- | --- | --- | --- |
| V4 RF MAE mean | 1.719 | 1.923 | 1.973 | 1.805 | 2.132 | 1.91 ± 0.16 |
| Morgan 1024 RF MAE mean | 1.891 | 1.871 | 1.891 | 2.037 | 1.92 | 1.92 ± 0.07 |
| Morgan 1024 XGB MAE mean | 1.944 | 1.937 | 1.924 | 2.38 | 2.298 | 2.10 ± 0.22 |
| V4 XGB MAE mean | 2.206 | 2.126 | 2.079 | 2.128 | 2.136 | 2.13 ± 0.05 |
| ChemBertA 77M-MLM RF MAE mean | 2.087 | 2.188 | 2.109 | 2.245 | 2.229 | 2.17 ± 0.07 |
| ChemBertA 77M-MLM MLP MAE mean | 2.374 | 2.16 | 1.584 | 2.224 | 2.765 | 2.22 ± 0.43 |
| Morgan 1024 SVR MAE mean | 1.936 | 3.637 | 2.017 | 1.94 | 1.918 | 2.29 ± 0.75 |
| ChemBertA 77M-MLM SVR MAE mean | 2.268 | 2.096 | 2.462 | 2.429 | 2.366 | 2.32 ± 0.15 |
| ChemBertA 77M-MLM XGB MAE mean | 2.334 | 2.261 | 2.227 | 2.37 | 2.656 | 2.37 ± 0.17 |
| Graph GIN MAE mean | 2.084 | 2.893 | 2.131 | 2.476 | 2.905 | 2.50 ± 0.40 |
| RDKit RF MAE mean | 2.547 | 2.752 | 2.55 | 2.551 | 2.603 | 2.60 ± 0.09 |
| RDKit SVR MAE mean | 2.366 | 4.12 | 2.241 | 2.212 | 2.405 | 2.67 ± 0.82 |
| Morgan 1024 MLP MAE mean | 2.265 | 2.702 | 2.752 | 2.539 | 3.395 | 2.73 ± 0.42 |
| RDKit XGB MAE mean | 2.847 | 2.691 | 3.082 | 2.556 | 3.034 | 2.84 ± 0.22 |
| V4 SVR MAE mean | 3.159 | 3.031 | 3.259 | 3.004 | 3.219 | 3.13 ± 0.11 |
| RDKit MLP MAE mean | 2.803 | 4.348 | 2.571 | 2.841 | 3.138 | 3.14 ± 0.70 |
| V4 MLP MAE mean | 4.558 | 6.744 | 3.003 | 4.427 | 4.193 | 4.59 ± 1.36 |

**Table S38.** Raw R^2^ values of different representation – ML algorithm combinations for the DAAA external validation dataset when training models using the DAAA benchmark dataset.

| **Random seed** | **0** | **1** | **2** | **3** | **4** | **mean ± std** |
| --- | --- | --- | --- | --- | --- | --- |
| RDKit MLP R2 mean | 0.127 | -0.934 | 0.305 | 0.193 | 0.092 | -0.04 ± 0.50 |
| V4 MLP R2 mean | -0.924 | -3.042 | 0.031 | -0.871 | -0.562 | -1.07 ± 1.16 |
| RDKit XGB R2 mean | 0.166 | 0.211 | 0.022 | 0.25 | 0.032 | 0.14 ± 0.10 |
| V4 SVR R2 mean | 0.158 | 0.141 | 0.113 | 0.182 | 0.131 | 0.15 ± 0.03 |
| RDKit RF R2 mean | 0.232 | 0.161 | 0.239 | 0.216 | 0.214 | 0.21 ± 0.03 |
| Morgan 1024 MLP R2 mean | 0.484 | 0.194 | 0.188 | 0.247 | 0.004 | 0.22 ± 0.17 |
| RDKit SVR R2 mean | 0.468 | -0.496 | 0.505 | 0.509 | 0.379 | 0.27 ± 0.43 |
| Graph GIN R2 mean | 0.447 | 0.338 | 0.349 | 0.283 | 0.069 | 0.30 ± 0.14 |
| ChemBertA 77M-MLM XGB R2 mean | 0.394 | 0.391 | 0.441 | 0.364 | 0.277 | 0.37 ± 0.06 |
| ChemBertA 77M-MLM SVR R2 mean | 0.428 | 0.504 | 0.233 | 0.335 | 0.365 | 0.37 ± 0.10 |
| Morgan 1024 SVR R2 mean | 0.522 | -0.144 | 0.461 | 0.515 | 0.496 | 0.37 ± 0.29 |
| ChemBertA 77M-MLM RF R2 mean | 0.47 | 0.411 | 0.405 | 0.377 | 0.409 | 0.41 ± 0.03 |
| Morgan 1024 XGB R2 mean | 0.575 | 0.488 | 0.484 | 0.265 | 0.339 | 0.43 ± 0.13 |
| ChemBertA 77M-MLM MLP R2 mean | 0.462 | 0.521 | 0.642 | 0.501 | 0.209 | 0.47 ± 0.16 |
| V4 XGB R2 mean | 0.453 | 0.516 | 0.55 | 0.531 | 0.524 | 0.51 ± 0.04 |
| Morgan 1024 RF R2 mean | 0.565 | 0.576 | 0.542 | 0.48 | 0.521 | 0.54 ± 0.04 |
| V4 RF R2 mean | 0.624 | 0.563 | 0.536 | 0.599 | 0.479 | 0.56 ± 0.06 |

**Table S39.** Wilcoxon statistical tests for comparing non-graph representations MAE value distributions for the DAAA external validation dataset.

| **Group a** | **Group b** | **n** | **Wilcoxon stat** | **P value** | **Median diff a minus b** | **P value corrected** | **Reject null** |
| --- | --- | --- | --- | --- | --- | --- | --- |
| Morgan 1024 | RDKit | 20 | 6.0 | 0.000 | -0.525 | 0.000 | True |
| ChemBertA 77M-MLM | RDKit | 20 | 9.0 | 0.000 | -0.430 | 0.000 | True |
| Morgan 1024 | V4 | 20 | 39.0 | 0.012 | -0.231 | 0.048 | True |
| ChemBertA 77M-MLM | V4 | 20 | 55.0 | 0.064 | -0.239 | 0.191 | False |
| ChemBertA 77M-MLM | Morgan 1024 | 20 | 83.0 | 0.430 | 0.261 | 0.861 | False |
| RDKit | V4 | 20 | 93.0 | 0.674 | 0.449 | 0.861 | False |

**Table S40.** Linear mixed effects model results for comparing representations taking graph representation as baseline model for the DAAA external validation dataset.

| **Variable** | **Coef.** | **Std.Err.** | **z** | **P>\|z\|** | **[0.025** | **0.975]** |
| --- | --- | --- | --- | --- | --- | --- |
| Intercept | 2.498 | 0.326 | 7.666 | 0.000 | 1.859 | 3.137 |
| C(representation, Treatment(reference='Graph'))  [T.ChemBertA 77M-MLM] | -0.226 | 0.357 | -0.633 | 0.527 | -0.926 | 0.474 |
| C(representation, Treatment(reference='Graph'))  [T.Morgan 1024] | -0.238 | 0.357 | -0.667 | 0.505 | -0.938 | 0.462 |
| C(representation, Treatment(reference='Graph'))  [T.RDKit] | 0.315 | 0.357 | 0.882 | 0.378 | -0.385 | 1.015 |
| C(representation, Treatment(reference='Graph'))  [T.V4] | 0.443 | 0.357 | 1.241 | 0.215 | -0.257 | 1.143 |
| Group Var | 0.021 | 0.051 |  |  |  |  |

**Table S41.** Wilcoxon statistical tests for comparing non-graph ML algorithms MAE value distributions for the DAAA external validation dataset.

| **Group a** | **Group b** | **n** | **Wilcoxon stat** | **P value** | **Median diff a minus b** | **P value corrected** | **Reject null** |
| --- | --- | --- | --- | --- | --- | --- | --- |
| RF | XGB | 20 | 5.0 | 0.000 | -0.164 | 0.000 | True |
| MLP | RF | 20 | 14.0 | 0.000 | 0.536 | 0.001 | True |
| MLP | XGB | 20 | 32.0 | 0.005 | 0.303 | 0.019 | True |
| MLP | SVR | 20 | 37.0 | 0.009 | 0.418 | 0.028 | True |
| RF | SVR | 20 | 49.0 | 0.036 | -0.159 | 0.072 | False |
| SVR | XGB | 20 | 79.0 | 0.349 | 0.026 | 0.349 | False |

**Table S42.** Linear mixed effects model results for comparing ML algorithms taking GIN as baseline model for the DAAA external validation dataset.

| **Variable** | **Coef.** | **Std.Err.** | **z** | **P>\|z\|** | **[0.025** | **0.975]** |
| --- | --- | --- | --- | --- | --- | --- |
| Intercept | 2.498 | 0.310 | 8.046 | 0.000 | 1.889 | 3.106 |
| C(model, Treatment(reference='GIN'))[T.MLP] | 0.671 | 0.338 | 1.984 | 0.047 | 0.008 | 1.335 |
| C(model, Treatment(reference='GIN'))[T.RF] | -0.347 | 0.338 | -1.025 | 0.306 | -1.010 | 0.317 |
| C(model, Treatment(reference='GIN'))[T.SVR] | 0.106 | 0.338 | 0.315 | 0.753 | -0.557 | 0.770 |
| C(model, Treatment(reference='GIN'))[T.XGB] | -0.137 | 0.338 | -0.405 | 0.686 | -0.800 | 0.526 |
| Group Var | 0.024 | 0.054 |  |  |  |  |

## 9.2 Complementary figures

**Figure S8.** Distribution of external validation MAE values for different reaction representation – ML algorithm pairs. Distributions are ordered from lower to higher mean MAE.

**Figure S9.** Distribution of external validation R^2^ values for different reaction representation – ML algorithm pairs. **a)** Shows all collected R^2^ values and **b)** shows only positive values.

Distributions are ordered from lower to higher mean R^2^.

**Figure S10.** Distribution of MAE’s of the external validation test set over 5 random seeds for different representations and ML algorithms.

**Figure S11.** Structure of substrates for which enantioselectivity outcomes were failed by 2 kJ mol^-1^ by all the models tested. The predicted value shown corresponds to the ensemble of model created by averaging the result of all the models trained.

# 10. Other asymmetric reactions results

## 10.1 C-H activation

### 10.1.1 Raw data C-H activation

**Table S43.** Raw MAE values of different representation – ML algorithm combinations for scaffold-based splitting trials and 8/2 train/test ratio for the C-H activation dataset.

| **Random seed** | **0** | **1** | **2** | **3** | **4** | **mean ± std** |
| --- | --- | --- | --- | --- | --- | --- |
| RDKit RF MAE mean | 0.698 | 0.702 | 0.718 | 0.667 | 0.684 | 0.69 ± 0.02 |
| ChemBERTa RF MAE mean | 0.719 | 0.752 | 0.796 | 0.744 | 0.741 | 0.75 ± 0.03 |
| Bespoke RF MAE mean | 0.796 | 0.845 | 0.785 | 0.808 | 0.817 | 0.81 ± 0.02 |
| Bespoke XGB MAE mean | 0.776 | 0.803 | 0.77 | 0.888 | 0.826 | 0.81 ± 0.05 |
| RDKit XGB MAE mean | 0.839 | 0.848 | 0.76 | 0.906 | 0.753 | 0.82 ± 0.06 |
| Morgan 1024 RF MAE mean | 0.834 | 1.118 | 0.948 | 0.961 | 1.018 | 0.98 ± 0.10 |
| ChemBERTa XGB MAE mean | 0.981 | 0.854 | 0.786 | 1.399 | 0.874 | 0.98 ± 0.24 |
| Bespoke SVR MAE mean | 1.101 | 0.933 | 0.953 | 1.099 | 0.855 | 0.99 ± 0.11 |
| Morgan 1024 XGB MAE mean | 1.131 | 1.241 | 1.1 | 1.268 | 1.194 | 1.19 ± 0.07 |
| Morgan 1024 SVR MAE mean | 1.1 | 1.455 | 1.245 | 1.27 | 1.37 | 1.29 ± 0.13 |
| Bespoke MLP MAE mean | 1.378 | 0.875 | 1.271 | 1.132 | 2.003 | 1.33 ± 0.42 |
| ChemBERTa SVR MAE mean | 0.799 | 1.82 | 1.69 | 1.622 | 1.218 | 1.43 ± 0.42 |
| Morgan 1024 MLP MAE mean | 2.256 | 1.442 | 1.356 | 0.977 | 1.096 | 1.43 ± 0.50 |
| ChemBERTa MLP MAE mean | 1.144 | 1.051 | 2.594 | 2.107 | 1.147 | 1.61 ± 0.70 |
| RDKit SVR MAE mean | 2.401 | 1.766 | 2.086 | 1.627 | 1.819 | 1.94 ± 0.31 |
| RDKit MLP MAE mean | 2.746 | 1.471 | 1.877 | 2.209 | 1.678 | 2.00 ± 0.50 |
| Graph GIN MAE mean | 4.475 | 4.438 | 1.018 | 2.218 | 3.47 | 3.12 ± 1.49 |

**Table S44.** Raw R^2^ values of different representation – ML algorithm combinations for scaffold-based splitting trials and 8/2 train/test ratio for the C-H activation dataset.

| **Random seed** | **0** | **1** | **2** | **3** | **4** | **mean ± std** |
| --- | --- | --- | --- | --- | --- | --- |
| Morgan 1024 XGB R2 mean | 0.015 | -0.136 | 0.082 | -0.148 | -0.035 | -0.04 ± 0.10 |
| Morgan 1024 SVR R2 mean | -0.008 | -0.541 | -0.096 | -0.238 | -0.372 | -0.25 ± 0.21 |
| Bespoke MLP R2 mean | -0.444 | 0.264 | -0.238 | 0.041 | -1.634 | -0.40 ± 0.74 |
| Morgan 1024 MLP R2 mean | -2.041 | -0.354 | -0.548 | 0.29 | 0.079 | -0.51 ± 0.92 |
| ChemBERTa SVR R2 mean | 0.257 | -1.396 | -1.115 | -0.884 | -0.182 | -0.66 ± 0.68 |
| ChemBERTa MLP R2 mean | -0.062 | 0.167 | -5.247 | -2.78 | -0.178 | -1.62 ± 2.36 |
| RDKit SVR R2 mean | -2.978 | -1.202 | -1.945 | -1.078 | -1.104 | -1.66 ± 0.82 |
| RDKit MLP R2 mean | -3.756 | -0.731 | -1.128 | -2.007 | -0.865 | -1.70 ± 1.25 |
| Graph GIN R2 mean | -10.168 | -10.243 | 0.145 | -2.798 | -7.757 | -6.16 ± 4.65 |
| Morgan 1024 RF R2 mean | 0.342 | -0.033 | 0.269 | 0.24 | 0.173 | 0.20 ± 0.14 |
| ChemBERTa XGB R2 mean | 0.255 | 0.373 | 0.454 | -0.425 | 0.375 | 0.21 ± 0.36 |
| Bespoke SVR R2 mean | 0.123 | 0.381 | 0.36 | 0.042 | 0.453 | 0.27 ± 0.18 |
| Bespoke RF R2 mean | 0.38 | 0.446 | 0.4 | 0.468 | 0.439 | 0.43 ± 0.04 |
| RDKit XGB R2 mean | 0.478 | 0.382 | 0.537 | 0.248 | 0.514 | 0.43 ± 0.12 |
| Bespoke XGB R2 mean | 0.524 | 0.541 | 0.586 | 0.1 | 0.486 | 0.45 ± 0.20 |
| ChemBERTa RF R2 mean | 0.563 | 0.526 | 0.519 | 0.533 | 0.572 | 0.54 ± 0.02 |
| RDKit RF R2 mean | 0.65 | 0.648 | 0.629 | 0.692 | 0.67 | 0.66 ± 0.02 |

### 10.1.2 Complementary figures

**Figure S12.** Distribution of MAE’s of the C-H activation test set over 5 random seeds for different representations and ML algorithms for scaffold-based splitting and 8/2 train/test split ratio.

## 10.2 Asymmetric hydrogenation

### 10.2.1 Raw data asymmetric hydrogenation

**Table S45.** Raw MAE values of different representation – ML algorithm combinations for scaffold-based splitting trials and 8/2 train/test ratio for the asymmetric hydrogenation dataset.

| **Random seed** | **0** | **1** | **2** | **3** | **4** | **mean ± std** |
| --- | --- | --- | --- | --- | --- | --- |
| Bespoke XGB MAE mean | 1.558 | 1.533 | 1.51 | 1.558 | 1.575 | 1.55 ± 0.03 |
| Bespoke RF MAE mean | 1.674 | 1.671 | 1.681 | 1.677 | 1.669 | 1.67 ± 0.00 |
| ChemBERTa RF MAE mean | 1.718 | 1.744 | 1.726 | 1.725 | 1.745 | 1.73 ± 0.01 |
| ChemBERTa XGB MAE mean | 1.751 | 1.708 | 1.748 | 1.72 | 1.798 | 1.74 ± 0.04 |
| RDKit RF MAE mean | 1.863 | 1.774 | 1.774 | 1.785 | 1.791 | 1.80 ± 0.04 |
| RDKit XGB MAE mean | 1.918 | 1.886 | 1.973 | 1.763 | 1.787 | 1.87 ± 0.09 |
| Morgan 1024 SVR MAE mean | 1.849 | 1.978 | 1.849 | 1.819 | 1.931 | 1.89 ± 0.07 |
| Bespoke SVR MAE mean | 1.799 | 1.719 | 2.013 | 1.898 | 2.155 | 1.92 ± 0.17 |
| Bespoke MLP MAE mean | 2.152 | 1.801 | 1.949 | 1.923 | 1.846 | 1.93 ± 0.14 |
| ChemBERTa SVR MAE mean | 2.117 | 1.886 | 1.677 | 1.961 | 2.055 | 1.94 ± 0.17 |
| Morgan 1024 MLP MAE mean | 1.727 | 1.758 | 2.508 | 2.053 | 1.972 | 2.00 ± 0.31 |
| Morgan 1024 XGB MAE mean | 2.105 | 2.027 | 2.076 | 1.931 | 2.007 | 2.03 ± 0.07 |
| Morgan 1024 RF MAE mean | 2.118 | 2.096 | 2.08 | 2.1 | 2.129 | 2.10 ± 0.02 |
| RDKit SVR MAE mean | 1.978 | 2.454 | 2.004 | 1.998 | 2.293 | 2.15 ± 0.22 |
| ChemBERTa MLP MAE mean | 1.757 | 1.671 | 2.552 | 4.885 | 1.707 | 2.51 ± 1.37 |
| Graph GIN MAE mean | 2.157 | 3.522 | 2.873 | 2.487 | 2.116 | 2.63 ± 0.58 |
| RDKit MLP MAE mean | 1351.461 | 123.378 | 781.991 | 512.708 | 735.814 | 701.07 ± 447.21 |

**Table S46.** Raw R^2^ values of different representation – ML algorithm combinations for scaffold-based splitting trials and 8/2 train/test ratio for the asymmetric hydrogenation dataset.

| **Random seed** | **0** | **1** | **2** | **3** | **4** | **mean ± std** |
| --- | --- | --- | --- | --- | --- | --- |
| RDKit SVR R2 mean | 0.179 | -0.696 | 0.124 | 0.065 | -0.039 | -0.07 ± 0.36 |
| ChemBERTa MLP R2 mean | 0.357 | 0.424 | -0.044 | -2.648 | 0.328 | -0.32 ± 1.32 |
| Graph GIN R2 mean | -0.035 | -1.098 | -0.568 | -0.169 | -0.025 | -0.38 ± 0.46 |
| RDKit MLP R2 mean | -inf | -inf | -inf | -inf | -inf | -inf |
| Morgan 1024 RF R2 mean | 0.179 | 0.192 | 0.204 | 0.187 | 0.172 | 0.19 ± 0.01 |
| ChemBERTa SVR R2 mean | 0.012 | 0.194 | 0.394 | 0.192 | 0.183 | 0.20 ± 0.14 |
| Morgan 1024 MLP R2 mean | 0.361 | 0.385 | -0.106 | 0.23 | 0.268 | 0.23 ± 0.20 |
| Bespoke MLP R2 mean | 0.042 | 0.326 | 0.216 | 0.273 | 0.321 | 0.24 ± 0.12 |
| Bespoke SVR R2 mean | 0.312 | 0.315 | 0.2 | 0.284 | 0.132 | 0.25 ± 0.08 |
| Morgan 1024 XGB R2 mean | 0.217 | 0.248 | 0.232 | 0.301 | 0.278 | 0.26 ± 0.03 |
| RDKit RF R2 mean | 0.244 | 0.292 | 0.292 | 0.28 | 0.28 | 0.28 ± 0.02 |
| RDKit XGB R2 mean | 0.261 | 0.285 | 0.237 | 0.35 | 0.341 | 0.29 ± 0.05 |
| Morgan 1024 SVR R2 mean | 0.342 | 0.261 | 0.326 | 0.335 | 0.286 | 0.31 ± 0.03 |
| ChemBERTa RF R2 mean | 0.329 | 0.335 | 0.334 | 0.347 | 0.334 | 0.34 ± 0.01 |
| ChemBERTa XGB R2 mean | 0.373 | 0.365 | 0.341 | 0.393 | 0.328 | 0.36 ± 0.03 |
| Bespoke RF R2 mean | 0.372 | 0.372 | 0.357 | 0.367 | 0.365 | 0.37 ± 0.01 |
| Bespoke XGB R2 mean | 0.432 | 0.429 | 0.453 | 0.43 | 0.425 | 0.43 ± 0.01 |

### 10.2.2 Complementary figures

**Figure S13.** Distribution of MAEs of the asymmetric hydrogenations test set over 5 random seeds for different representations and ML algorithms for scaffold-based splitting and 8/2 train/test split ratio. MAEs from the RDKit MLP pair were excluded for clarity of the visualisation.

# 11. References

1.	The RDKit Documentation — The RDKit 2022.03.1 documentation. https://www.rdkit.org/docs/.

2.	Rogers, D. & Hahn, M. Extended-Connectivity Fingerprints. *J. Chem. Inf. Model.* **50**, 742–754 (2010).

3.	Tsuji, N. *et al.* Predicting Highly Enantioselective Catalysts Using Tunable Fragment Descriptors**. *Angew Chem Int Ed* **62**, e202218659 (2023).

4.	Byadi, S., Gantzer, P., Gimadiev, T. & Sidorov, P. DOPtools: a Python platform for descriptor calculation and model optimization. *Digital Discovery* **4**, 1188–1198 (2025).

5.	PyG Documentation — pytorch_geometric documentation. https://pytorch-geometric.readthedocs.io/en/latest/index.html.

6.	Singh, R. *et al.* ChemBERTa-3: an open source training framework for chemical foundation models. *Digital Discovery* **5**, 662–685 (2026).

7.	Liu, Y. *et al.* RoBERTa: A Robustly Optimized BERT Pretraining Approach. Preprint at https://doi.org/10.48550/arXiv.1907.11692 (2019).

8.	Edwards, C. *et al.* Translation between Molecules and Natural Language. Preprint at https://doi.org/10.48550/arXiv.2204.11817 (2022).

9.	Raffel, C. *et al.* Exploring the Limits of Transfer Learning with a Unified Text-to-Text Transformer. Preprint at https://doi.org/10.48550/arXiv.1910.10683 (2023).

10.	Lu, S., Gao, Z., He, D., Zhang, L. & Ke, G. Data-driven quantum chemical property prediction leveraging 3D conformations with Uni-Mol+. *Nat Commun* **15**, 7104 (2024).

11.	Ziegler, B. E. & McMahon, T. B. Computational analysis of substituent effects and Hammett constants for the ionization of gas phase acids. *Computational and Theoretical Chemistry* **1008**, 46–51 (2013).

12.	Singh, S. *et al.* A unified machine-learning protocol for asymmetric catalysis as a proof of concept demonstration using asymmetric hydrogenation. *Proceedings of the National Academy of Sciences* **117**, 1339–1345 (2020).

13.	Xu, L.-C. *et al.* Enantioselectivity prediction of pallada-electrocatalysed C–H activation using transition state knowledge in machine learning. *Nat. Synth* **2**, 321–330 (2023).

14.	Kipf, T. N. & Welling, M. Semi-Supervised Classification with Graph Convolutional Networks. (2017).

15.	Brody, S., Alon, U. & Yahav, E. How Attentive are Graph Attention Networks? Preprint at https://doi.org/10.48550/arXiv.2105.14491 (2022).

16.	Xu, K., Hu, W., Leskovec, J. & Jegelka, S. How Powerful are Graph Neural Networks? Preprint at https://doi.org/10.48550/arXiv.1810.00826 (2019).

17.	Kingma, D. P. & Ba, J. Adam: A Method for Stochastic Optimization. Preprint at https://doi.org/10.48550/arXiv.1412.6980 (2017).

18.	Lundberg, S. M. & Lee, S.-I. A Unified Approach to Interpreting Model Predictions. in *Advances in Neural Information Processing Systems* (eds Guyon, I. et al.) vol. 30 (Curran Associates, Inc., 2017).

19.	Sundararajan, M., Taly, A. & Yan, Q. Axiomatic Attribution for Deep Networks. *CoRR* vol. abs/1703.01365 (2017).

20.	Degen, J., Wegscheid‐Gerlach, C., Zaliani, A. & Rarey, M. On the Art of Compiling and Using ‘Drug‐Like’ Chemical Fragment Spaces. *ChemMedChem* **3**, 1503–1507 (2008).
